# Supplementary material for: Validation of the QualiPresc instrument for assessing the quality of drug prescription writing in primary health care
Source: PLoS One. 2022 May 11;17(5):e0267707. doi: 10.1371/journal.pone.0267707 (PMC9094502; doi:10.1371/journal.pone.0267707)
Supplement: S1 Table — (DOC) [file pone.0267707.s001.doc]

**S1 Table. Validity analysis of potential indicators.**

| **INDICATOR** | **ROUND 1**  **(n=15)**  **A (%)** | | **ROUND 2**  **(n=15)**  **A (%)** | | **ROUND 3**  **(n=15)**  **A (%)** | | **APPROVAL** |
| --- | --- | --- | --- | --- | --- | --- | --- |
|  | **R** | **A** | **R** | **A** | **R** | **A** |  |
| Number of drugs / prescription^37^ | 66,7 | 60 | 80 | 73,3 | 80 | 80 | NO^a^ |
| Electronic prescription^26^ | 86,7 | 73,3 | 100 | 93,3 | **-** | **-** | YES |
| Absence of erasure^26^ | 86,7 | 93,3 | 100 | 100 | **-** | **-** | YES |
| Patient's full name^26,38^ | 86,7 | 86,7 | 100 | 93,3 | **-** | **-** | YES |
| Patient’s address^26,38^ | 40 | 46,7 | 40 | 46,7 | 46,7 | 33,3 | NO^a^ |
| Patient's date of birth^26^ | 80 | 73,3 | 93,3 | 93,3 | **-** | **-** | YES |
| Prescriber’s full name^26,38^ | 80 | 80 | 93,3 | 86,7 | 93,3 | 86,7 | NO^b^ |
| Prescriber´s identification^26^ | 93,3 | 93,3 | **-** | **-** | **-** | **-** | YES |
| Institution’s name^26^ | 73,3 | 73,3 | 86,7 | 93,3 | 86,7 | 93,3 | NO^c^ |
| Institution’s address^26,38^ | 46,7 | 60 | 60 | 80 | 66,7 | 80 | NO^a^ |
| Date of prescription^26,38^ | 100 | 100 | **-** | **-** | **-** | **-** | YES |
| Use of abbreviations^26^ | 73,3 | 40 | 80 | 53,3 | 93,3 | 66,7 | NO^b^ |
| Use of acronyms/symbols ^26^ | 73,3 | 40 | 73,3 | 46,7 | 86,7 | 66,7 | NO^a^ |
| Drugs with similar names^26^ | 80 | 66,7 | 86,7 | 73,3 | 93,3 | 86,7 | NO^b^ |
| Record of allergy report ^26^ | 86,7 | 93,3 | 100 | 100 | - | - | YES |
| Medicine included in the institutional list officially approved ^26^ | 80 | 73,3 | 93,3 | 86,7 | 100 | 93,3 | YES |
| Active ingredient^26,38,39^ | 73,3 | 86,7 | 93,3 | 93,3 | **-** | **-** | YES |
| Concentration^26,38,39^ | 100 | 100 | - | - | - | - | YES |
| Dosage^26,38,39^ | 100 | 100 | **-** | **-** | **-** | **-** | YES |
| Pharmaceutical form^26,38,39^ | 100 | 100 | **-** | **-** | **-** | **-** | YES |
| Route of administration^26,38,39^ | 100 | 100 | **-** | **-** | **-** | **-** | YES |
| Frequency of administration^26,38,39^ | 100 | 100 | **-** | **-** | **-** | **-** | YES |
| Duration of treatment^26,38^ | 100 | 100 | **-** | **-** | **-** | **-** | YES |
| Directions on the use of the drugs^26,38^ | 100 | 93,3 | **-** | **-** | **-** | **-** | YES |
| Non-pharmacological recommendations^26,38^ | 86,7 | 80 | 100 | 93,3 | - | - | YES |
| Potentially dangerous drugs^26^ | 86,7 | 66,7 | 86,7 | 80 | 93,3 | 86,7 | NO^b^ |
| Vague expression^26^ | 73,3 | 60 | 86,7 | 80 | 93,3 | 86,7 | NO^b^ |
| Drug with narrow therapeutic index ^26^ | 80 | 60 | 80 | 60 | 86,7 | 60 | NO^a^ |
| Drug in therapeutic duplication ^26^ | 80 | 66,7 | 86,7 | 66,7 | 93,3 | 80 | NO^b^ |

A (%): % expert approval

R: relevance; A: adequacy

a R and A <90%; b A <90%; c R <90%

Level III (all potential indicators): Expert opinions based on clinical experience, descriptive studies or expert committee reports (Canadian Task Force on Preventive Health Care).
