# Supplementary material for: Validation of the QualiPresc instrument for assessing the quality of drug prescription writing in primary health care
Source: PLoS One. 2022 May 11;17(5):e0267707. doi: 10.1371/journal.pone.0267707 (PMC9094502; doi:10.1371/journal.pone.0267707)
Supplement: S2 Table — (DOC) [file pone.0267707.s002.doc]

**S2 Table. Reliability and utility analysis of consensual indicators.**

| **INDICATOR** | **RELIABILITY** | | | | | **UTILITY** | **APPROVAL** |
| --- | --- | --- | --- | --- | --- | --- | --- |
|  | **AGREEMENT** | **KAPPA** | | **PREVALENCE** | |  |  |
|  | general (%) | value | CI 95% | AV1  (%) | AV2  (%) |  |  |
| Electronic prescription | 100 | a | a | 0 | 0 | NO | NO^b^ |
| Absence of erasure | 97 | a | a | 100 | 97 | NO | NO^b^ |
| Patient's full name | 100 | a | a | 100 | 100 | NO | NO^b^ |
| Date of prescription | 100 | a | a | 100 | 100 | NO | NO^b^ |
| Patient's date of birth | 100 | a | a | 0 | 0 | YES | YES |
| Prescriber´s identification | 97 | 0,65 | 0,48-0,82 | 93 | 97 | YES | YES |
| Record of allergy report | 100 | a | a | 0 | 0 | YES | YES |
| Medicine included in the institutional list officially approved | 97 | 0,93 | 0,82-1,02 | 67 | 63 | YES | YES |
| Active ingredient | 100 | 1,0 | 1,0-1,0 | 73 | 73 | YES | YES |
| Concentration | 100 | 1,0 | 1,0-1,0 | 70 | 70 | YES | YES |
| Dosage | 87 | 0,73 | 0,57-0,89 | 53 | 67 | YES | YES |
| Pharmaceutical form | 100 | 1,0 | 1,0-1,0 | 77 | 77 | YES | YES |
| Route of administration | 97 | 0,93 | 0,84-1,02 | 40 | 37 | YES | YES |
| Frequency of administration | 97 | 0,65 | 0,48-0,82 | 97 | 93 | YES | YES |
| Duration of treatment | 100 | 1,0 | 1,0-1,0 | 70 | 70 | YES | YES |
| Non-pharmacological recommendations | 97 | a | a | 0 | 3 | YES | YES |
| Directions on the use of the drugs | 90 | 0,67 | 0,50-0,84 | 13 | 23 | YES | YES |

a Kappa does not apply, as data from at least one evaluator was a constant

b Not useful (a- prevalence = 0 and unfeasible to improve; or b- prevalence = 100%)

CI: confidence interval

AV1: rater 1; AV2: rater 2
