# Supplementary material for: Validation of the QualiPresc instrument for assessing the quality of drug prescription writing in primary health care
Source: PLoS One. 2022 May 11;17(5):e0267707. doi: 10.1371/journal.pone.0267707 (PMC9094502; doi:10.1371/journal.pone.0267707)
Supplement: S3 Table — (DOC) [file pone.0267707.s003.doc]

**S3 Table. Definitions and clarifications of the validated indicators.**

| **INDICATOR** | **DEFINITIONS/CLARIFICATIONS** | **S** | **E** | **Q** |
| --- | --- | --- | --- | --- |
| Patient's date of birth^26^ | Day/month/year. | 2,5 | 2,3 | 5,8 |
| Prescriber´s identification^26^ | Covers the professional registration number from the Regional Council, profession, name and surname of the prescriber. The intermediate words to the first name and the last name can be made up of the initial letters. | 2,4 | 1,6 | 3,8 |
| Record of allergy report^26^ | Record of information about the fact that the user is allergic to a certain drug (s). It must be included in the prescription, regardless of whether the drug involved is present in the prescription. | 2,9 | 2,2 | 6,4 |
| Medicine included in the institutional list officially approved^26^ | Component of an institution's standard list of drugs, preferably standardized according to the epidemiological profile and best scientific evidence of efficacy, safety, and cost-effectiveness. Alternatively, the standard list of the essential municipal, state, or national medicines can be used as the basis, instead of the standard list of a specific institution, considering the most appropriate to the context. | 2,1 | 2,1 | 4,4 |
| Active ingredient^26,38,39^ | This is the Brazilian Common Denomination (DCB) or, in its absence, the International Common Denomination (DCI). Short drug names or chemical formulas (e.g., MgSO4) should not be used. Nomenclature should be written in full [26,64]. | 2,4 | 2,2 | 5,3 |
| Concentration^26,38,39^ | Corresponds to the amount of active ingredient contained in each unit dose. For solid dosage forms, 1 unit dose corresponds to 1 unit dosage form (e.g., 1 tablet). For semi-solid and liquid dosage forms, 1 dose unit corresponds to 1 unit of measure (e.g., 1 mL, 1 g). The acronyms U, u, and UI should not be used, as they can be confused with 0, 4, cc, IV, or 10; an international unit should be written instead. The acronyms mcg and µg should not be used; microgram should be written instead. A zero should not be written on the right of a decimal point, nor should the zero on the left of the decimal point be omitted, as this may result in the decimal place going unnoticed: write X mg and 0, X mg, respectively. For doses or volumes with fractional numbers (e.g., 2.5 mL), observe whether the comma is well positioned and clear in the two copies of the prescription. Do not use a period to replace the comma [26,64]. | 2,9 | 2,9 | 8,4 |
| Dosage^26,38,39^ | The amount of medication to be administered at each time of use. Comprised of a numerical value and a unit of measure. As a unit of measure, consider the name of the solid pharmaceutical form, drops, or nationally and/or internationally recommended abbreviations/acronyms/symbols. The acronyms U, u, and UI should not be used, as they can be confused with 0, 4, cc, IV, or 10; an international unit should be written instead. The acronyms mcg and µg should not be used; microgram should be written instead. A zero should not be written on the right of a decimal point, nor should the zero be omitted on the left of the decimal point, as this may result in the decimal place going unnoticed: write X mg and 0, X mg, respectively. For doses or volumes with fractional numbers (e.g., 2.5 mL), observe whether the comma is well positioned and clear in the two copies of the prescription. Do not use a period to replace the comma [26,64]. | 3,0 | 3,0 | 9,0 |
| Pharmaceutical form^26,38,39^ | Final physical form of the drug after mixing active ingredients and excipients during the production process. | 2,9 | 2,8 | 8,1 |
| Route of administration^26,38,39^ | Gateway through which the medication is administered in order to reach its place of action (e.g., oral, intramuscular, intravenous, etc.). Prefer using EV (intravenous) instead of IV (intravenous), due to the risk of misinterpretation of IV as IM [26,64]. | 2,9 | 2,9 | 8,4 |
| Frequency of administration^26,38,39^ | Number of times each dose of medication should be administered, considering a 24-hour period (e.g., every 12 hours, etc.). | 3,0 | 3,0 | 9,0 |
| Duration of treatment^26,38^ | Period of time during which the medicine should be used (e.g., for 10 days, continuous use, etc.). If the prescription contains more than 1 medication, consider it to be in compliance with this item only if all prescription medications meet the indicator. | 2,9 | 3,0 | 8,7 |
| Directions on the use of the drugs^26,38^ | Directions, such as the best period for medication administration (e.g., morning/night, before/after meals, fasting, after bathing), duration of medication administration (e.g., n minutes, until the contents of the container are complete), thin layer application (e.g., topical use), dilution of the medication, instruction for using an inhalation device, among others, that are necessary for the user's understanding of how to use the medication. | 3,0 | 3,0 | 9,0 |
| Non-pharmacological recommendations^26,38^ | Information regarding non-drug treatment (e.g., diet, physical activity, etc.). | 2,1 | 2,3 | 4,8 |
| **COMPOUND INDICATORS** | **CLARIFICATIONS FOR THE MEASURE** | | | |
| Prescription quality | - To evaluate a prescription, the calculation is the sum of the scores of each of the 13 indicators of the QualiPresc instrument, in case of compliance. - For aggregated data, it is the average of the sum found in each prescription. | | | |

S: safety; E: efficacy; Q: quality (composite indicator scores)
