# Supplementary material for: Validation of the QualiPresc instrument for assessing the quality of drug prescription writing in primary health care
Source: PLoS One. 2022 May 11;17(5):e0267707. doi: 10.1371/journal.pone.0267707 (PMC9094502; doi:10.1371/journal.pone.0267707)

QualiPresc - Qualidade da Prescrição na Atenção Primária à Saúde

Instruções:

O instrumento QualiPresc tem o objetivo de facilitar o monitoramento da qualidade da prescrição de medicamentos com base em crtérios de escrita. Entende-se por critérios de escrita aqueles relacionados à estrutura do documento prescrição a partir de informações de usuário, prescritor e medicametos prescritos.

Como utilizar:

1) O instrumento contempla 13 indicadores da qualidade da prescrição de medicamentos que têm diferentes pesos para a efetividade e a segurança da prescrição.
2) O QualiPresc deve ser aplicado, preferencialmente, por farmacêuticos e/ou após treinamento ministrado por este profissional.
3) Antes de coletar os dados, os avaliadores devem ler com muita atenção as definições e os esclarecimentos para a medida dos indicadores no quadro a seguir.
4) Após a leitura, clique na próxima planilha chamada "coleta" ou na seta ao lado para registrar os dados das prescrições avaliadas.

INDICADOR	DEFINIÇÕES/ESCLARECIMENTOS PARA A MEDIDA	SCORE[3]		
		S	E	Q
**1. Data de nascimento do paciente**	Dia / mês / ano.	7,1	6,9	6,4
**2. Identificação do prescritor**	Abrange nº de registro profissional no Conselho Regional, profissão, nome e sobrenomes do prescritor. As palavras intermediárias ao nome e ao último sobrenome podem ser constituídas pelas letras iniciais.	6,9	4,8	4,2
**3. Registro de relato de alergia**	Registro de informação acerca do fato do usuário ser alérgico a determinado (s) medicamento (s). Deve constar na prescrição independentemente do medicamento envolvido estar presente na prescrição.	8,3	6,6	7
**4. Medicamento incluído na lista institucional aprovada oficialmente[1]**	Componente do elenco padrão de medicamentos de uma instituição, padronizados, preferencialmente, conforme perfil epidemiológico e melhores evidências científicas de eficácia, segurança e custo-efetividade. Alternativamente ao elenco padrão de uma instituição em específico, pode-se usar como base o elenco padrão de medicamentos essenciais municipais, estaduais ou nacional, considerando a mais adequada ao contexto.	6	6,3	4,8
**5. Princípio ativo**	Trata-se da Denominação Comum Brasileira (DCB) ou, em sua ausência, da Denominação Comum Internacional (DCI). Não utilizar nomes abreviados	6,9	6,6	5,8
**6. Concentração[2]**	Corresponde à quantidade de princípio ativo contida em cada unidade posológica. Para formas farmacêuticas sólidas, 1 unidade posológica corresponde a 1 unidade de forma farmacêutica (ex. 1 comprimido). Para formas farmacêuticas semi-sólidas e líquidas, 1 unidade posológica corresponde a 1 unidade de medida (ex. 1 ml, 1g) (4,5).	8,3	8,7	9,2
**7. Dose[2]**	Quantidade de medicamento a ser administrada a cada horário de uso. Constituída por valor numérico e unidade de medida. Como unidade de medida, considerar nome da forma farmacêutica sólida ou gotas ou abreviaturas/siglas/símbolos nacional e/ou internacionalmente recomendados (4,5).	8,6	9	9,9
**8. Forma farmacêutica**	Forma física final do medicamento após mistura entre princípios ativos e excipientes por ocasião do processo de produção.	8,3	8,4	8,9
**9. Via de administração**	Porta de entrada através da qual o medicamento é administrado, a fim de alcançar seu local de ação (ex. oral, intramuscular, intravenoso, etc.).	8,3	8,7	9,2
**10. Frequência de administração**	Quantidade de vezes, considerando período de 24h em que cada dose de medicamento deve ser administrada (ex. a cada 12 horas, etc.).	8,6	9	9,9
**11. Duração do tratamento**	Período de tempo durante o qual o medicamento deve ser utilizado (ex. durante 10 dias, uso contínuo, etc.).	8,3	9	9,6
**12. Orientações sobre uso do medicamento[1]**	Melhor período para administração do medicamento (ex. manhã/noite, antes/depois das refeições, jejum, após banho), duração da administração do medicamento (ex. n minutos, até finalizar conteúdo do recipiente), aplicação de fina camada (ex. uso tópico), diluição do medicamento, instrução de uso de dispositivo inalatório, entre outras necessárias para compreensão do usuário acerca da forma de uso do medicamento.	8,6	9	9,9
**13. Recomendações não farmacológicas**	Informações concernentes ao tratamento não medicamentoso (ex. dieta, atividade física, etc.).	6	6,9	5,3

[1] Nos indicadores 4 a 12, se a prescrição apresentar mais de 1 medicamento, considera-se este item conforme apenas se todos os medicamentos da prescrição atenderem ao indicador.

[2] Não utilizar siglas U, u e UI, pois podem confundir com 0 ou 4 ou cc ou IV ou 10, escrever unidade internacional. Não utilizar siglas mcg ou µg, escrever micrograma. Não utilizar zero à direita nem omitir zero à esquerda, pois a casa decimal pode passar despercebida, escrever X mg e 0,X mg, respectivamente. Para doses ou volumes com números fracionados (ex. 2,5mL), observar nas duas vias da prescrição se a vírgula está bem posicionada e clara. Não utilizar ponto em substituição à vírgula.[4,5]

[3] Scores de ponderação com respeito a Segurança (S) e Efetividade (E), para ponderação do indicador composto Qualidade (Q).

[4] The Joint Commission. Facts about Official Do-Not-Use List. Disponível em: http://www.jointcommission.org/assets/1/18/Do_Not_Use_List.pdf. Acesso em: 18 de abril de 2021.

[5] Ministério da Saúde. Protocolo de segurança na prescrição, uso e administração de medicamentos. 2013. Disponível em: https://proqualis.net/sites/proqualis.net/files/000002490IQmwD8.pdf.

QualiPresc - Qualidade da Prescrição na Atenção Primária à Saúde

Coleta de dados:

1. Para garantir um monitoramento contínuo, selecione no mínimo 20 a 30 prescrições. Faça a seleção aleatória (sorteio entre todas prescrições do período avaliado), para garantir a representatividade.
2. Inclua somente prescrições legíveis e coloque o número de identificação das incluídas na primeira coluna após a data de coleta. Se alguma estiver ilegível, sorteie outra prescrição do mesmo período para completar a amostra.
3. Para as prescrições incluídas, responda com 0 ou 1 se os indicadores forem conformes ou não conformes, respectivamente.
4. Após inserir as respostas, confira na seguinte planilha os resultados consolidados dos 13 indicadores simples e dos 3 indicadores compostos do QualiPresc.

IDENTIFICAÇÃO			INDICADORES SIMPLES														
ID	Data	Nº prescrição	1. Data de nascimento do paciente	2. Identificação do prescritor	3. Registro de relato de alergia	4. Medicamento padronizado pela instituição	5. Princípio ativo	6. Concentração	7. Dose	8. Forma farmacêutica	9. Via de administração	10. Frequência de administração	11. Duração do tratamento	12. Orientações sobre uso do medicamento	13. Recomendações não farmacológicas	14. Qualidade	Opções de resposta
1			1	0	1	0	1	0	1	0	1	1	0	1	1	63	0
2			0	0	1	0	0	1	0	1	1	0	0	0	1	40	1
3			0	0	1	1	1	0	0	1	1	1	0	0	0	46	
4			0	0	1	1	1	1	1	0	1	1	1	1	0	75	
5			1	0	1	0	0	1	0	0	0	0	1	1	0	42	
6			0	0	1	0	0	1	1	1	0	0	1	0	0	45	
7			1	0	0	0	1	1	1	0	0	1	1	0	1	56	
8			1	1	0	0	0	0	0	1	1	0	0	1	0	39	
9			1	1	0	1	1	1	0	1	1	0	0	1	1	64	
10			0	0	1	0	1	1	0	0	0	1	1	1	1	57	
11			1	0	1	1	1	0	1	0	0	0	1	1	1	59	
12			0	1	1	1	0	1	0	0	1	0	0	1	1	50	
13			0	1	0	1	1	0	1	1	1	1	0	0	1	58	
14			1	1	1	0	0	0	1	1	0	1	1	0	0	56	
15			1	1	0	0	1	1	0	0	0	1	0	0	1	41	
16			1	1	1	0	0	0	0	1	0	1	1	0	1	51	
17			1	1	0	0	1	1	0	1	0	1	0	1	0	54	
18			1	0	0	0	0	0	1	1	1	0	0	1	0	44	
19			1	1	0	1	1	0	1	0	0	1	1	1	0	61	
20			0	1	0	1	0	0	0	1	1	1	0	0	1	42	
21			0	1	0	1	0	1	1	1	1	0	0	0	0	46	
22			1	0	0	0	0	1	1	0	1	1	1	0	0	54	
23			1	1	0	0	1	0	0	0	0	1	1	0	1	41	
24			1	0	0	0	1	1	0	0	1	0	0	0	0	31	
25			0	1	1	0	0	1	0	0	0	0	1	1	0	40	
26			0	0	0	0	1	1	0	0	1	0	1	1	1	49	
27			1	1	0	0	1	0	0	1	1	0	0	1	0	44	
28			1	1	1	1	0	1	0	0	1	0	0	0	0	41	
29			0	1	1	0	1	0	0	1	1	1	1	0	1	60	
30			0	1	1	0	0	1	1	1	1	1	1	1	1	83	
31			0	1	1	1	1	0	1	0	1	0	0	0	0	41	
32			0	0	1	1	1	0	0	1	1	0	0	1	0	46	
33			0	1	0	0	1	1	1	0	0	1	0	1	1	54	
34			0	1	1	1	1	0	1	1	0	0	1	0	1	56	
35			1	0	0	1	1	1	1	0	1	1	0	1	1	70	
36			1	0	0	0	1	1	0	1	0	1	0	0	1	46	
37			0	1	0	0	1	1	0	1	0	1	1	1	0	58	
38			1	1	1	1	0	0	0	0	0	0	0	1	1	38	
39			1	0	1	1	0	1	0	1	0	0	1	0	1	51	
40			1	1	0	0	1	1	1	1	0	0	0	1	0	54	
41			0	0	0	0	0	1	1	1	0	1	1	1	0	57	
42			1	1	0	0	0	1	0	0	1	0	0	0	1	34	
43			1	0	0	1	0	1	1	0	1	0	1	0	0	49	
44			1	1	1	0	1	0	1	1	1	1	0	0	1	67	
45			1	0	0	1	1	1	1	1	0	0	1	1	1	70	
46			1	1	0	0	0	1	0	1	1	0	0	1	0	48	
47			0	1	1	0	1	0	0	1	0	0	1	0	1	41	
48			0	1	1	1	1	0	0	0	1	0	0	0	0	31	
49			1	0	1	0	1	0	1	1	1	1	1	1	1	82	
50			0	1	1	1	1	1	0	0	0	1	1	0	0	51	
51			1	1	0	1	1	0	1	0	1	0	1	0	0	50	
52			1	1	0	0	1	1	1	1	1	0	1	0	1	69	
53			0	1	0	1	1	0	1	1	1	0	0	0	0	43	
54			1	1	0	1	1	0	1	0	0	0	0	0	0	31	
55			1	1	1	1	0	1	1	0	1	1	1	0	0	70	
56			1	1	1	0	1	0	1	1	0	1	1	0	1	67	
57			1	0	0	1	1	1	0	0	1	0	0	0	1	41	
58			0	1	0	0	1	0	0	1	1	0	1	0	0	38	
59			1	0	1	1	0	1	0	1	1	1	1	0	1	70	
60			1	1	1	1	1	0	0	1	0	0	0	0	0	37	
61			1	0	1	1	1	0	1	1	1	1	0	0	1	67	
62			1	0	1	0	1	0	0	0	1	0	1	0	0	38	
63			0	0	0	1	1	1	0	0	1	0	0	0	1	34	
64			1	1	1	1	1	1	1	0	0	0	1	1	0	67	
65			0	0	1	0	1	0	0	1	1	1	0	1	1	56	
66			1	1	1	1	0	0	0	1	0	0	1	1	0	51	
67			0	0	0	0	0	0	0	1	0	0	1	0	0	19	
68			1	0	1	1	0	0	0	1	1	0	1	1	0	56	
69			1	0	1	0	1	1	0	1	0	0	0	1	0	47	
70			0	0	1	0	0	0	0	0	0	1	1	0	1	32	
71			0	1	0	1	1	1	0	1	0	1	1	1	1	68	
72			1	1	1	1	0	0	0	0	0	1	0	1	1	48	
73			0	1	0	0	1	1	0	1	1	0	1	0	1	52	
74			1	1	1	1	0	1	0	1	1	0	1	0	1	65	
75			1	1	1	0	0	0	1	0	1	0	1	0	0	46	
76			1	0	1	0	0	1	1	0	0	1	1	0	0	52	
77			0	1	0	0	1	1	1	0	1	1	0	0	0	48	
78			0	1	1	0	1	0	1	0	1	0	1	1	0	56	
79			1	0	1	0	1	1	1	1	1	0	0	1	1	72	
80			1	1	1	1	1	1	1	0	1	0	0	1	0	66	
81			1	0	1	1	0	0	1	0	0	0	1	0	1	43	
82			1	0	1	0	1	1	0	1	1	0	0	1	0	56	
83			1	0	1	1	1	1	0	1	0	1	0	0	0	52	
84			1	1	0	0	0	0	0	1	1	1	1	1	0	58	
85			1	1	0	1	0	1	0	0	1	1	0	1	0	54	
86			1	0	0	1	1	0	1	0	1	0	0	1	0	46	
87			1	1	0	0	0	1	1	1	0	0	1	0	1	54	
88			1	0	1	0	1	0	1	1	0	0	1	1	0	58	
89			0	1	1	0	1	0	1	1	1	0	1	0	0	55	
90			0	0	0	1	0	0	1	0	1	1	0	1	1	49	
91			1	1	1	0	0	1	0	0	0	0	1	0	1	42	
92			1	1	0	1	1	0	0	0	1	1	1	0	0	50	
93			1	1	1	1	0	1	0	0	0	0	0	1	0	42	
94			1	1	1	0	0	1	1	1	0	0	1	1	0	65	
95			1	1	1	0	1	1	0	1	1	0	1	1	0	70	
96			0	0	1	1	1	1	1	1	1	1	1	0	1	80	
97			0	1	1	0	1	1	0	0	0	1	0	1	1	51	
98			1	1	0	1	1	1	1	0	1	1	0	1	0	69	
99			0	0	0	1	0	0	1	0	0	0	1	1	0	34	
100			1	1	1	0	0	1	0	0	1	0	0	0	1	41	
101			0	1	1	1	0	0	0	0	1	0	0	1	0	35	
102			1	1	0	0	1	0	1	1	0	1	1	1	0	65	
103			1	0	1	0	1	0	0	1	1	0	0	0	0	37	
104			1	0	1	1	1	1	0	0	0	1	1	1	1	68	
105			0	0	0	0	0	0	1	0	1	1	0	0	1	34	
106			0	0	0	1	1	1	1	0	1	1	1	0	0	58	
107			1	1	0	1	1	1	1	1	0	0	0	0	0	49	

108			0	0	1	1	1	1	1	0	1	1	1	0	1	71
109			1	0	0	1	0	0	0	0	1	1	1	1	0	50
110			1	1	1	1	1	0	0	0	0	1	1	0	0	48
111			1	0	0	1	1	1	0	0	0	1	1	1	0	56
112			0	1	0	1	1	0	1	1	1	1	1	0	1	68
113			1	1	0	0	1	0	0	0	1	1	0	0	1	41
114			1	0	1	1	1	1	1	1	1	0	0	0	1	67
115			1	1	1	1	0	1	0	1	1	1	0	0	1	65
116			1	1	0	0	1	0	0	1	1	0	0	1	1	50
117			1	1	1	1	1	0	1	0	1	1	1	1	0	77
118			1	1	1	1	0	1	0	1	0	1	1	0	0	60
119			0	1	0	0	1	0	1	0	1	0	0	0	0	29
120			1	0	1	0	0	1	0	0	1	0	1	1	0	51
121			0	0	1	1	1	0	1	1	1	0	1	1	0	65
122			0	1	1	1	1	0	0	0	0	0	1	1	0	41
123			1	0	0	1	1	0	0	0	1	1	1	0	0	46
124			0	1	1	0	1	1	1	1	0	1	0	1	0	65
125			1	1	0	1	1	1	0	1	1	1	0	1	1	74
126			0	1	1	1	0	0	0	0	1	1	0	1	0	45
127			0	0	1	1	0	0	0	0	1	1	0	1	1	46
128			1	0	1	0	0	1	1	1	0	1	1	0	1	66
129			0	1	1	0	1	1	1	0	0	0	1	0	0	46
130			1	1	0	0	0	0	1	0	0	1	1	0	0	40
131			1	1	1	1	1	1	0	0	0	1	0	1	0	57
132			1	0	1	0	0	1	0	1	1	1	0	0	1	56
133			1	0	0	0	0	1	1	1	1	1	0	0	0	54
134			0	1	0	1	0	0	0	1	0	0	0	1	1	33
135			0	0	1	1	1	1	0	0	1	1	0	1	0	56
136			0	1	0	0	1	0	1	1	1	0	1	1	1	63
137			1	1	1	0	0	1	0	0	0	1	1	1	1	62
138			0	1	1	0	0	1	0	0	0	1	0	0	0	30
139			0	1	0	1	0	0	0	0	0	1	0	1	0	29
140			0	0	1	0	1	1	0	1	1	0	0	1	0	50
141			1	1	1	0	0	0	0	1	0	0	1	0	0	36
142			0	0	0	0	1	1	0	1	0	1	0	1	1	49
143			0	0	1	0	0	0	0	1	1	1	1	0	1	50
144			0	0	0	1	0	1	0	1	1	0	0	1	1	47
145			1	1	0	1	1	1	0	1	1	1	0	1	1	74
146			0	1	0	0	1	1	0	0	1	1	0	1	1	54
147			1	0	0	1	0	0	1	1	0	1	0	1	0	50
148			0	0	0	0	0	0	1	1	0	0	1	0	0	28
149			1	0	0	1	1	1	0	0	1	1	1	1	0	65
150			0	0	0	1	0	0	1	1	1	1	1	1	0	62
151			1	0	1	0	0	0	0	0	1	1	1	0	1	47
152			1	1	0	1	0	1	0	1	0	1	0	1	1	59
153			1	0	1	1	1	0	1	1	1	0	0	1	0	62
154			0	1	1	0	1	0	1	1	1	0	1	1	0	65
155			0	1	0	0	0	0	1	1	0	0	0	0	0	23
156			0	0	1	0	1	1	1	0	1	1	0	1	1	66
157			0	0	0	1	0	0	0	0	1	0	1	0	1	29
158			0	1	0	1	0	1	0	1	0	1	0	1	0	47
159			1	0	1	1	0	1	0	1	1	1	0	0	0	55
160			0	1	0	1	0	1	0	0	0	1	1	1	0	48
161			1	0	0	0	0	0	1	1	0	1	1	1	1	60
162			0	1	0	1	1	0	1	1	0	0	0	0	1	39
163			1	1	0	1	0	0	1	1	1	0	1	0	1	58
164			1	1	1	1	1	1	0	1	0	0	1	1	1	71
165			0	0	0	0	0	0	1	0	1	1	1	1	0	49
166			0	1	0	0	0	0	0	0	0	0	0	0	1	10
167			0	1	1	0	0	0	1	1	0	1	0	0	0	40
168			0	0	1	0	1	1	1	1	1	0	0	0	1	55
169			0	1	0	0	1	0	1	1	0	0	0	0	0	29
170			1	0	0	0	1	1	0	0	1	1	0	1	0	50
171			1	0	1	0	0	0	0	0	0	1	1	1	1	48
172			1	1	1	0	0	1	0	1	0	1	0	0	0	46
173			1	1	0	0	1	0	1	0	1	1	0	0	1	51
174			1	1	0	0	0	1	0	1	0	1	1	1	0	58
175			1	0	1	1	0	1	0	0	0	0	1	1	0	47
176			0	0	1	1	1	0	0	0	0	0	1	0	1	33
177			1	0	0	0	0	1	0	1	1	1	1	1	0	63
178			1	1	0	1	0	1	1	1	1	1	0	1	0	72
179			1	1	0	0	0	1	0	0	0	0	1	1	0	39
180			0	1	1	1	1	1	1	1	1	1	1	1	1	94
181			1	0	0	1	1	0	1	0	0	1	0	0	0	37
182			1	1	0	0	0	0	1	1	1	0	0	0	0	39
183			0	1	0	1	1	1	1	1	0	0	1	0	1	58
184			0	0	1	0	0	0	0	0	0	1	0	0	0	17
185			1	0	0	1	0	1	1	0	1	1	1	1	0	69
186			0	0	0	0	0	0	1	1	0	1	1	1	1	54
187			1	1	0	1	0	1	0	0	1	1	0	0	0	44
188			1	0	1	1	1	0	0	1	0	1	1	0	0	52
189			1	1	0	1	1	1	1	1	0	1	1	1	1	84
190			1	0	0	1	1	0	0	0	1	1	0	0	1	41
191			0	1	0	1	0	1	0	1	1	0	0	0	0	36
192			0	0	1	0	1	0	1	0	1	1	0	1	0	52
193			1	0	0	0	0	0	1	1	0	1	1	0	0	45
194			0	1	0	0	0	0	1	1	0	1	0	0	0	33
195			1	1	0	0	1	1	0	1	1	1	0	1	0	64
196			0	0	0	0	0	0	1	1	0	0	0	0	0	19
197			0	1	1	0	0	0	0	0	1	1	1	1	0	50
198			0	1	1	0	0	1	0	0	0	0	0	0	0	20
199			1	1	1	1	1	1	1	1	1	1	0	1	0	85
200			0	0	1	1	1	1	0	1	0	0	1	1	0	55
201			1	0	0	0	1	1	1	0	1	0	0	0	1	46
202			1	1	1	1	0	0	1	0	1	0	0	1	0	51
203			0	0	1	1	1	1	1	1	0	1	1	1	0	75
204			1	0	1	0	0	1	0	0	1	0	1	0	1	47
205			0	0	0	0	0	1	1	0	1	0	1	1	0	48
206			1	0	0	0	0	1	1	1	1	1	0	0	1	59
207			1	0	0	1	1	1	1	1	1	1	0	1	0	74
208			1	1	0	0	0	1	1	1	1	1	0	1	0	68
209			0	1	1	1	1	1	1	1	0	0	1	0	0	59
210			0	1	1	1	0	0	1	1	0	1	1	1	0	64
211			1	1	1	1	1	0	0	0	0	1	0	1	1	53
212			0	1	1	1	1	0	0	0	0	0	1	1	1	47
213			1	1	0	0	1	1	1	0	1	0	0	0	1	50
214			1	0	0	0	1	1	0	1	1	1	0	0	1	55
215			0	0	0	0	1	1	0	0	0	0	0	0	0	15
216			0	1	1	1	1	1	0	1	0	1	1	1	0	69
217			0	0	0	1	0	1	0	0	1	1	1	1	1	58
218			1	0	1	0	1	1	1	0	0	1	0	0	1	54
219			1	0	0	0	1	1	0	1	0	0	1	0	0	40
220			1	1	0	0	0	0	0	0	1	1	0	0	0	30
221			1	0	0	1	1	1	0	1	0	1	0	0	1	50
222			0	0	0	0	1	1	1	1	0	1	1	0	1	59
223			1	1	0	0	0	1	0	0	0	1	1	0	0	39
224			1	1	0	1	0	1	1	1	1	1	0	1	1	78
225			0	1	0	0	1	1	1	1	1	1	1	1	0	77

226			1	0	1	1	0	1	1	0	1	1	1	0	0	66
227			1	1	0	0	0	1	1	1	0	0	1	1	0	58
228			1	1	0	1	0	1	0	0	1	0	0	1	1	49
229			0	0	0	0	1	0	0	0	1	0	1	1	1	40
230			1	1	0	1	1	1	1	0	0	1	1	0	0	60
231			0	0	1	1	0	0	0	1	1	1	1	0	1	55
232			1	1	1	1	1	0	1	1	0	0	0	1	1	62
233			0	0	0	1	0	0	1	0	1	0	0	0	0	24
234			1	1	0	1	0	1	0	1	1	0	0	1	1	58
235			0	0	1	0	1	1	1	1	1	1	1	1	0	79
236			1	1	0	1	1	1	0	0	0	1	0	0	0	40
237			1	0	1	1	1	0	0	1	0	0	0	0	0	33
238			0	0	1	0	1	1	0	1	0	0	1	1	0	50
239			1	1	0	1	1	0	0	1	1	1	0	1	0	59
240			1	1	0	0	0	0	1	1	1	0	0	0	1	44
241			1	1	1	0	1	1	0	0	0	1	0	0	0	43
242			0	0	0	0	0	1	0	1	1	1	1	0	0	47
243			0	0	0	1	1	1	1	0	1	1	0	1	1	64
244			0	0	0	0	0	1	0	1	1	0	0	1	1	43
245			0	1	0	1	0	1	1	1	1	1	1	1	1	81
246			1	0	0	1	0	1	1	1	0	1	0	1	1	64
247			1	1	0	1	0	1	1	1	0	1	0	1	1	69
248			0	0	0	1	0	1	1	0	0	0	0	0	0	24
249			0	0	0	0	0	0	0	1	1	0	0	1	1	33
250			0	0	1	1	0	0	0	0	0	1	0	1	0	32
251			1	1	0	1	0	1	1	0	0	0	1	1	0	54
252			1	1	1	0	0	0	0	0	0	1	1	1	0	47
253			1	0	1	0	1	0	1	0	1	0	0	0	1	44
254			0	1	1	0	0	1	1	1	0	1	1	0	1	64
255			1	1	0	0	1	1	1	0	1	0	0	1	0	55
256			0	1	1	1	1	0	0	1	0	1	0	0	1	46
257			0	0	0	0	0	1	1	1	0	1	0	0	0	38
258			0	0	1	1	1	0	1	0	1	0	0	1	1	52
259			1	1	0	0	0	1	0	0	0	0	1	0	0	29
260			1	1	0	1	1	0	0	0	0	0	1	0	1	36
261			1	1	0	1	0	1	1	0	0	0	0	1	0	44
262			0	0	1	0	1	0	1	1	0	1	0	1	1	57
263			0	0	0	0	0	0	0	1	1	0	0	0	0	18
264			1	1	1	1	0	1	1	0	1	1	0	0	0	61
265			0	1	0	1	0	1	0	0	0	1	1	1	1	53
266			1	1	0	0	1	1	0	0	1	1	0	1	0	55
267			0	0	1	1	1	1	1	0	0	1	1	1	1	71
268			1	1	0	1	1	0	1	0	1	1	1	0	1	65
269			1	1	1	1	0	1	0	0	1	0	1	1	1	66
270			1	1	1	1	0	1	0	1	1	0	1	0	1	65
271			1	1	0	0	0	1	0	0	0	0	0	0	1	25
272			0	0	0	1	0	1	0	1	1	1	0	0	0	42
273			0	0	0	1	1	1	1	0	0	0	1	1	0	49
274			0	0	1	0	1	0	0	0	0	1	0	1	0	33
275			1	1	0	1	1	0	1	0	1	1	1	0	0	60
276			1	1	0	0	0	1	0	1	0	1	0	1	1	54
277			1	1	1	0	1	0	1	0	1	1	0	0	1	58
278			0	1	1	0	0	1	0	1	0	0	0	0	1	35
279			0	0	0	0	1	1	1	0	0	0	0	1	0	35
280			0	1	1	0	0	0	0	0	0	0	0	0	0	11
281			0	0	0	0	1	1	1	1	0	1	1	0	1	59
282			1	0	0	0	0	1	1	1	0	1	0	0	0	44
283			1	0	0	0	0	1	1	0	0	0	0	1	0	35
284			1	0	0	1	0	1	1	1	0	1	0	1	1	64
285			1	0	1	1	1	0	1	0	0	0	1	1	1	59
286			0	0	1	1	0	1	1	0	1	0	0	1	1	55
287			1	0	0	1	1	1	1	0	1	1	0	0	0	55
288			0	0	0	0	0	0	0	1	1	0	1	0	1	33
289			0	1	0	1	0	1	1	1	0	1	0	1	0	57
290			0	0	0	1	1	0	1	0	1	0	0	0	1	35
291			0	1	1	1	0	0	0	1	0	0	0	0	0	25
292			1	0	0	0	0	1	0	1	1	1	0	1	0	54
293			0	0	1	1	1	0	1	1	0	0	1	1	0	56
294			1	0	1	0	0	0	0	0	1	0	1	0	0	32
295			0	1	1	1	0	1	1	1	1	1	0	0	1	68
296			1	1	1	0	0	0	0	0	0	0	0	1	1	33
297			1	1	1	0	0	1	1	0	1	0	0	0	0	46
298			1	0	0	0	1	0	0	1	1	1	1	1	1	65
299			1	0	0	1	1	1	0	0	0	1	1	0	1	51
300			1	0	1	0	1	0	1	0	0	0	1	1	1	54
301			0	0	1	0	1	1	0	1	0	0	1	0	0	41
302			0	0	0	1	1	1	1	1	0	0	1	0	0	48
303			1	0	0	0	1	1	0	1	1	0	0	0	1	45
304			1	0	1	1	1	0	1	1	0	0	1	0	1	58
305			0	0	0	0	1	1	1	0	1	1	0	0	0	44
306			0	0	0	1	0	0	1	0	0	1	1	0	0	34
307			0	1	0	1	0	1	1	1	0	0	0	0	0	37
308			1	0	1	1	0	0	0	0	0	1	0	0	0	28
309			0	0	0	0	0	0	0	0	0	0	0	0	0	0
310			0	0	1	1	1	0	0	0	1	0	0	1	0	37
311			0	0	0	1	1	1	1	0	1	1	1	0	0	58
312			1	1	1	0	0	1	1	1	1	1	1	1	1	90
313			0	0	0	0	1	0	0	1	0	1	1	1	0	44
314			0	1	1	0	1	0	1	1	0	1	1	1	1	71
315			0	0	1	0	1	1	0	1	1	1	0	0	0	50
316			1	1	1	1	0	1	1	0	1	1	0	1	0	71
317			0	1	0	1	0	1	0	0	0	1	0	0	1	33
318			1	1	0	0	1	1	1	1	0	0	1	1	1	69
319			0	0	0	1	0	0	0	1	0	1	1	0	1	39
320			0	1	0	1	1	0	1	1	1	1	0	1	1	68
321			1	1	0	0	0	0	1	1	0	1	0	0	1	45
322			1	1	1	1	0	0	0	1	1	1	1	1	0	70
323			1	1	1	0	1	1	0	0	0	0	0	0	0	33
324			0	1	0	1	0	1	0	1	1	1	0	0	0	46
325			1	0	1	0	1	0	0	1	1	1	0	1	0	57
326			1	0	0	1	1	1	0	1	0	1	1	0	1	60
327			1	1	1	0	0	0	1	0	1	0	0	1	1	52
328			1	0	1	0	0	1	0	0	0	0	1	1	1	47
329			1	0	0	1	1	0	1	0	1	0	1	1	0	56
330			1	1	0	1	0	1	0	1	1	0	0	0	0	43
331			0	1	0	1	1	0	0	0	1	0	1	1	0	44
332			0	1	1	0	1	1	0	0	0	1	0	0	0	36
333			0	0	0	1	1	0	1	0	1	0	1	0	1	45
334			0	0	0	1	0	1	1	1	1	0	1	1	0	62
335			0	0	0	0	1	1	0	0	1	0	0	1	1	39
336			0	1	1	0	1	0	1	1	1	0	1	1	0	65
337			0	1	0	0	0	0	1	0	1	0	1	1	0	43
338			1	0	0	0	1	0	0	1	0	0	0	1	0	31
339			1	1	1	0	0	0	0	0	1	0	1	1	1	52
340			1	0	1	0	1	1	1	0	1	1	0	1	1	73
341			0	1	1	1	0	1	1	1	0	0	1	0	0	54
342			0	1	1	0	0	1	0	0	0	0	0	1	1	36
343			0	0	1	1	1	0	0	0	0	1	1	0	1	42

344			0	1	0	0	0	0	1	1	1	0	0	1	1	47
345			0	1	0	0	1	0	0	0	1	0	0	1	0	29
346			0	0	0	0	0	1	1	1	0	0	1	1	1	53
347			0	0	0	1	1	0	0	0	1	1	1	0	1	45
348			0	0	0	0	1	1	0	0	1	1	1	0	0	44
349			0	1	1	0	0	1	0	1	1	1	1	0	1	63
350			1	0	0	1	0	0	1	1	1	0	1	0	0	49
351			1	0	1	0	1	0	1	0	0	1	0	0	0	39
352			1	0	0	0	0	0	1	1	0	0	0	1	0	35
353			0	0	0	0	0	1	1	0	0	0	1	0	1	34
354			1	0	1	1	0	1	1	1	0	0	0	0	0	46
355			1	0	0	1	0	0	1	0	1	1	1	1	0	60
356			1	1	1	1	1	0	0	0	0	0	1	0	1	43
357			0	1	1	1	1	1	0	1	0	1	0	0	1	55
358			0	1	0	1	0	1	0	1	0	1	0	1	0	47
359			0	1	0	1	1	0	1	1	1	0	1	0	1	58
360			0	0	0	1	0	0	0	1	0	1	1	0	1	39
361			1	1	0	1	1	0	0	0	0	1	0	1	0	41
362			1	1	1	1	1	0	0	1	0	0	1	1	0	57
363			1	1	1	0	1	0	1	0	0	0	0	1	0	43
364			1	0	0	0	0	1	1	0	0	1	0	0	0	35
365			1	1	0	1	1	1	0	1	1	0	1	0	1	63
366			1	0	0	1	1	1	1	1	1	1	1	1	0	84
367			1	0	1	0	1	1	1	1	0	0	0	0	0	47
368			1	1	0	0	1	0	0	0	1	1	1	1	0	55
369			1	1	0	0	0	1	0	0	0	0	0	0	1	25
370			1	0	1	0	0	1	1	0	1	0	0	0	1	47
371				1	1	1	1	0	1	0	1	1	1	1	0	70
372				1	0	1	1	1	0	0	1	0	1	1	0	53
373				0	0	1	1	0	1	0	0	1	1	1	0	50
374				1	0	1	1	1	0	0	0	0	1	1	1	49
375				1	0	0	0	0	0	1	0	1	0	1	1	38
376				0	1	0	1	0	0	1	0	0	1	0	1	37
377				0	1	0	0	0	1	1	1	0	1	0	0	45
378				0	0	1	0	0	0	1	1	0	0	1	0	33
379				1	1	1	1	1	1	0	0	1	0	1	1	66
380				1	0	0	1	0	1	1	1	1	1	0	0	58
381				0	1	1	1	1	1	1	0	1	0	0	1	61
382				0	0	0	1	0	1	0	0	1	1	1	1	50
383				1	1	0	1	0	1	1	0	1	1	1	0	65
384				0	1	1	1	0	1	0	1	1	1	1	0	66
385				0	0	0	1	0	0	0	1	0	0	0	0	15
386				0	0	1	1	0	0	1	1	1	0	1	0	49
387				0	1	1	1	0	1	1	0	0	1	1	0	56
388				0	0	0	0	0	0	1	0	1	1	1	1	44
389				1	0	0	1	1	0	1	0	0	1	1	0	48
390				0	1	1	0	1	0	0	1	1	0	1	1	55
391				1	1	0	0	1	1	0	0	1	0	0	1	46
392				1	1	0	1	1	0	0	1	1	0	1	1	61
393				1	1	0	1	0	1	0	0	0	0	0	1	32
394				0	1	1	1	1	0	0	1	1	0	1	0	56
395				0	1	0	1	1	1	1	0	0	1	1	1	66
396				0	0	0	1	1	0	0	0	0	1	1	0	35
397				1	0	0	0	0	0	1	1	0	1	1	0	42
398				0	0	0	1	0	0	1	0	0	0	1	1	30
399				0	0	0	0	1	0	0	1	0	0	1	0	28
400				1	1	1	1	0	1	1	0	0	0	1	0	51
401				0	1	1	1	1	1	0	0	0	1	0	0	46
402				0	1	1	1	1	1	0	0	1	0	0	1	52
403				0	0	1	0	0	0	0	1	1	1	1	1	49
404				0	1	1	1	1	1	0	1	1	0	0	0	56
405				0	0	1	1	1	0	0	0	0	0	1	1	35
406				1	1	0	1	0	0	1	0	1	0	0	0	36
407				1	1	1	0	1	1	1	1	0	0	1	1	68
408				1	1	0	1	0	0	1	1	1	1	1	1	70
409				1	1	0	1	0	0	0	1	0	1	1	1	51
410				1	0	1	0	0	0	0	1	0	0	1	0	28
411				0	0	1	0	1	0	0	1	0	1	0	0	33
412				0	0	1	1	0	1	1	1	1	0	1	1	64
413				0	0	0	0	1	1	0	0	0	0	0	0	19
414				0	1	1	0	0	1	1	1	1	1	0	0	59
415				0	1	1	1	1	0	1	0	0	1	1	1	61
416				0	1	1	0	1	0	1	0	1	0	1	0	50
417				1	0	1	1	1	1	0	1	1	1	1	1	78
418				1	1	0	0	1	1	0	1	0	0	0	1	45
419				1	0	1	0	0	0	1	1	1	1	0	0	47
420				0	1	1	0	0	1	0	1	1	1	0	1	56
421				0	1	0	1	1	0	1	1	0	1	0	0	50
422				1	0	0	1	1	1	0	1	1	1	1	1	73
423				1	0	1	0	1	1	1	1	1	1	0	1	71
424				1	0	1	1	0	1	1	1	1	0	1	0	63
425				0	0	1	1	0	0	1	1	0	1	1	1	54
426				0	0	0	1	0	0	1	0	0	1	1	1	40
427				0	1	0	0	0	0	0	1	1	1	0	0	36
428				0	1	1	0	0	0	0	0	0	1	1	1	37
429				1	1	0	0	0	1	0	0	0	0	0	1	26
430				0	0	0	0	0	0	0	0	0	0	0	1	5
431				1	0	0	1	0	0	1	1	0	1	1	0	48
432				1	0	0	0	1	1	1	1	0	1	0	1	56
433				1	1	1	1	1	1	1	1	1	1	1	1	94
434				1	1	0	0	0	1	0	0	0	0	1	1	36
435				1	1	1	0	0	1	1	0	1	1	0	1	60
436				1	0	0	0	1	1	0	0	0	1	0	1	38
437				1	1	1	0	1	0	0	0	1	0	0	1	40
438				1	1	0	0	0	0	1	1	1	0	0	1	45
439				1	1	0	1	1	1	0	0	1	0	0	0	46
440				1	1	1	0	0	1	0	0	1	1	1	0	55
441				0	1	0	1	1	1	1	1	0	0	1	0	60
442				1	1	0	1	1	0	1	0	1	0	0	1	50
443				1	0	1	0	1	0	0	0	1	1	0	0	38
444				0	0	1	1	1	0	0	1	0	0	0	0	29
445				1	0	1	0	0	0	1	1	1	0	0	1	42
446				0	1	0	0	0	1	1	0	1	0	1	0	46
447				1	0	1	1	1	0	0	1	1	0	1	1	58
448				0	1	1	0	1	1	1	1	1	1	0	0	69
449				1	0	0	0	0	1	0	0	0	0	1	0	24
450				0	0	1	0	1	0	1	0	1	0	0	0	33
451				1	1	1	1	1	0	0	0	0	0	0	1	36
452				1	0	0	1	0	0	1	0	1	1	0	1	44
453				0	0	1	0	1	0	1	0	0	1	1	1	48
454				0	0	0	1	1	0	0	0	0	0	1	0	25
455				0	1	0	0	0	0	0	1	0	1	1	0	36
456				0	0	0	1	0	1	1	1	0	0	1	1	49
457				1	0	1	0	1	1	0	1	0	0	1	1	53
458				1	0	0	0	0	0	1	0	0	1	1	1	38
459				0	1	1	1	0	0	0	1	1	0	1	0	47
460				1	1	1	1	1	1	0	0	1	0	0	0	51
461				0	0	1	0	0	1	1	1	0	1	1	1	58

462				0	1	1	1	0	1	0	1	0	1	0	0	46
463				0	1	0	1	1	1	0	0	0	0	0	1	37
464				1	0	0	1	1	1	0	0	1	0	0	0	39
465				0	0	1	0	0	1	1	1	1	1	0	1	58
466				0	0	1	0	0	1	1	0	1	1	1	1	58
467				0	0	1	1	1	1	1	0	0	1	1	0	58
468				1	1	1	1	1	1	1	1	1	1	1	0	88
469				1	1	0	0	0	1	1	0	1	0	1	0	50
470				0	1	1	0	0	0	0	1	1	1	0	0	41
471				0	1	1	0	0	1	0	0	1	0	0	0	32
472				0	1	0	0	1	0	0	0	1	1	0	1	41
473				0	1	0	1	1	1	1	1	1	1	1	1	85
474				1	1	1	1	1	0	0	1	0	1	0	1	55
475				0	1	1	1	0	1	0	1	1	0	0	1	52
476				1	1	0	0	1	0	0	1	0	1	1	1	54
477				0	0	0	1	1	0	0	1	0	1	0	1	39
478				1	1	0	1	0	1	0	1	1	1	1	0	66
479				1	0	0	0	0	0	0	1	0	0	1	0	23
480				1	0	0	0	1	0	1	1	1	1	0	0	51
481				0	1	0	0	0	1	1	1	1	0	1	1	60
482				0	0	0	0	1	1	1	1	1	1	0	1	62
483				1	1	1	0	0	0	1	0	0	1	1	0	44
484				1	0	0	0	0	0	1	0	1	1	1	1	48
485				0	1	1	1	1	1	0	0	0	0	0	0	37
486				1	0	1	1	0	0	0	0	1	0	0	1	30
487				0	0	1	1	1	0	0	0	1	0	1	1	45
488				1	1	1	1	0	1	0	0	0	1	0	0	41
489				1	1	1	0	1	1	1	0	1	0	1	1	69
490				0	1	0	1	1	0	0	0	1	1	0	0	42
491				1	0	0	0	1	0	0	1	0	0	0	0	23
492				1	1	0	0	0	0	1	0	0	0	0	1	25
493				1	0	0	1	0	1	0	0	0	0	1	0	30
494				1	0	0	0	1	1	0	1	1	0	0	1	48
495				1	1	1	0	0	0	0	0	0	1	0	0	26
496				0	0	1	0	1	0	1	1	0	1	0	1	47
497				1	0	1	0	0	1	0	0	0	1	1	1	44
498				0	1	1	1	1	0	1	1	1	1	1	0	74
499				0	0	0	1	0	1	0	0	1	1	1	1	50
500				0	0	1	1	0	1	0	1	0	0	0	1	35
501				0	1	0	1	0	0	1	0	0	0	1	1	37
502				0	1	0	1	0	1	0	0	1	0	0	0	33
503				0	1	1	0	0	0	0	0	0	1	1	0	31
504				0	1	0	1	1	1	0	0	1	0	1	1	57
505				1	0	0	0	1	1	0	0	0	1	0	0	33
506				1	0	1	0	1	1	0	1	0	1	1	1	62
507				0	1	1	0	0	1	0	1	0	0	1	0	41
508				0	1	1	1	0	1	1	0	1	1	0	1	61
509				1	1	1	0	1	0	1	1	1	1	0	0	63
510				0	1	0	0	1	0	0	1	0	1	0	1	40
511				0	0	0	1	1	0	0	0	1	1	1	1	50
512				0	1	1	0	1	0	0	0	1	0	0	1	36
513				0	0	0	1	1	1	1	1	0	1	1	1	68
514				0	1	1	1	1	0	1	0	0	1	1	1	61
515				0	1	1	0	1	0	1	1	0	1	0	0	49
516				1	0	1	1	0	0	0	1	1	1	1	0	53
517				0	0	0	1	0	1	0	0	1	0	1	1	41
518				1	0	1	0	1	0	0	0	0	0	1	1	33
519				1	1	0	1	1	1	1	1	1	0	0	0	64
520				0	1	0	1	0	0	1	1	0	1	0	0	41
521				0	1	1	0	0	0	0	0	0	1	1	1	37
522				1	0	1	1	0	1	1	0	0	0	0	1	39
523				1	1	1	1	0	0	1	0	0	0	0	0	31
524				1	1	1	1	1	1	1	0	0	1	1	0	69
525				1	1	0	0	0	0	1	0	0	1	0	0	30
526				0	0	1	1	1	0	1	0	0	1	1	1	54
527				1	0	1	0	0	1	0	0	1	0	1	0	39
528				0	0	1	1	0	0	0	0	1	1	1	0	40
529				0	1	1	0	0	0	0	0	1	0	1	0	32
530				1	0	1	0	0	1	1	0	1	0	1	1	53
531				1	1	0	0	1	0	1	1	0	0	1	1	54
532				0	0	0	1	0	1	1	1	0	1	0	0	43
533				0	1	0	0	0	0	0	0	0	1	1	0	27
534				1	1	1	0	0	1	1	1	1	0	0	0	54
535				0	1	0	0	1	1	0	1	0	1	1	1	60
536				0	1	0	1	1	1	0	1	0	1	1	1	66
537				1	0	0	1	0	1	1	0	1	0	0	0	39
538				0	0	1	1	0	1	1	1	0	1	1	1	63
539				1	1	0	1	1	1	0	0	0	0	0	1	41
540				1	1	1	0	0	1	0	1	1	1	0	1	60
541				0	1	1	1	1	0	1	0	0	1	1	0	55
542				0	0	1	1	1	0	1	1	1	1	0	0	57
543				0	1	1	1	0	0	0	0	0	0	0	0	18
544				1	0	0	1	0	1	0	1	0	1	1	0	49
545				1	0	1	0	0	1	0	0	1	1	1	1	54
546				1	0	1	1	0	0	0	1	0	1	0	0	34
547				0	1	1	1	1	1	0	1	0	0	0	1	51
548				1	0	1	1	0	0	0	1	0	0	1	1	39
549				1	0	0	1	1	0	1	0	1	1	1	0	58
550				1	0	1	1	1	0	0	1	0	0	0	0	33
551				1	1	1	0	1	0	0	1	1	1	0	1	59
552				0	0	1	0	0	0	0	0	0	1	1	0	24
553				0	0	0	0	0	0	0	1	0	1	0	0	19
554				0	1	1	0	0	0	1	1	1	1	0	0	49
555				1	1	1	0	1	1	0	1	0	0	0	0	44
556				0	0	0	1	0	0	1	1	0	0	0	1	29
557				1	0	0	1	1	1	1	0	1	1	1	1	73
558				0	1	1	0	0	1	1	1	0	1	0	1	55
559				1	0	1	0	1	0	0	1	0	0	0	1	33
560				1	1	0	0	0	1	1	1	0	1	0	0	49
561				1	0	0	0	0	1	1	1	1	0	1	1	57
562				0	1	1	0	0	1	0	0	0	0	0	0	22
563				1	1	0	1	1	1	1	0	0	0	0	1	50
564				0	0	0	1	1	1	0	1	1	0	1	1	59
565				1	1	0	0	0	0	1	1	1	0	0	0	39
566				0	0	1	1	1	0	0	0	1	0	1	0	40
567				1	0	0	1	1	1	0	1	1	1	0	0	58
568				1	0	1	0	1	0	1	1	1	0	0	1	52
569				0	1	1	1	1	0	0	1	0	0	1	1	51
570				0	1	1	1	1	0	1	1	1	1	1	1	80
571				0	0	0	1	1	0	0	1	0	1	0	1	39
572				1	1	0	0	0	0	0	1	1	1	0	1	45
573				1	1	1	1	0	0	0	1	1	1	0	1	56
574				1	1	1	1	1	1	0	1	0	0	0	0	50
575				0	1	0	1	0	0	0	1	1	1	1	0	51
576				1	1	0	0	0	1	0	0	1	1	0	0	41
577				1	0	0	1	1	0	0	1	0	1	0	0	38
578				0	0	0	0	0	0	1	1	0	1	0	0	28
579				0	1	1	1	0	1	1	1	1	1	0	1	70

580				1	0	1	1	1	0	0	0	0	0	1	1	39
581				1	1	1	0	0	1	0	0	1	0	0	0	36
582				0	0	1	0	1	0	0	0	0	0	1	0	24
583				1	0	0	1	0	1	0	1	1	1	1	0	59
584				0	1	0	0	1	0	1	1	0	1	1	1	59
585				1	0	0	1	0	1	0	1	1	1	0	0	49
586				1	0	1	0	1	1	1	1	1	0	0	0	56
587				0	0	1	0	1	1	1	1	0	0	0	1	47
588				0	1	0	1	1	0	0	0	1	0	0	1	37
589				0	0	0	1	1	1	0	1	1	1	1	1	69
590				0	1	1	0	1	0	1	0	1	1	0	0	49
591				1	0	1	0	1	1	0	0	0	0	0	1	33
592				0	0	1	0	0	1	1	0	0	1	1	1	48
593				1	1	1	1	1	1	0	0	0	0	1	1	56
594				0	1	1	1	0	0	0	1	0	1	0	0	36
595				1	1	1	1	0	1	0	0	1	1	1	0	61
596				1	0	1	0	1	0	0	1	0	1	1	0	47
597				1	1	0	0	1	1	1	0	1	1	0	0	59
598				0	0	1	0	1	0	1	1	1	0	0	0	42
599				1	0	1	1	0	1	1	1	1	0	1	1	68
600				1	0	0	0	1	1	1	1	0	0	1	1	57
601				0	1	1	0	1	0	0	1	1	1	1	1	65
602				1	1	0	1	1	0	1	1	1	0	1	1	69
603				0	1	1	1	0	0	1	1	1	0	1	0	56
604				1	0	1	0	1	1	1	1	1	1	1	1	81
605				0	0	1	0	0	0	1	1	0	0	0	0	23
606				0	1	0	0	1	1	1	1	0	0	1	1	59
607				0	0	0	0	0	1	1	1	1	0	0	0	38
608				1	1	1	0	0	1	1	1	0	0	1	1	59
609				1	0	0	0	1	1	0	0	0	1	1	1	48
610				0	0	0	0	1	1	1	1	0	1	0	0	47
611				1	1	1	0	0	0	0	0	0	1	0	0	26
612				1	1	1	1	0	1	0	1	0	0	1	1	56
613				0	1	1	0	1	0	0	1	0	1	0	0	40
614				0	0	1	0	1	1	0	1	0	1	0	1	48
615				1	0	0	0	1	0	0	0	0	1	0	1	28
616				0	0	1	1	0	0	0	1	1	0	1	1	45
617				1	1	0	0	1	0	1	0	0	1	0	1	44
618				0	1	1	0	0	1	1	0	0	1	1	1	55
619				1	0	1	0	0	1	0	1	0	1	0	0	38
620				0	0	1	1	0	0	0	1	1	0	0	0	30
621				1	1	1	1	1	1	1	0	0	0	0	0	50
622				0	1	1	1	0	1	0	0	1	1	0	0	47
623				0	1	0	1	0	0	0	1	0	0	1	1	37
624				1	1	0	0	0	1	1	0	0	0	0	1	35
625				0	1	0	0	1	1	1	0	0	1	1	0	55
626				1	0	1	1	1	1	1	0	1	1	0	0	62
627				1	1	0	0	1	1	1	0	0	0	1	1	54
628				1	0	1	1	0	1	1	0	1	0	0	0	44
629				0	0	0	0	1	1	1	0	1	1	0	0	48
630				0	0	1	0	0	0	1	1	1	0	0	0	33
631				0	0	1	0	1	1	0	1	1	0	0	0	43
632				0	1	0	0	1	0	0	0	0	1	0	0	26
633				0	0	0	0	1	0	0	1	1	0	0	0	28
634				1	1	1	1	1	0	0	1	0	1	0	1	55
635				0	1	0	0	0	0	0	1	0	1	0	1	31
636				1	0	0	1	1	0	1	1	0	0	0	1	43
637				0	0	1	1	1	0	1	0	0	0	1	1	44
638				1	0	0	1	0	1	1	1	1	0	0	0	48
639				1	1	0	0	0	1	1	0	1	0	0	1	45
640				0	0	1	0	1	0	0	1	0	1	1	0	43
641				1	0	1	1	1	1	0	1	1	0	1	1	68
642				0	1	0	0	0	1	1	1	0	0	0	1	40
643				1	0	1	1	1	0	1	1	0	1	0	1	57
644				0	1	1	1	1	0	0	1	1	1	1	1	71
645				1	1	1	1	1	0	0	1	1	0	0	1	55
646				0	1	0	0	1	0	0	1	1	0	0	0	35
647				0	0	1	0	0	0	0	0	0	1	1	1	30
648				1	1	0	0	1	0	1	0	0	1	0	0	39
649				1	1	1	1	1	0	1	1	1	1	1	0	79
650				0	0	1	1	1	1	0	1	0	0	0	1	44
651				1	1	1	0	0	0	0	0	1	1	1	1	51
652				0	1	0	0	1	0	0	1	0	0	0	1	31
653				1	1	1	0	0	0	0	1	1	0	1	1	50
654				1	1	0	0	0	1	0	1	0	0	1	0	40
655				1	0	0	0	1	0	1	0	1	0	0	1	38
656				1	1	0	1	1	1	0	1	0	1	0	0	55
657				0	1	0	0	0	1	0	0	0	0	1	0	27
658				1	0	1	1	1	1	1	1	1	1	1	1	87
659				1	0	0	0	1	1	0	1	1	0	1	0	52
660				1	1	0	1	0	0	0	1	0	1	0	0	36
661				1	1	1	0	1	0	1	0	1	0	1	1	59
662				0	0	0	1	0	1	0	1	0	0	1	1	40
663				0	0	0	1	1	1	0	0	0	0	1	1	40
664				1	0	0	1	0	1	1	0	0	1	0	0	38
665				0	0	0	1	0	1	0	0	1	1	1	1	50
666				1	0	1	1	1	0	0	0	1	0	1	1	49
667				0	1	0	0	1	0	1	0	1	1	0	1	50
668				0	0	1	0	0	0	1	0	1	1	0	0	33
669				1	1	1	0	0	0	1	1	0	0	0	0	34
670				1	1	1	1	0	0	1	1	0	0	0	1	45
671				0	0	1	0	0	0	1	0	0	0	0	0	14
672				0	0	0	0	0	1	0	0	1	1	1	0	39
673				0	0	1	0	0	1	0	0	0	1	1	1	40
674				0	1	1	0	1	1	0	1	1	1	1	1	75
675				0	1	0	1	1	1	0	1	1	1	0	1	66
676				0	0	1	0	1	0	0	0	0	0	0	1	19
677				1	1	1	1	1	1	0	0	1	0	1	1	66
678				1	0	1	0	1	1	0	0	0	1	0	1	43
679				0	1	0	1	1	1	1	0	0	1	0	1	56
680				0	1	0	1	1	1	0	0	1	0	1	0	52
681				1	1	0	1	0	1	0	0	1	0	0	1	42
682				1	0	1	0	1	0	1	1	0	1	1	1	61
683				1	0	1	0	0	1	1	1	0	0	1	1	52
684				1	0	0	1	1	0	1	0	0	0	0	0	28
685				1	0	0	1	0	0	1	0	0	1	1	0	38
686				1	0	1	1	1	0	1	0	0	0	0	1	38
687				0	0	1	1	1	0	1	0	1	0	0	0	39
688				0	0	1	1	0	1	0	0	0	0	1	0	30
689				1	1	1	0	1	1	1	1	0	1	1	1	78
690				1	1	1	0	1	0	0	0	1	1	0	0	45
691				1	0	0	1	1	1	1	0	0	1	1	1	63
692				0	0	0	1	0	0	1	0	1	1	1	1	49
693				0	1	0	0	0	0	1	1	0	0	1	1	40
694				1	1	0	1	0	0	0	0	0	1	1	1	42
695				1	1	0	0	0	0	1	0	1	1	0	0	40
696				1	1	0	1	1	0	0	0	0	0	1	1	41
697				1	1	1	1	0	1	0	0	1	0	0	1	47

698				0	1	0	1	0	0	1	1	1	1	0	0	50
699				1	0	0	0	0	1	1	0	1	1	1	1	58
700				1	0	1	1	1	0	1	0	0	0	1	1	48
701				0	1	1	0	0	0	1	1	0	1	0	1	45
702				0	0	1	1	0	0	0	1	0	1	0	1	35
703				1	0	1	1	1	0	0	0	0	1	0	0	34
704				1	0	0	0	1	0	0	0	0	0	1	1	29
705				1	1	1	1	1	0	0	1	1	0	1	0	60
706				0	0	0	1	1	1	0	1	0	0	0	0	34
707				0	0	0	1	0	1	0	0	1	0	1	0	36
708				0	0	0	1	0	0	1	0	1	0	1	1	40
709				1	0	0	0	0	1	0	0	1	1	0	0	34
710				1	1	0	0	0	1	1	1	0	1	1	1	64
711				1	0	0	1	0	1	0	1	0	0	0	0	29
712				0	1	1	0	1	1	1	0	1	0	0	0	50
713				1	1	0	1	1	1	1	0	1	0	1	1	70
714				1	0	0	1	1	1	0	0	0	1	1	1	54
715				0	0	0	0	0	1	1	1	0	0	1	1	43
716				1	0	0	1	0	1	1	0	0	0	0	1	34
717				1	1	0	0	0	0	0	1	0	0	0	1	26
718				0	1	1	1	1	0	0	0	0	0	1	0	37
719				0	0	1	1	0	0	1	1	1	0	0	0	39
720				0	1	1	0	1	1	1	1	1	1	0	1	74
721				0	0	0	1	0	1	1	0	1	1	0	0	44
722				1	0	0	1	1	1	1	0	0	1	0	1	53
723				0	0	1	1	1	0	0	1	0	1	0	0	39
724				0	0	0	0	0	0	1	0	1	0	0	1	24
725				1	1	0	0	0	0	0	0	0	0	1	0	21
726				1	1	0	1	1	0	1	1	0	0	1	0	54
727				0	0	1	0	1	0	0	0	0	1	1	0	34
728				1	0	1	0	0	1	0	0	1	0	0	0	29
729				0	1	0	0	1	0	0	0	0	0	0	1	22
730				0	1	1	1	0	1	0	0	1	1	0	0	47
731				1	1	1	0	0	1	0	0	1	0	0	1	41
732				1	0	0	1	0	0	0	0	1	1	0	1	35
733				0	1	1	1	0	0	0	0	1	0	0	0	28
734				0	0	1	0	1	0	0	1	1	0	1	0	43
735				1	0	0	1	1	0	1	1	0	1	0	0	47
736				0	0	1	1	1	0	0	1	1	1	1	1	64
737				1	0	0	0	1	1	1	1	1	1	1	0	71
738				1	1	1	0	0	1	0	1	1	1	1	0	65
739				0	1	1	1	0	1	1	0	0	0	0	1	42
740				1	0	0	0	0	0	1	1	0	0	1	0	32
741				1	1	1	0	1	1	0	1	1	0	0	0	54
742				1	0	0	1	0	1	0	0	1	1	0	1	45
743				1	0	1	1	1	1	0	0	0	0	0	0	34
744				1	1	1	1	0	1	1	1	1	0	1	0	70
745				1	1	1	1	1	1	1	1	1	1	1	1	94
746				1	0	0	1	1	0	1	0	0	1	0	1	43
747				1	1	1	0	0	1	1	1	0	1	1	1	69
748				0	0	1	0	0	1	1	1	1	1	0	0	52
749				0	1	1	1	1	0	0	1	1	0	1	1	61
750				0	0	1	1	0	0	1	1	0	1	0	1	44
751				1	0	0	1	1	1	0	1	1	1	1	1	73
752				0	0	1	0	1	0	0	0	1	0	0	1	29
753				0	1	1	0	0	0	0	0	0	1	1	0	31
754				0	0	0	1	0	0	0	1	0	1	0	1	30
755				1	0	0	0	1	1	1	0	1	1	1	1	67
756				0	0	1	0	1	0	1	1	1	0	0	1	47
757				0	0	1	0	0	1	1	1	1	1	1	1	68
758				1	1	0	0	1	1	0	1	1	1	1	1	74
759				1	0	0	1	1	1	0	0	1	0	1	0	49
760				1	0	0	1	1	1	1	1	0	1	0	1	62
761				1	0	1	1	0	0	0	0	0	1	1	0	34
762				1	1	1	1	0	0	0	0	1	0	0	0	32
763				0	0	1	0	1	1	0	0	1	1	0	1	49
764				1	0	1	0	1	1	1	0	0	1	1	1	62
765				1	1	0	1	1	0	1	1	0	1	1	1	69
766				0	1	0	0	1	0	1	1	1	0	1	0	54
767				0	0	1	0	0	1	1	0	1	0	1	0	43
768				0	0	0	1	0	1	1	0	0	0	1	1	40
769				0	1	1	0	1	0	0	0	0	1	0	1	36
770				1	1	0	1	1	0	0	0	0	1	0	0	36
771				1	1	0	1	1	0	0	1	0	1	1	1	60
772				0	0	0	1	0	1	0	1	0	0	0	0	25
773				1	0	0	1	1	0	1	0	0	0	1	1	43
774				1	1	0	0	1	1	1	0	1	1	1	1	74
775				1	0	1	1	1	1	1	0	0	1	0	0	52
776				1	1	1	1	0	1	1	0	1	0	1	1	66
777				0	1	0	0	1	0	0	1	1	1	1	0	55
778				0	0	1	1	1	0	0	1	1	1	1	0	58
779				1	0	1	0	0	0	0	0	1	1	1	0	38
780				1	1	1	0	0	1	0	0	0	1	0	1	41
781				1	1	0	1	1	0	1	0	1	0	0	1	50
782				0	0	1	1	1	1	0	1	1	0	1	0	59
783				1	0	0	0	1	0	1	0	0	1	0	1	37
784				0	0	1	0	0	0	0	0	1	1	0	0	24
785				0	1	1	1	1	0	1	0	0	1	1	1	61
786				1	0	1	1	0	0	0	1	1	0	0	0	34
787				0	0	0	1	1	1	0	0	0	0	0	1	30
788				1	1	0	0	0	0	0	1	0	0	1	1	36
789				0	1	0	0	1	1	1	1	0	1	0	1	59
790				1	1	1	0	0	0	1	1	0	0	0	0	34
791				1	0	0	1	1	1	1	0	1	0	0	0	48
792				0	1	1	0	1	1	0	0	0	1	1	0	50
793				0	1	1	0	0	0	0	1	1	0	1	1	46
794				1	1	0	1	0	0	0	1	0	0	0	1	32
795				0	1	0	0	0	1	1	0	1	0	1	0	46
796				0	0	0	0	1	0	0	0	1	1	1	1	44
797				0	1	0	1	1	1	0	0	0	1	1	0	51
798				0	1	0	0	1	0	1	1	0	0	1	1	50
799				0	1	1	0	1	0	0	0	1	0	0	0	31
800				0	1	0	1	0	0	1	1	0	1	0	0	41
801				0	1	0	0	0	0	1	0	0	0	0	1	21
802				0	1	1	0	0	0	0	1	0	1	1	0	41
803				0	0	0	1	1	1	1	0	1	0	1	1	59
804				0	1	0	1	1	1	1	0	0	1	0	1	56
805				1	0	1	0	1	1	0	0	0	0	0	0	28
806				1	1	0	0	0	0	0	0	0	1	1	1	36
807				1	1	1	1	1	1	0	1	1	1	0	0	70
808				1	0	1	0	1	1	0	1	0	0	0	1	43
809				1	1	1	1	1	1	1	1	1	0	0	1	74
810				1	0	0	0	0	1	0	1	0	1	0	1	38
811				1	0	1	0	1	0	1	1	0	0	0	0	36
812				0	0	1	0	1	0	0	1	0	1	1	0	43
813				0	1	1	0	0	1	1	1	1	1	1	1	75
814				1	0	1	1	0	0	0	1	0	0	1	1	39
815				1	1	0	0	1	0	0	1	1	0	0	1	45

816				1	1	0	0	1	1	1	0	1	0	1	0	59
817				1	1	0	1	0	0	0	0	0	1	1	1	42
818				1	1	0	0	1	0	0	0	1	1	1	1	55
819				1	0	0	0	1	0	1	1	0	1	0	1	46
820				0	1	1	1	0	1	1	1	0	0	1	1	61
821				0	1	0	0	0	0	1	1	0	1	0	0	35
822				1	0	1	1	0	1	1	1	0	0	0	0	43
823				1	0	1	1	0	0	0	1	1	0	0	1	39
824				1	0	0	1	1	0	1	1	0	1	0	0	47
825				0	0	0	0	0	0	0	1	1	0	1	0	29
826				1	0	0	1	1	0	1	0	0	0	0	1	33
827				1	0	0	0	1	0	0	0	0	1	0	1	28
828				1	1	1	1	1	0	1	1	0	1	1	1	74
829				0	0	1	1	1	1	0	1	0	0	1	1	54
830				0	1	1	1	1	0	1	0	1	0	1	0	56
831				0	0	0	1	1	1	0	1	1	0	0	1	49
832				0	1	0	0	1	1	0	0	0	1	0	1	41
833				1	0	1	1	1	1	1	0	0	0	0	0	43
834				1	0	1	1	0	0	0	1	1	0	1	0	44
835				0	1	1	1	0	0	0	1	0	1	1	0	46
836				0	0	0	1	0	1	0	1	0	1	0	1	40
837				0	0	0	1	0	1	1	0	1	0	0	1	40
838				1	0	1	1	1	0	1	1	1	1	1	1	77
839				1	0	1	1	1	1	1	0	1	0	0	0	53
840				1	0	1	0	1	1	0	0	1	1	0	0	48
841				1	1	1	1	1	1	1	1	0	0	0	1	64
842				0	0	1	1	0	0	0	1	1	1	1	1	55
843				0	0	0	1	0	1	1	0	0	1	0	0	34
844				0	1	0	1	0	1	1	0	0	0	0	1	37
845				1	0	0	1	1	1	0	1	1	0	1	1	63
846				1	0	1	1	0	0	0	1	1	0	0	0	34
847				0	0	1	1	1	0	0	0	0	1	1	0	39
848				1	0	1	1	1	0	0	1	0	0	0	0	33
849				1	0	0	0	0	0	0	0	1	0	1	0	24
850				0	1	1	0	0	0	0	0	0	1	0	1	27
851				1	0	0	0	0	1	0	0	0	0	1	1	29
852				1	1	0	1	1	1	0	1	0	0	1	0	55
853				1	0	1	1	1	0	0	1	1	1	0	0	53
854				1	0	0	0	1	1	1	0	1	1	1	1	67
855				0	0	0	1	1	0	1	0	1	0	1	1	49
856				0	0	1	0	0	1	1	1	0	0	1	1	48
857				1	1	1	1	0	1	0	1	0	1	0	1	56
858				0	0	1	0	0	1	1	0	1	1	1	1	58
859				1	1	0	1	1	1	1	1	1	0	1	1	79
860				1	1	1	0	1	0	0	1	1	0	0	0	44
861				0	0	1	0	0	1	0	0	1	1	1	0	44
862				0	1	1	1	1	1	0	0	0	1	1	1	62
863				1	1	1	0	1	1	0	1	0	1	0	0	54
864				0	1	0	0	1	1	1	1	0	0	0	0	44
865				0	0	1	1	1	0	0	0	0	1	0	1	35
866				0	1	1	1	0	0	0	1	0	0	0	1	32
867				1	0	0	1	0	1	1	0	0	0	0	1	34
868				0	0	0	0	0	1	1	1	1	0	0	0	38
869				1	0	1	1	0	0	0	1	0	1	1	0	44
870				0	0	1	0	1	0	0	0	1	1	1	1	49
871				1	0	0	1	1	1	0	1	0	1	0	0	48
872				0	1	1	1	1	0	0	1	1	0	1	0	56
873				0	0	1	0	1	1	1	1	1	0	0	0	52
874				1	1	1	0	1	1	0	0	0	0	1	1	50
875				1	0	0	0	1	1	0	1	1	0	1	1	58
876				1	1	0	1	1	1	0	1	0	1	0	0	55
877				0	0	0	0	1	1	1	1	0	1	1	1	62
878				0	0	0	1	1	0	0	0	0	1	0	1	30
879				0	1	1	0	1	1	1	0	0	1	1	1	65
880				0	0	1	0	1	0	0	0	1	0	1	0	34
881				0	1	0	1	0	1	1	0	1	0	1	0	51
882				1	1	0	1	0	0	0	1	0	0	1	0	36
883				0	0	1	1	0	1	1	1	1	0	0	1	54
884				0	0	1	0	0	0	1	0	0	0	0	0	14
885				1	1	1	0	1	1	0	0	0	1	1	0	55
886				1	1	1	1	0	1	0	0	1	0	1	1	57
887				0	1	1	1	0	0	1	0	0	0	0	0	27
888				1	0	0	0	1	0	0	0	0	0	0	1	19
889				1	1	0	1	1	1	0	1	0	1	0	1	60
890				1	0	0	1	0	1	0	0	0	0	1	1	35
891				1	0	1	0	1	1	1	0	0	1	0	0	47
892				0	1	0	1	0	1	1	0	0	0	1	1	47
893				1	1	0	0	1	1	0	0	0	1	1	0	50
894				0	1	1	0	1	0	0	0	1	0	1	0	41
895				1	1	1	1	0	1	0	0	1	0	0	1	47
896				1	1	0	1	0	1	1	1	0	0	1	0	55
897				1	0	0	1	0	0	0	0	0	1	0	1	25
898				1	1	1	1	0	1	0	0	1	1	0	1	57
899				1	1	1	0	1	0	0	0	1	0	0	0	35
900				0	0	1	0	0	1	0	1	1	0	1	1	49
901				1	0	1	1	1	1	1	0	1	0	1	1	68
902				0	1	1	1	1	1	0	0	0	1	0	1	52
903				1	1	1	0	0	1	0	1	1	0	0	1	50
904				0	1	1	1	0	1	0	1	1	0	0	0	47
905				0	1	1	1	1	0	1	1	0	0	0	0	45
906				0	1	1	0	1	1	1	0	0	1	0	0	49
907				0	1	1	0	1	0	1	1	1	1	0	1	64
908				0	1	0	0	0	0	0	1	1	0	1	0	36
909				1	0	0	1	1	1	1	0	1	0	0	1	53
910				0	0	0	1	1	0	0	0	1	1	1	1	50
911				0	1	1	0	1	0	0	0	1	1	0	1	46
912				0	1	1	1	1	0	0	0	1	0	0	1	42
913				0	0	1	0	1	0	1	0	0	1	0	1	38
914				0	1	0	1	0	0	0	0	0	0	1	0	23
915				1	0	1	1	1	0	1	0	1	0	1	0	53
916				1	0	1	0	0	0	0	1	0	0	1	1	33
917				0	1	0	0	0	0	0	1	0	1	0	0	26
918				1	1	0	0	0	1	0	1	0	0	0	1	36
919				1	1	0	1	1	1	0	1	1	0	0	1	61
920				1	1	0	0	0	0	0	1	1	0	0	1	36
921				0	0	0	0	0	1	1	1	1	1	0	1	53
922				0	0	0	0	0	1	1	0	0	1	1	0	38
923				1	0	0	1	1	1	0	1	0	1	1	1	63
924				0	0	1	0	0	1	0	1	0	0	1	1	39
925				0	1	0	0	0	1	1	0	1	0	1	0	46
926				1	0	0	0	1	0	0	0	1	1	0	0	33
927				0	1	0	0	0	1	1	1	0	0	1	1	50
928				1	0	1	1	1	1	1	0	1	0	0	1	58
929				0	0	0	1	0	0	0	0	0	0	0	1	11
930				0	0	1	1	1	1	1	0	0	0	1	0	49
931				1	0	0	1	1	0	0	0	1	1	0	1	44
932				0	1	0	0	0	1	1	0	1	1	1	0	55
933				0	0	1	1	0	1	1	1	0	0	0	0	39

934				0	1	1	0	0	1	1	1	0	0	1	0	50
935				0	0	0	1	0	1	0	1	0	1	0	1	40
936				0	0	0	1	1	0	1	0	1	0	1	0	44
937				1	0	0	1	1	0	1	1	0	0	1	0	47
938				1	1	0	1	1	1	1	1	1	0	1	0	74
939				0	1	0	0	1	1	1	0	0	1	1	0	55
940				1	0	0	1	1	0	0	0	1	0	0	0	29
941				0	1	1	0	1	0	0	1	1	1	1	1	65
942				0	1	0	0	1	1	0	1	0	0	0	1	41
943				0	0	0	1	1	1	1	0	0	0	1	0	44
944				0	0	1	0	1	1	0	1	0	1	0	1	48
945				1	1	1	1	1	0	1	0	0	1	0	1	55
946				1	0	1	0	0	0	0	0	1	1	1	0	38
947				1	1	0	1	1	1	1	0	0	0	1	1	60
948				0	1	0	1	1	1	1	1	0	0	1	0	60
949				1	1	1	1	0	1	0	1	0	0	1	0	51
950				1	0	1	1	1	0	1	1	1	1	1	1	77
951				1	0	1	1	1	0	1	1	0	0	1	1	57
952				1	0	1	0	1	1	1	1	0	0	1	0	56
953				0	0	1	0	1	0	1	0	0	1	1	1	48
954				0	0	1	0	1	1	1	0	0	1	0	1	48
955				0	0	0	1	0	0	1	0	0	0	1	1	30
956				1	0	1	1	0	0	0	1	1	0	1	0	44
957				0	0	0	1	0	0	1	0	1	1	0	0	34
958				1	0	1	1	0	0	0	1	1	0	0	0	34
959				1	0	1	1	1	0	0	1	0	0	0	0	33
960				0	1	1	1	0	0	0	1	1	0	0	0	37
961				1	1	1	0	0	1	0	1	0	1	0	0	45
962				1	0	1	1	0	0	1	1	0	1	0	1	48
963				0	1	0	0	1	1	0	1	0	1	1	1	60
964				0	0	0	0	1	1	0	0	0	1	1	0	39
965				1	0	1	1	0	0	1	1	1	1	1	1	68
966				1	1	0	0	1	1	1	0	0	1	0	0	49
967				0	1	0	0	0	1	0	1	0	0	1	0	36
968				0	0	0	0	1	0	1	0	1	0	0	1	33
969				1	1	1	0	1	0	0	1	0	0	0	0	34
970				1	1	1	1	0	1	0	0	1	0	1	1	57
971				1	1	1	1	1	0	1	0	0	0	1	0	50
972				0	0	0	0	1	1	0	1	1	1	1	1	63
973				0	0	0	1	1	1	1	1	1	0	0	1	58
974				0	1	1	0	1	1	1	1	1	0	0	1	64
975				1	1	1	1	0	1	1	1	1	0	1	0	70
976				1	1	0	0	1	1	0	0	1	0	0	0	40
977				1	0	0	0	1	0	0	0	1	0	0	0	23
978				1	1	1	1	0	1	0	0	0	1	0	1	47
979				1	1	1	0	0	1	1	0	1	0	0	0	45
980				0	1	1	0	0	0	1	1	1	1	0	0	49
981				0	1	0	1	1	1	0	1	0	1	0	0	51
982				1	1	1	1	0	0	1	1	1	1	0	0	59
983				1	0	1	0	1	1	0	1	0	1	0	1	52
984				1	1	1	1	0	1	0	1	0	1	1	0	60
985				1	0	1	1	1	0	1	0	1	1	1	0	62
986				0	0	1	1	0	0	1	0	1	1	1	0	49
987				1	0	1	0	1	0	0	1	1	1	1	0	57
988				0	0	0	1	1	1	0	1	0	0	0	0	34
989				1	0	0	0	0	1	1	0	1	0	1	1	48
990				0	1	0	0	1	0	0	0	1	1	1	1	51
991				1	0	1	1	0	0	1	0	0	0	0	1	29
992				0	0	0	1	1	1	0	1	0	1	0	0	44
993				0	1	1	0	1	0	1	1	1	1	1	1	74
994				1	1	1	0	1	1	0	1	1	1	1	1	79
995				1	0	0	1	1	1	1	0	1	0	1	0	58
996				1	1	1	1	0	0	0	0	0	1	0	1	37
997				0	0	1	0	1	0	1	0	1	0	0	0	33
998				1	1	1	1	1	0	1	0	0	0	0	1	45
999				0	0	0	0	1	0	1	1	1	1	0	1	52
1000				0	1	0	0	1	1	1	0	1	1	1	0	64
1001				0	1	0	1	1	0	1	1	1	1	0	1	65
1002				0	1	1	1	0	1	0	0	0	0	1	0	37
1003				0	0	1	1	1	1	1	0	1	0	1	1	64
1004				1	0	0	1	0	1	0	0	0	1	0	0	30
1005				1	1	0	1	0	0	1	1	0	1	1	1	60
1006				1	0	0	1	0	1	1	0	1	0	1	1	54
1007				1	1	0	1	1	1	1	0	1	1	0	0	65
1008				0	1	1	0	0	1	1	0	1	0	0	0	41
1009				1	0	0	0	0	0	1	0	0	1	1	0	33
1010				1	0	0	1	1	0	1	1	1	1	0	1	62
1011				1	1	1	1	0	0	1	1	1	0	1	0	60
1012				0	1	1	1	1	0	0	0	1	1	1	1	62
1013				0	0	0	0	1	1	1	0	1	0	1	0	48
1014				0	1	1	0	0	1	0	1	1	1	1	1	66
1015				1	1	1	1	0	1	1	1	0	1	0	1	65
1016				1	1	0	0	0	1	1	0	0	0	1	0	40
1017				1	1	0	0	1	0	0	1	1	1	0	0	49
1018				1	1	1	1	0	0	0	1	1	1	1	1	66
1019				1	1	0	1	1	1	0	0	1	1	1	0	66
1020				0	1	1	1	0	1	0	1	1	1	1	1	71
1021				0	1	1	1	0	0	0	0	0	1	1	1	42
1022				1	0	1	0	1	0	1	1	0	1	1	0	56
1023				1	0	1	0	0	1	1	1	0	0	1	0	47
1024				1	0	0	1	1	1	0	1	0	0	0	1	44
1025				1	0	1	0	0	0	1	0	0	1	1	0	37
1026				0	0	1	1	0	1	1	0	1	1	1	0	59
1027				0	0	0	0	1	0	1	0	1	1	1	1	53
1028				1	0	0	1	1	0	0	1	1	1	1	1	63
1029				0	0	0	0	1	1	0	1	0	0	0	1	34
1030				0	1	1	0	0	1	1	0	0	1	0	0	40
1031				1	1	1	0	1	0	0	0	0	0	0	0	25
1032				1	1	1	1	0	0	0	0	1	1	1	1	57
1033				0	1	1	1	1	1	0	0	0	1	1	1	62
1034				1	0	0	1	0	1	1	0	0	0	1	1	44
1035				1	0	1	1	1	0	1	1	1	1	0	0	62
1036				1	1	1	1	0	1	0	0	1	1	1	0	61
1037				1	0	0	1	0	0	0	1	0	1	1	0	39
1038				1	0	1	0	1	0	0	0	0	1	0	1	33
1039				0	0	0	1	1	1	1	1	0	1	1	1	68
1040				1	0	1	0	1	1	0	0	1	1	1	1	63
1041				1	0	0	1	1	0	0	0	0	0	1	0	29
1042				0	0	1	1	1	1	1	1	0	0	0	1	53
1043				1	0	1	0	0	1	0	1	1	0	1	1	53
1044				1	0	0	1	1	1	1	0	1	1	0	0	58
1045				0	1	0	0	0	0	1	0	0	1	0	1	31
1046				1	1	0	0	0	0	0	1	0	1	1	0	40
1047				0	0	0	0	0	1	1	1	1	0	0	0	38
1048				0	1	0	1	0	1	1	0	1	1	1	1	66
1049				1	0	0	0	1	0	0	0	1	0	0	0	23
1050				1	0	1	0	1	0	0	1	0	0	1	0	37
1051				0	1	1	0	1	1	0	1	0	0	0	1	45
1052				1	0	0	1	0	0	1	1	1	1	0	1	53
1053				1	1	0	0	1	1	0	1	0	1	0	0	49
1054				1	1	0	1	0	0	1	1	1	0	1	1	60

1055				1	0	0	0	0	0	0	1	0	0	1	1	29
1056				0	1	0	0	0	0	0	1	1	1	0	0	36
1057				0	1	0	1	0	1	0	0	0	1	1	0	42
1058				1	0	0	0	0	0	0	1	1	1	0	0	33
1059				1	0	1	0	0	0	1	0	1	1	0	1	43
1060				1	0	0	1	0	0	0	0	1	0	0	1	25
1061				1	0	1	1	1	1	0	0	0	0	0	0	34
1062				1	0	1	1	0	1	0	1	0	1	0	1	49
1063				1	1	0	0	0	0	0	1	1	0	1	0	40
1064				1	1	0	0	1	0	0	0	1	1	0	0	40
1065				1	1	0	0	0	0	0	0	0	1	1	1	36
1066				1	0	0	1	1	1	0	1	0	0	0	0	38
1067				0	1	0	1	0	0	1	0	1	1	0	0	41
1068				0	1	1	0	0	0	1	1	1	1	1	0	59
1069				0	1	0	0	0	1	0	1	0	1	0	1	41
1070				1	1	1	0	1	0	0	0	0	0	1	0	35
1071				1	0	0	0	1	1	0	1	1	1	0	0	52
1072				0	0	1	1	1	0	0	0	0	0	0	0	20
1073				0	1	1	0	0	1	0	0	1	0	1	0	42
1074				0	0	0	1	1	0	1	1	1	0	1	1	58
1075				1	1	1	0	0	0	0	1	1	1	1	1	60
1076				1	0	0	0	0	0	1	0	0	0	0	1	18
1077				1	1	1	0	0	0	0	0	1	0	1	0	36
1078				1	0	1	1	1	0	0	1	1	0	1	1	58
1079				0	1	0	1	0	1	1	1	1	0	0	0	51
1080				0	0	0	1	0	0	0	0	1	0	1	1	31

QualiPresc - Qualidade da Prescrição na Atenção Primária à Saúde

Instruções
1. Selecione o indicador no Quadro abaixo; e 2. Indique com um X, no Quadro à direita, o mês de início de uma nova mediana.

Indicadores
1. Data de nascimento do paciente
2. Identificação do prescritor
3. Registro de relato de alergia
4. Medicamento padronizado pela instituição
5. Princípio ativo
6. Concentração
7. Dose
8. Forma farmacêutica
9. Via de administração
10. Frequência de administração
11. Duração do tratamento
12. Orientações sobre uso do medicamento
13. Recomendações não farmacológicas
14. Qualidade

8. Forma farmacêutica

Mês	Valor	Mediana	Nova Mediana
jan/21			
fev/21			
mar/21			
abr/21			
mai/21			x
jun/21			
jul/21			
ago/21			
set/21			
out/21			x
nov/21			
dez/21			x
jan/22			
fev/22			
mar/22			
abr/22			
mai/22			
jun/22			
jul/22			
ago/22			
set/22			
out/22			
nov/22			
dez/22			
jan/23			
fev/23			
mar/23			
abr/23			
mai/23			
jun/23			
jul/23			
ago/23			
set/23			
out/23			
nov/23			
dez/23			

Run Chart: 8. Forma farmacêutica

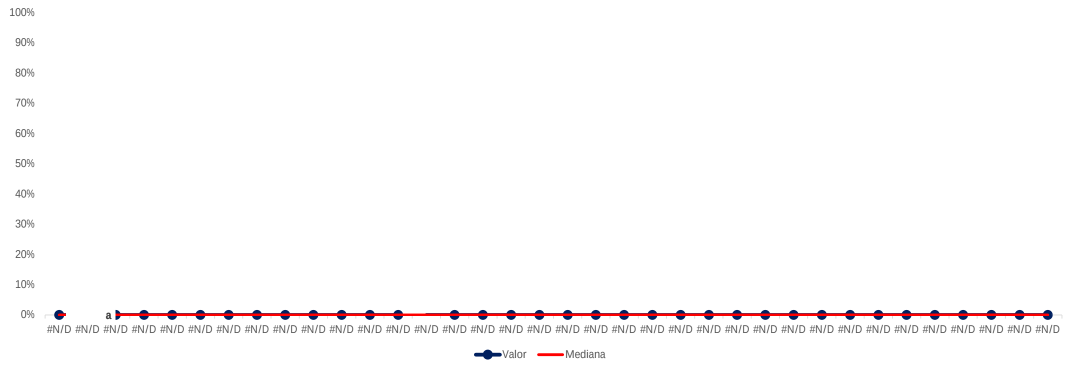

Supplement: S1 File — (PDF) [file pone.0267707.s004.pdf]
