# Supplementary material for: Validation of the QualiPresc instrument for assessing the quality of drug prescription writing in primary health care
Source: PLoS One. 2022 May 11;17(5):e0267707. doi: 10.1371/journal.pone.0267707 (PMC9094502; doi:10.1371/journal.pone.0267707)

QualiPresc - Quality of Prescription in Primary Health Care

Instructions:

The QualiPresc instrument aims to facilitate the monitoring of the quality of drug prescriptions based on writing criteria. Writing criteria are understood to be those related to the structure of the prescription document based on user, prescriber and prescribed drug information.

How to use:

1) The instrument includes 13 indicators of the quality of drug prescriptions that have different weights for the effectiveness and safety of the prescription.
2) QualiPresc should preferably be applied by pharmacists and/or after training given by this professional.
3) Before collecting data, evaluators should carefully read the definitions and clarifications for the measurement of indicators in the table below.
4) After reading, click on the next worksheet called "collect" or on the arrow to the side to record the data of the prescriptions evaluated. ➡

INDICATOR	DEFINITIONS/CLARIFICATIONS FOR THE MEASUREMENT	SCORE[3]		
		S	E	Q
**1. Patient's date of birth**	Day/month/year.	7,1	6,9	6,4
**2. Prescriber's identification**	Covers the professional registration number from the Regional Council, profession, name and surname of the prescriber. The intermediate words to the first name and the last name can be made up of the initial letters.	6,9	4,8	4,2
**3. Record of allergy report**	Record of information about the fact that the user is allergic to a certain drug (s). It must be included in the prescription, regardless of whether the drug involved is present in the prescription.	8,3	6,6	7
**4. Medicine included in the institutional list officially approved[1]**	Component of an institution's standard list of drugs, preferably standardized according to the epidemiological profile and best scientific evidence of efficacy, safety, and cost-effectiveness. Alternatively, the standard list of the essential municipal, state, or national medicines can be used as the basis, instead of the standard list of a specific institution, considering the most appropriate to the context.	6	6,3	4,8
**5.Active ingredient**	This is the Brazilian Common Denomination (DCB) or, in its absence, the International Common Denomination (DCI). Short drug names or chemical	6,9	6,6	5,8
**6. Concentration[2]**	Corresponds to the amount of active ingredient contained in each unit dose. For solid dosage forms, 1 unit dose corresponds to 1 unit dosage form (e.g., 1 tablet). For semi-solid and liquid dosage forms, 1 dose unit corresponds to 1 unit of measure (e.g., 1 mL, 1 g). The acronyms U, u, and UI should not be used, as they can be confused with 0, 4, cc, IV, or 10; an international unit should be written instead. The acronyms mcg and μg should not be	8,3	8,7	9,2
**7. Dosage[2]**	The amount of medication to be administered at each time of use. Comprised of a numerical value and a unit of measure. As a unit of measure, consider the name of the solid pharmaceutical form, drops, or nationally and/or internationally recommended abbreviations/acronyms/symbols. The acronyms U, u, and UI should not be used, as they can be confused with 0, 4, cc, IV, or 10; an international unit should be written instead. The	8,6	9	9,9
**8. Pharmaceutical form**	Final physical form of the drug after mixing active ingredients and excipients during the production process.	8,3	8,4	8,9
**9. Route of administration**	Gateway through which the medication is administered in order to reach its place of action (e.g., oral, intramuscular, intravenous, etc.). Prefer using	8,3	8,7	9,2
**10. Frequency of administration**	Number of times each dose of medication should be administered, considering a 24-hour period (e.g., every 12 hours, etc.).	8,6	9	9,9
**11. Duration of treatment**	Period of time during which the medicine should be used (e.g., for 10 days, continuous use, etc.). If the prescription contains more than 1 medication, [illegible]	8,3	9	9,6
**12. Directions on the use of the drugs[1]**	Directions, such as the best period for medication administration (e.g., morning/night, before/after meals, fasting, after bathing), duration of medication administration (e.g., n minutes, until the contents of the container are complete), thin layer application (e.g., topical use), dilution of the medication, instruction for using an inhalation device, among others, that are necessary for the user's understanding of how to use the medication.	8,6	9	9,9
**13. Non-pharmacological recommendations**	Information regarding non-drug treatment (e.g., diet, physical activity, etc.).	6	6,9	5,3

[1] In indicators 4 to 12, if the prescription has more than 1 drug, this item is considered as compliant only if all the prescription drugs meet the indicator.

[2] Do not use abbreviations U, u and UI, as they can be confused with 0 or 4 or cc or IV or 10, write international unit. Do not use abbreviations mcg or μg, write microgram. Do not use trailing zeros or omit leading zeros, as the decimal place may go unnoticed, write X mg and 0.X mg, respectively. For doses or volumes with fractional numbers (eg 2.5mL), observe in both prescription copies that the comma is well positioned and clear. Do not use a period instead of a comma [4,5].

[3] Weighting scores with respect to Safety (S) and Effectiveness (E), for weighting the composite indicator Quality (Q).

[4] The Joint Commission. Facts about Official Do-Not-Use List. Available in: http://www.jointcommission.org/assets/1/18/Do_Not_Use_List.pdf. Accessed on: April 18, 2021.

[5] Ministério da Saúde. Protocolo de segurança na prescrição, uso e administração de medicamentos. 2013. Available in: https://proqualis.net/sites/proqualis.net/files/000002490IQmwD8.pdf.

QualiPresc - Quality of Prescription in Primary Health Care

Data collect:

1. To ensure continuous monitoring, select a minimum of 20 to 30 prescriptions. Make a random selection (draw among all prescriptions of the evaluated period), to ensure representativeness.
2. Include only legible prescriptions and place the identification number of those included in the first column after the collection date. If any are illegible, draw another prescription from the same period to complete the sample.
3. For the included prescriptions, respond with 0 or 1 if the indicators are compliant or non-compliant, respectively.
4. After entering the answers, check the consolidated results of the 13 simple indicators and the 3 composite indicators of QualiPresc in the following spreadsheet.

IDENTIFICATION			INDICADORES SIMPLES													
ID	Date	Prescription no.	1. Patient's date of birth	2. Prescriber's identification	3. Record of allergy report	4. Medicine included in the institutional list officially approved	5.Active ingredient	6. Concentration	7. Dosage	8. Pharmaceutical form	9. Route of administration	10. Frequency of administration	11. Duration of treatment	12. Directions on the use of the drugs	13. Non-pharmacological recommendations	14. QUALITY
1			1	0	1	0	1	0	1	0	1	1	0	1	1	63
2			0	0	1	0	0	1	0	1	1	0	0	0	1	40
3			0	0	1	1	1	0	0	1	1	1	0	0	0	46
4			0	0	1	1	1	1	1	0	1	1	1	1	0	75
5			1	0	1	0	0	1	0	0	0	0	1	1	0	42
6			0	0	1	0	0	1	1	1	0	0	1	0	0	45
7			1	0	0	0	1	1	1	0	0	1	1	0	1	56
8			1	1	0	0	0	0	0	1	1	0	0	1	0	39
9			1	1	0	1	1	1	0	1	1	0	0	1	1	64
10			0	0	1	0	1	1	0	0	0	1	1	1	1	57
11			1	0	1	1	1	0	1	0	0	0	1	1	1	59
12			0	1	1	1	0	1	0	0	1	0	0	1	1	50
13			0	1	0	1	1	0	1	1	1	1	0	0	1	58
14			1	1	1	0	0	0	1	1	0	1	1	0	0	56
15			1	1	0	0	1	1	0	0	0	1	0	0	1	41
16			1	1	1	0	0	0	0	1	0	1	1	0	1	51
17			1	1	0	0	1	1	0	1	0	1	0	1	0	54
18			1	0	0	0	0	0	1	1	1	0	0	1	0	44
19			1	1	0	1	1	0	1	0	0	1	1	1	0	61
20			0	1	0	1	0	0	0	1	1	1	0	0	1	42
21			0	1	0	1	0	1	1	1	1	0	0	0	0	46
22			1	0	0	0	0	1	1	0	1	1	1	0	0	54
23			1	1	0	0	1	0	0	0	0	1	1	0	1	41
24			1	0	0	0	1	1	0	0	1	0	0	0	0	31
25			0	1	1	0	0	1	0	0	0	0	1	1	0	40
26			0	0	0	0	1	1	0	0	1	0	1	1	1	49
27			1	1	0	0	1	0	0	1	1	0	0	1	0	44
28			1	1	1	1	0	1	0	0	1	0	0	0	0	41
29			0	1	1	0	1	0	0	1	1	1	1	0	1	60
30			0	1	1	0	0	1	1	1	1	1	1	1	1	83
31			0	1	1	1	1	0	1	0	1	0	0	0	0	41
32			0	0	1	1	1	0	0	1	1	0	0	1	0	46
33			0	1	0	0	1	1	1	0	0	1	0	1	1	54
34			0	1	1	1	1	0	1	1	0	0	1	0	1	56
35			1	0	0	1	1	1	1	0	1	1	0	1	1	70
36			1	0	0	0	1	1	0	1	0	1	0	0	1	46
37			0	1	0	0	1	1	0	1	0	1	1	1	0	58
38			1	1	1	1	0	0	0	0	0	0	0	1	1	38
39			1	0	1	1	0	1	0	1	0	0	1	0	1	51
40			1	1	0	0	1	1	1	1	0	0	0	1	0	54
41			0	0	0	0	0	1	1	1	0	1	1	1	0	57
42			1	1	0	0	0	1	0	0	1	0	0	0	1	34
43			1	0	0	1	0	1	1	0	1	0	1	0	0	49
44			1	1	1	0	1	0	1	1	1	1	0	0	1	67
45			1	0	0	1	1	1	1	1	0	0	1	1	1	70
46			1	1	0	0	0	1	0	1	1	0	0	1	0	48
47			0	1	1	0	1	0	0	1	0	0	1	0	1	41
48			0	1	1	1	1	0	0	0	1	0	0	0	0	31
49			1	0	1	0	1	0	1	1	1	1	1	1	1	82
50			0	1	1	1	1	1	0	0	0	1	1	0	0	51
51			1	1	0	1	1	0	1	0	1	0	1	0	0	50
52			1	1	0	0	1	1	1	1	1	0	1	0	1	69
53			0	1	0	1	1	0	1	1	1	0	0	0	0	43
54			1	1	0	1	1	0	1	0	0	0	0	0	0	31
55			1	1	1	1	0	1	1	0	1	1	1	0	0	70
56			1	1	1	0	1	0	1	1	0	1	1	0	1	67
57			1	0	0	1	1	1	0	0	1	0	0	0	1	41
58			0	1	0	0	1	0	0	1	1	0	1	0	0	38
59			1	0	1	1	0	1	0	1	1	1	1	0	1	70
60			1	1	1	1	1	0	0	1	0	0	0	0	0	37
61			1	0	1	1	1	0	1	1	1	1	0	0	1	67
62			1	0	1	0	1	0	0	0	1	0	1	0	0	38
63			0	0	0	1	1	1	0	0	1	0	0	0	1	34
64			1	1	1	1	1	1	1	0	0	0	1	1	0	67
65			0	0	1	0	1	0	0	1	1	1	0	1	1	56
66			1	1	1	1	0	0	0	1	0	0	1	1	0	51
67			0	0	0	0	0	0	0	1	0	0	1	0	0	19
68			1	0	1	1	0	0	0	1	1	0	1	1	0	56
69			1	0	1	0	1	1	0	1	0	0	0	1	0	47
70			0	0	1	0	0	0	0	0	0	1	1	0	1	32
71			0	1	0	1	1	1	0	1	0	1	1	1	1	68
72			1	1	1	1	0	0	0	0	0	1	0	1	1	48
73			0	1	0	0	1	1	0	1	1	0	1	0	1	52
74			1	1	1	1	0	1	0	1	1	0	1	0	1	65
75			1	1	1	0	0	0	1	0	1	0	1	0	0	46
76			1	0	1	0	0	1	1	0	0	1	1	0	0	52
77			0	1	0	0	1	1	1	0	1	1	0	0	0	48
78			0	1	1	0	1	0	1	0	1	0	1	1	0	56
79			1	0	1	0	1	1	1	1	1	0	0	1	1	72
80			1	1	1	1	1	1	1	0	1	0	0	1	0	66
81			1	0	1	1	0	0	1	0	0	0	1	0	1	43
82			1	0	1	0	1	1	0	1	1	0	0	1	0	56
83			1	0	1	1	1	1	0	1	0	1	0	0	0	52
84			1	1	0	0	0	0	0	1	1	1	1	1	0	58
85			1	1	0	1	0	1	0	0	1	1	0	1	0	54
86			1	0	0	1	1	0	1	0	1	0	0	1	0	46
87			1	1	0	0	0	1	1	1	0	0	1	0	1	54
88			1	0	1	0	1	0	1	1	0	0	1	1	0	58
89			0	1	1	0	1	0	1	1	1	0	1	0	0	55
90			0	0	0	1	0	0	1	0	1	1	0	1	1	49
91			1	1	1	0	0	1	0	0	0	0	1	0	1	42
92			1	1	0	1	1	0	0	0	1	1	1	0	0	50
93			1	1	1	1	0	1	0	0	0	0	0	1	0	42
94			1	1	1	0	0	1	1	1	0	0	1	1	0	65
95			1	1	1	0	1	1	0	1	1	0	1	1	0	70
96			0	0	1	1	1	1	1	1	1	1	1	0	1	80
97			0	1	1	0	1	1	0	0	0	1	0	1	1	51
98			1	1	0	1	1	1	1	0	1	1	0	1	0	69
99			0	0	0	1	0	0	1	0	0	0	1	1	0	34
100			1	1	1	0	0	1	0	0	1	0	0	0	1	41
101			0	1	1	1	0	0	0	0	1	0	0	1	0	35
102			1	1	0	0	1	0	1	1	0	1	1	1	0	65
103			1	0	1	0	1	0	0	1	1	0	0	0	0	37
104			1	0	1	1	1	1	0	0	0	1	1	1	1	68
105			0	0	0	0	0	0	1	0	1	1	0	0	1	34
106			0	0	0	1	1	1	1	0	1	1	1	0	0	58
107			1	1	0	1	1	1	1	1	0	0	0	0	0	49

Answer options
0
1

108			0	0	1	1	1	1	1	0	1	1	1	0	1	71
109			1	0	0	1	0	0	0	0	1	1	1	1	0	50
110			1	1	1	1	1	0	0	0	0	1	1	0	0	48
111			1	0	0	1	1	1	0	0	0	1	1	1	0	56
112			0	1	0	1	1	0	1	1	1	1	1	0	1	68
113			1	1	0	0	1	0	0	0	1	1	0	0	1	41
114			1	0	1	1	1	1	1	1	1	0	0	0	1	67
115			1	1	1	1	0	1	0	1	1	1	0	0	1	65
116			1	1	0	0	1	0	0	1	1	0	0	1	1	50
117			1	1	1	1	1	0	1	0	1	1	1	1	0	77
118			1	1	1	1	0	1	0	1	0	1	1	0	0	60
119			0	1	0	0	1	0	1	0	1	0	0	0	0	29
120			1	0	1	0	0	1	0	0	1	0	1	1	0	51
121			0	0	1	1	1	0	1	1	1	0	1	1	0	65
122			0	1	1	1	1	0	0	0	0	0	1	1	0	41
123			1	0	0	1	1	0	0	0	1	1	1	0	0	46
124			0	1	1	0	1	1	1	1	0	1	0	1	0	65
125			1	1	0	1	1	1	0	1	1	1	0	1	1	74
126			0	1	1	1	0	0	0	0	1	1	0	1	0	45
127			0	0	1	1	0	0	0	0	1	1	0	1	1	46
128			1	0	1	0	0	1	1	1	0	1	1	0	1	66
129			0	1	1	0	1	1	1	0	0	0	1	0	0	46
130			1	1	0	0	0	0	1	0	0	1	1	0	0	40
131			1	1	1	1	1	1	0	0	0	1	0	1	0	57
132			1	0	1	0	0	1	0	1	1	1	0	0	1	56
133			1	0	0	0	0	1	1	1	1	1	0	0	0	54
134			0	1	0	1	0	0	0	1	0	0	0	1	1	33
135			0	0	1	1	1	1	0	0	1	1	0	1	0	56
136			0	1	0	0	1	0	1	1	1	0	1	1	1	63
137			1	1	1	0	0	1	0	0	0	1	1	1	1	62
138			0	1	1	0	0	1	0	0	0	1	0	0	0	30
139			0	1	0	1	0	0	0	0	0	1	0	1	0	29
140			0	0	1	0	1	1	0	1	1	0	0	1	0	50
141			1	1	1	0	0	0	0	1	0	0	1	0	0	36
142			0	0	0	0	1	1	0	1	0	1	0	1	1	49
143			0	0	1	0	0	0	0	1	1	1	1	0	1	50
144			0	0	0	1	0	1	0	1	1	0	0	1	1	47
145			1	1	0	1	1	1	0	1	1	1	0	1	1	74
146			0	1	0	0	1	1	0	0	1	1	0	1	1	54
147			1	0	0	1	0	0	1	1	0	1	0	1	0	50
148			0	0	0	0	0	0	1	1	0	0	1	0	0	28
149			1	0	0	1	1	1	0	0	1	1	1	1	0	65
150			0	0	0	1	0	0	1	1	1	1	1	1	0	62
151			1	0	1	0	0	0	0	0	1	1	1	0	1	47
152			1	1	0	1	0	1	0	1	0	1	0	1	1	59
153			1	0	1	1	1	0	1	1	1	0	0	1	0	62
154			0	1	1	0	1	0	1	1	1	0	1	1	0	65
155			0	1	0	0	0	0	1	1	0	0	0	0	0	23
156			0	0	1	0	1	1	1	0	1	1	0	1	1	66
157			0	0	0	1	0	0	0	0	1	0	1	0	1	29
158			0	1	0	1	0	1	0	1	0	1	0	1	0	47
159			1	0	1	1	0	1	0	1	1	1	0	0	0	55
160			0	1	0	1	0	1	0	0	0	1	1	1	0	48
161			1	0	0	0	0	0	1	1	0	1	1	1	1	60
162			0	1	0	1	1	0	1	1	0	0	0	0	1	39
163			1	1	0	1	0	0	1	1	1	0	1	0	1	58
164			1	1	1	1	1	1	0	1	0	0	1	1	1	71
165			0	0	0	0	0	0	1	0	1	1	1	1	0	49
166			0	1	0	0	0	0	0	0	0	0	0	0	1	10
167			0	1	1	0	0	0	1	1	0	1	0	0	0	40
168			0	0	1	0	1	1	1	1	1	0	0	0	1	55
169			0	1	0	0	1	0	1	1	0	0	0	0	0	29
170			1	0	0	0	1	1	0	0	1	1	0	1	0	50
171			1	0	1	0	0	0	0	0	0	1	1	1	1	48
172			1	1	1	0	0	1	0	1	0	1	0	0	0	46
173			1	1	0	0	1	0	1	0	1	1	0	0	1	51
174			1	1	0	0	0	1	0	1	0	1	1	1	0	58
175			1	0	1	1	0	1	0	0	0	0	1	1	0	47
176			0	0	1	1	1	0	0	0	0	0	1	0	1	33
177			1	0	0	0	0	1	0	1	1	1	1	1	0	63
178			1	1	0	1	0	1	1	1	1	1	0	1	0	72
179			1	1	0	0	0	1	0	0	0	0	1	1	0	39
180			0	1	1	1	1	1	1	1	1	1	1	1	1	94
181			1	0	0	1	1	0	1	0	0	1	0	0	0	37
182			1	1	0	0	0	0	1	1	1	0	0	0	0	39
183			0	1	0	1	1	1	1	1	0	0	1	0	1	58
184			0	0	1	0	0	0	0	0	0	1	0	0	0	17
185			1	0	0	1	0	1	1	0	1	1	1	1	0	69
186			0	0	0	0	0	0	1	1	0	1	1	1	1	54
187			1	1	0	1	0	1	0	0	1	1	0	0	0	44
188			1	0	1	1	1	0	0	1	0	1	1	0	0	52
189			1	1	0	1	1	1	1	1	0	1	1	1	1	84
190			1	0	0	1	1	0	0	0	1	1	0	0	1	41
191			0	1	0	1	0	1	0	1	1	0	0	0	0	36
192			0	0	1	0	1	0	1	0	1	1	0	1	0	52
193			1	0	0	0	0	0	1	1	0	1	1	0	0	45
194			0	1	0	0	0	0	1	1	0	1	0	0	0	33
195			1	1	0	0	1	1	0	1	1	1	0	1	0	64
196			0	0	0	0	0	0	1	1	0	0	0	0	0	19
197			0	1	1	0	0	0	0	0	1	1	1	1	0	50
198			0	1	1	0	0	1	0	0	0	0	0	0	0	20
199			1	1	1	1	1	1	1	1	1	1	0	1	0	85
200			0	0	1	1	1	1	0	1	0	0	1	1	0	55
201			1	0	0	0	1	1	1	0	1	0	0	0	1	46
202			1	1	1	1	0	0	1	0	1	0	0	1	0	51
203			0	0	1	1	1	1	1	1	0	1	1	1	0	75
204			1	0	1	0	0	1	0	0	1	0	1	0	1	47
205			0	0	0	0	0	1	1	0	1	0	1	1	0	48
206			1	0	0	0	0	1	1	1	1	1	0	0	1	59
207			1	0	0	1	1	1	1	1	1	1	0	1	0	74
208			1	1	0	0	0	1	1	1	1	1	0	1	0	68
209			0	1	1	1	1	1	1	1	0	0	1	0	0	59
210			0	1	1	1	0	0	1	1	0	1	1	1	0	64
211			1	1	1	1	1	0	0	0	0	1	0	1	1	53
212			0	1	1	1	1	0	0	0	0	0	1	1	1	47
213			1	1	0	0	1	1	1	0	1	0	0	0	1	50
214			1	0	0	0	1	1	0	1	1	1	0	0	1	55
215			0	0	0	0	1	1	0	0	0	0	0	0	0	15
216			0	1	1	1	1	1	0	1	0	1	1	1	0	69
217			0	0	0	1	0	1	0	0	1	1	1	1	1	58
218			1	0	1	0	1	1	1	0	0	1	0	0	1	54
219			1	0	0	0	1	1	0	1	0	0	1	0	0	40
220			1	1	0	0	0	0	0	0	1	1	0	0	0	30
221			1	0	0	1	1	1	0	1	0	1	0	0	1	50
222			0	0	0	0	1	1	1	1	0	1	1	0	1	59
223			1	1	0	0	0	1	0	0	0	1	1	0	0	39
224			1	1	0	1	0	1	1	1	1	1	0	1	1	78
225			0	1	0	0	1	1	1	1	1	1	1	1	0	77

226			1	0	1	1	0	1	1	0	1	1	1	0	0	66
227			1	1	0	0	0	1	1	1	0	0	1	1	0	58
228			1	1	0	1	0	1	0	0	1	0	0	1	1	49
229			0	0	0	0	1	0	0	0	1	0	1	1	1	40
230			1	1	0	1	1	1	1	0	0	1	1	0	0	60
231			0	0	1	1	0	0	0	1	1	1	1	0	1	55
232			1	1	1	1	1	0	1	1	0	0	0	1	1	62
233			0	0	0	1	0	0	1	0	1	0	0	0	0	24
234			1	1	0	1	0	1	0	1	1	0	0	1	1	58
235			0	0	1	0	1	1	1	1	1	1	1	1	0	79
236			1	1	0	1	1	1	0	0	0	1	0	0	0	40
237			1	0	1	1	1	0	0	1	0	0	0	0	0	33
238			0	0	1	0	1	1	0	1	0	0	1	1	0	50
239			1	1	0	1	1	0	0	1	1	1	0	1	0	59
240			1	1	0	0	0	0	1	1	1	0	0	0	1	44
241			1	1	1	0	1	1	0	0	0	1	0	0	0	43
242			0	0	0	0	0	1	0	1	1	1	1	0	0	47
243			0	0	0	1	1	1	1	0	1	1	0	1	1	64
244			0	0	0	0	0	1	0	1	1	0	0	1	1	43
245			0	1	0	1	0	1	1	1	1	1	1	1	1	81
246			1	0	0	1	0	1	1	1	0	1	0	1	1	64
247			1	1	0	1	0	1	1	1	0	1	0	1	1	69
248			0	0	0	1	0	1	1	0	0	0	0	0	0	24
249			0	0	0	0	0	0	0	1	1	0	0	1	1	33
250			0	0	1	1	0	0	0	0	0	1	0	1	0	32
251			1	1	0	1	0	1	1	0	0	0	1	1	0	54
252			1	1	1	0	0	0	0	0	0	1	1	1	0	47
253			1	0	1	0	1	0	1	0	1	0	0	0	1	44
254			0	1	1	0	0	1	1	1	0	1	1	0	1	64
255			1	1	0	0	1	1	1	0	1	0	0	1	0	55
256			0	1	1	1	1	0	0	1	0	1	0	0	1	46
257			0	0	0	0	0	1	1	1	0	1	0	0	0	38
258			0	0	1	1	1	0	1	0	1	0	0	1	1	52
259			1	1	0	0	0	1	0	0	0	0	1	0	0	29
260			1	1	0	1	1	0	0	0	0	0	1	0	1	36
261			1	1	0	1	0	1	1	0	0	0	0	1	0	44
262			0	0	1	0	1	0	1	1	0	1	0	1	1	57
263			0	0	0	0	0	0	0	1	1	0	0	0	0	18
264			1	1	1	1	0	1	1	0	1	1	0	0	0	61
265			0	1	0	1	0	1	0	0	0	1	1	1	1	53
266			1	1	0	0	1	1	0	0	1	1	0	1	0	55
267			0	0	1	1	1	1	1	0	0	1	1	1	1	71
268			1	1	0	1	1	0	1	0	1	1	1	0	1	65
269			1	1	1	1	0	1	0	0	1	0	1	1	1	66
270			1	1	1	1	0	1	0	1	1	0	1	0	1	65
271			1	1	0	0	0	1	0	0	0	0	0	0	1	25
272			0	0	0	1	0	1	0	1	1	1	0	0	0	42
273			0	0	0	1	1	1	1	0	0	0	1	1	0	49
274			0	0	1	0	1	0	0	0	0	1	0	1	0	33
275			1	1	0	1	1	0	1	0	1	1	1	0	0	60
276			1	1	0	0	0	1	0	1	0	1	0	1	1	54
277			1	1	1	0	1	0	1	0	1	1	0	0	1	58
278			0	1	1	0	0	1	0	1	0	0	0	0	1	35
279			0	0	0	0	1	1	1	0	0	0	0	1	0	35
280			0	1	1	0	0	0	0	0	0	0	0	0	0	11
281			0	0	0	0	1	1	1	1	0	1	1	0	1	59
282			1	0	0	0	0	1	1	1	0	1	0	0	0	44
283			1	0	0	0	0	1	1	0	0	0	0	1	0	35
284			1	0	0	1	0	1	1	1	0	1	0	1	1	64
285			1	0	1	1	1	0	1	0	0	0	1	1	1	59
286			0	0	1	1	0	1	1	0	1	0	0	1	1	55
287			1	0	0	1	1	1	1	0	1	1	0	0	0	55
288			0	0	0	0	0	0	0	1	1	0	1	0	1	33
289			0	1	0	1	0	1	1	1	0	1	0	1	0	57
290			0	0	0	1	1	0	1	0	1	0	0	0	1	35
291			0	1	1	1	0	0	0	1	0	0	0	0	0	25
292			1	0	0	0	0	1	0	1	1	1	0	1	0	54
293			0	0	1	1	1	0	1	1	0	0	1	1	0	56
294			1	0	1	0	0	0	0	0	1	0	1	0	0	32
295			0	1	1	1	0	1	1	1	1	1	0	0	1	68
296			1	1	1	0	0	0	0	0	0	0	0	1	1	33
297			1	1	1	0	0	1	1	0	1	0	0	0	0	46
298			1	0	0	0	1	0	0	1	1	1	1	1	1	65
299			1	0	0	1	1	1	0	0	0	1	1	0	1	51
300			1	0	1	0	1	0	1	0	0	0	1	1	1	54
301			0	0	1	0	1	1	0	1	0	0	1	0	0	41
302			0	0	0	1	1	1	1	1	0	0	1	0	0	48
303			1	0	0	0	1	1	0	1	1	0	0	0	1	45
304			1	0	1	1	1	0	1	1	0	0	1	0	1	58
305			0	0	0	0	1	1	1	0	1	1	0	0	0	44
306			0	0	0	1	0	0	1	0	0	1	1	0	0	34
307			0	1	0	1	0	1	1	1	0	0	0	0	0	37
308			1	0	1	1	0	0	0	0	0	1	0	0	0	28
309			0	0	0	0	0	0	0	0	0	0	0	0	0	0
310			0	0	1	1	1	0	0	0	1	0	0	1	0	37
311			0	0	0	1	1	1	1	0	1	1	1	0	0	58
312			1	1	1	0	0	1	1	1	1	1	1	1	1	90
313			0	0	0	0	1	0	0	1	0	1	1	1	0	44
314			0	1	1	0	1	0	1	1	0	1	1	1	1	71
315			0	0	1	0	1	1	0	1	1	1	0	0	0	50
316			1	1	1	1	0	1	1	0	1	1	0	1	0	71
317			0	1	0	1	0	1	0	0	0	1	0	0	1	33
318			1	1	0	0	1	1	1	1	0	0	1	1	1	69
319			0	0	0	1	0	0	0	1	0	1	1	0	1	39
320			0	1	0	1	1	0	1	1	1	1	0	1	1	68
321			1	1	0	0	0	0	1	1	0	1	0	0	1	45
322			1	1	1	1	0	0	0	1	1	1	1	1	0	70
323			1	1	1	0	1	1	0	0	0	0	0	0	0	33
324			0	1	0	1	0	1	0	1	1	1	0	0	0	46
325			1	0	1	0	1	0	0	1	1	1	0	1	0	57
326			1	0	0	1	1	1	0	1	0	1	1	0	1	60
327			1	1	1	0	0	0	1	0	1	0	0	1	1	52
328			1	0	1	0	0	1	0	0	0	0	1	1	1	47
329			1	0	0	1	1	0	1	0	1	0	1	1	0	56
330			1	1	0	1	0	1	0	1	1	0	0	0	0	43
331			0	1	0	1	1	0	0	0	1	0	1	1	0	44
332			0	1	1	0	1	1	0	0	0	1	0	0	0	36
333			0	0	0	1	1	0	1	0	1	0	1	0	1	45
334			0	0	0	1	0	1	1	1	1	0	1	1	0	62
335			0	0	0	0	1	1	0	0	1	0	0	1	1	39
336			0	1	1	0	1	0	1	1	1	0	1	1	0	65
337			0	1	0	0	0	0	1	0	1	0	1	1	0	43
338			1	0	0	0	1	0	0	1	0	0	0	1	0	31
339			1	1	1	0	0	0	0	0	1	0	1	1	1	52
340			1	0	1	0	1	1	1	0	1	1	0	1	1	73
341			0	1	1	1	0	1	1	1	0	0	1	0	0	54
342			0	1	1	0	0	1	0	0	0	0	0	1	1	36
343			0	0	1	1	1	0	0	0	0	1	1	0	1	42

344			0	1	0	0	0	0	1	1	1	0	0	1	1	47
345			0	1	0	0	1	0	0	0	1	0	0	1	0	29
346			0	0	0	0	0	1	1	1	0	0	1	1	1	53
347			0	0	0	1	1	0	0	0	1	1	1	0	1	45
348			0	0	0	0	1	1	0	0	1	1	1	0	0	44
349			0	1	1	0	0	1	0	1	1	1	1	0	1	63
350			1	0	0	1	0	0	1	1	1	0	1	0	0	49
351			1	0	1	0	1	0	1	0	0	1	0	0	0	39
352			1	0	0	0	0	0	1	1	0	0	0	1	0	35
353			0	0	0	0	0	1	1	0	0	0	1	0	1	34
354			1	0	1	1	0	1	1	1	0	0	0	0	0	46
355			1	0	0	1	0	0	1	0	1	1	1	1	0	60
356			1	1	1	1	1	0	0	0	0	0	1	0	1	43
357			0	1	1	1	1	1	0	1	0	1	0	0	1	55
358			0	1	0	1	0	1	0	1	0	1	0	1	0	47
359			0	1	0	1	1	0	1	1	1	0	1	0	1	58
360			0	0	0	1	0	0	0	1	0	1	1	0	1	39
361			1	1	0	1	1	0	0	0	0	1	0	1	0	41
362			1	1	1	1	1	0	0	1	0	0	1	1	0	57
363			1	1	1	0	1	0	1	0	0	0	0	1	0	43
364			1	0	0	0	0	1	1	0	0	1	0	0	0	35
365			1	1	0	1	1	1	0	1	1	0	1	0	1	63
366			1	0	0	1	1	1	1	1	1	1	1	1	0	84
367			1	0	1	0	1	1	1	1	0	0	0	0	0	47
368			1	1	0	0	1	0	0	0	1	1	1	1	0	55
369			1	1	0	0	0	1	0	0	0	0	0	0	1	25
370			1	0	1	0	0	1	1	0	1	0	0	0	1	47
371				1	1	1	1	0	1	0	1	1	1	1	0	70
372				1	0	1	1	1	0	0	1	0	1	1	0	53
373				0	0	1	1	0	1	0	0	1	1	1	0	50
374				1	0	1	1	1	0	0	0	0	1	1	1	49
375				1	0	0	0	0	0	1	0	1	0	1	1	38
376				0	1	0	1	0	0	1	0	0	1	0	1	37
377				0	1	0	0	0	1	1	1	0	1	0	0	45
378				0	0	1	0	0	0	1	1	0	0	1	0	33
379				1	1	1	1	1	1	0	0	1	0	1	1	66
380				1	0	0	1	0	1	1	1	1	1	0	0	58
381				0	1	1	1	1	1	1	0	1	0	0	1	61
382				0	0	0	1	0	1	0	0	1	1	1	1	50
383				1	1	0	1	0	1	1	0	1	1	1	0	65
384				0	1	1	1	0	1	0	1	1	1	1	0	66
385				0	0	0	1	0	0	0	1	0	0	0	0	15
386				0	0	1	1	0	0	1	1	1	0	1	0	49
387				0	1	1	1	0	1	1	0	0	1	1	0	56
388				0	0	0	0	0	0	1	0	1	1	1	1	44
389				1	0	0	1	1	0	1	0	0	1	1	0	48
390				0	1	1	0	1	0	0	1	1	0	1	1	55
391				1	1	0	0	1	1	0	0	1	0	0	1	46
392				1	1	0	1	1	0	0	1	1	0	1	1	61
393				1	1	0	1	0	1	0	0	0	0	0	1	32
394				0	1	1	1	1	0	0	1	1	0	1	0	56
395				0	1	0	1	1	1	1	0	0	1	1	1	66
396				0	0	0	1	1	0	0	0	0	1	1	0	35
397				1	0	0	0	0	0	1	1	0	1	1	0	42
398				0	0	0	1	0	0	1	0	0	0	1	1	30
399				0	0	0	0	1	0	0	1	0	0	1	0	28
400				1	1	1	1	0	1	1	0	0	0	1	0	51
401				0	1	1	1	1	1	0	0	0	1	0	0	46
402				0	1	1	1	1	1	0	0	1	0	0	1	52
403				0	0	1	0	0	0	0	1	1	1	1	1	49
404				0	1	1	1	1	1	0	1	1	0	0	0	56
405				0	0	1	1	1	0	0	0	0	0	1	1	35
406				1	1	0	1	0	0	1	0	1	0	0	0	36
407				1	1	1	0	1	1	1	1	0	0	1	1	68
408				1	1	0	1	0	0	1	1	1	1	1	1	70
409				1	1	0	1	0	0	0	1	0	1	1	1	51
410				1	0	1	0	0	0	0	1	0	0	1	0	28
411				0	0	1	0	1	0	0	1	0	1	0	0	33
412				0	0	1	1	0	1	1	1	1	0	1	1	64
413				0	0	0	0	1	1	0	0	0	0	0	0	19
414				0	1	1	0	0	1	1	1	1	1	0	0	59
415				0	1	1	1	1	0	1	0	0	1	1	1	61
416				0	1	1	0	1	0	1	0	1	0	1	0	50
417				1	0	1	1	1	1	0	1	1	1	1	1	78
418				1	1	0	0	1	1	0	1	0	0	0	1	45
419				1	0	1	0	0	0	1	1	1	1	0	0	47
420				0	1	1	0	0	1	0	1	1	1	0	1	56
421				0	1	0	1	1	0	1	1	0	1	0	0	50
422				1	0	0	1	1	1	0	1	1	1	1	1	73
423				1	0	1	0	1	1	1	1	1	1	0	1	71
424				1	0	1	1	0	1	1	1	1	0	1	0	63
425				0	0	1	1	0	0	1	1	0	1	1	1	54
426				0	0	0	1	0	0	1	0	0	1	1	1	40
427				0	1	0	0	0	0	0	1	1	1	0	0	36
428				0	1	1	0	0	0	0	0	0	1	1	1	37
429				1	1	0	0	0	1	0	0	0	0	0	1	26
430				0	0	0	0	0	0	0	0	0	0	0	1	5
431				1	0	0	1	0	0	1	1	0	1	1	0	48
432				1	0	0	0	1	1	1	1	0	1	0	1	56
433				1	1	1	1	1	1	1	1	1	1	1	1	94
434				1	1	0	0	0	1	0	0	0	0	1	1	36
435				1	1	1	0	0	1	1	0	1	1	0	1	60
436				1	0	0	0	1	1	0	0	0	1	0	1	38
437				1	1	1	0	1	0	0	0	1	0	0	1	40
438				1	1	0	0	0	0	1	1	1	0	0	1	45
439				1	1	0	1	1	1	0	0	1	0	0	0	46
440				1	1	1	0	0	1	0	0	1	1	1	0	55
441				0	1	0	1	1	1	1	1	0	0	1	0	60
442				1	1	0	1	1	0	1	0	1	0	0	1	50
443				1	0	1	0	1	0	0	0	1	1	0	0	38
444				0	0	1	1	1	0	0	1	0	0	0	0	29
445				1	0	1	0	0	0	1	1	1	0	0	1	42
446				0	1	0	0	0	1	1	0	1	0	1	0	46
447				1	0	1	1	1	0	0	1	1	0	1	1	58
448				0	1	1	0	1	1	1	1	1	1	0	0	69
449				1	0	0	0	0	1	0	0	0	0	1	0	24
450				0	0	1	0	1	0	1	0	1	0	0	0	33
451				1	1	1	1	1	0	0	0	0	0	0	1	36
452				1	0	0	1	0	0	1	0	1	1	0	1	44
453				0	0	1	0	1	0	1	0	0	1	1	1	48
454				0	0	0	1	1	0	0	0	0	0	1	0	25
455				0	1	0	0	0	0	0	1	0	1	1	0	36
456				0	0	0	1	0	1	1	1	0	0	1	1	49
457				1	0	1	0	1	1	0	1	0	0	1	1	53
458				1	0	0	0	0	0	1	0	0	1	1	1	38
459				0	1	1	1	0	0	0	1	1	0	1	0	47
460				1	1	1	1	1	1	0	0	1	0	0	0	51
461				0	0	1	0	0	1	1	1	0	1	1	1	58

462				0	1	1	1	0	1	0	1	0	1	0	0	46
463				0	1	0	1	1	1	0	0	0	0	0	1	37
464				1	0	0	1	1	1	0	0	1	0	0	0	39
465				0	0	1	0	0	1	1	1	1	1	0	1	58
466				0	0	1	0	0	1	1	0	1	1	1	1	58
467				0	0	1	1	1	1	1	0	0	1	1	0	58
468				1	1	1	1	1	1	1	1	1	1	1	0	88
469				1	1	0	0	0	1	1	0	1	0	1	0	50
470				0	1	1	0	0	0	0	1	1	1	0	0	41
471				0	1	1	0	0	1	0	0	1	0	0	0	32
472				0	1	0	0	1	0	0	0	1	1	0	1	41
473				0	1	0	1	1	1	1	1	1	1	1	1	85
474				1	1	1	1	1	0	0	1	0	1	0	1	55
475				0	1	1	1	0	1	0	1	1	0	0	1	52
476				1	1	0	0	1	0	0	1	0	1	1	1	54
477				0	0	0	1	1	0	0	1	0	1	0	1	39
478				1	1	0	1	0	1	0	1	1	1	1	0	66
479				1	0	0	0	0	0	0	1	0	0	1	0	23
480				1	0	0	0	1	0	1	1	1	1	0	0	51
481				0	1	0	0	0	1	1	1	1	0	1	1	60
482				0	0	0	0	1	1	1	1	1	1	0	1	62
483				1	1	1	0	0	0	1	0	0	1	1	0	44
484				1	0	0	0	0	0	1	0	1	1	1	1	48
485				0	1	1	1	1	1	0	0	0	0	0	0	37
486				1	0	1	1	0	0	0	0	1	0	0	1	30
487				0	0	1	1	1	0	0	0	1	0	1	1	45
488				1	1	1	1	0	1	0	0	0	1	0	0	41
489				1	1	1	0	1	1	1	0	1	0	1	1	69
490				0	1	0	1	1	0	0	0	1	1	0	0	42
491				1	0	0	0	1	0	0	1	0	0	0	0	23
492				1	1	0	0	0	0	1	0	0	0	0	1	25
493				1	0	0	1	0	1	0	0	0	0	1	0	30
494				1	0	0	0	1	1	0	1	1	0	0	1	48
495				1	1	1	0	0	0	0	0	0	1	0	0	26
496				0	0	1	0	1	0	1	1	0	1	0	1	47
497				1	0	1	0	0	1	0	0	0	1	1	1	44
498				0	1	1	1	1	0	1	1	1	1	1	0	74
499				0	0	0	1	0	1	0	0	1	1	1	1	50
500				0	0	1	1	0	1	0	1	0	0	0	1	35
501				0	1	0	1	0	0	1	0	0	0	1	1	37
502				0	1	0	1	0	1	0	0	1	0	0	0	33
503				0	1	1	0	0	0	0	0	0	1	1	0	31
504				0	1	0	1	1	1	0	0	1	0	1	1	57
505				1	0	0	0	1	1	0	0	0	1	0	0	33
506				1	0	1	0	1	1	0	1	0	1	1	1	62
507				0	1	1	0	0	1	0	1	0	0	1	0	41
508				0	1	1	1	0	1	1	0	1	1	0	1	61
509				1	1	1	0	1	0	1	1	1	1	0	0	63
510				0	1	0	0	1	0	0	1	0	1	0	1	40
511				0	0	0	1	1	0	0	0	1	1	1	1	50
512				0	1	1	0	1	0	0	0	1	0	0	1	36
513				0	0	0	1	1	1	1	1	0	1	1	1	68
514				0	1	1	1	1	0	1	0	0	1	1	1	61
515				0	1	1	0	1	0	1	1	0	1	0	0	49
516				1	0	1	1	0	0	0	1	1	1	1	0	53
517				0	0	0	1	0	1	0	0	1	0	1	1	41
518				1	0	1	0	1	0	0	0	0	0	1	1	33
519				1	1	0	1	1	1	1	1	1	0	0	0	64
520				0	1	0	1	0	0	1	1	0	1	0	0	41
521				0	1	1	0	0	0	0	0	0	1	1	1	37
522				1	0	1	1	0	1	1	0	0	0	0	1	39
523				1	1	1	1	0	0	1	0	0	0	0	0	31
524				1	1	1	1	1	1	1	0	0	1	1	0	69
525				1	1	0	0	0	0	1	0	0	1	0	0	30
526				0	0	1	1	1	0	1	0	0	1	1	1	54
527				1	0	1	0	0	1	0	0	1	0	1	0	39
528				0	0	1	1	0	0	0	0	1	1	1	0	40
529				0	1	1	0	0	0	0	0	1	0	1	0	32
530				1	0	1	0	0	1	1	0	1	0	1	1	53
531				1	1	0	0	1	0	1	1	0	0	1	1	54
532				0	0	0	1	0	1	1	1	0	1	0	0	43
533				0	1	0	0	0	0	0	0	0	1	1	0	27
534				1	1	1	0	0	1	1	1	1	0	0	0	54
535				0	1	0	0	1	1	0	1	0	1	1	1	60
536				0	1	0	1	1	1	0	1	0	1	1	1	66
537				1	0	0	1	0	1	1	0	1	0	0	0	39
538				0	0	1	1	0	1	1	1	0	1	1	1	63
539				1	1	0	1	1	1	0	0	0	0	0	1	41
540				1	1	1	0	0	1	0	1	1	1	0	1	60
541				0	1	1	1	1	0	1	0	0	1	1	0	55
542				0	0	1	1	1	0	1	1	1	1	0	0	57
543				0	1	1	1	0	0	0	0	0	0	0	0	18
544				1	0	0	1	0	1	0	1	0	1	1	0	49
545				1	0	1	0	0	1	0	0	1	1	1	1	54
546				1	0	1	1	0	0	0	1	0	1	0	0	34
547				0	1	1	1	1	1	0	1	0	0	0	1	51
548				1	0	1	1	0	0	0	1	0	0	1	1	39
549				1	0	0	1	1	0	1	0	1	1	1	0	58
550				1	0	1	1	1	0	0	1	0	0	0	0	33
551				1	1	1	0	1	0	0	1	1	1	0	1	59
552				0	0	1	0	0	0	0	0	0	1	1	0	24
553				0	0	0	0	0	0	0	1	0	1	0	0	19
554				0	1	1	0	0	0	1	1	1	1	0	0	49
555				1	1	1	0	1	1	0	1	0	0	0	0	44
556				0	0	0	1	0	0	1	1	0	0	0	1	29
557				1	0	0	1	1	1	1	0	1	1	1	1	73
558				0	1	1	0	0	1	1	1	0	1	0	1	55
559				1	0	1	0	1	0	0	1	0	0	0	1	33
560				1	1	0	0	0	1	1	1	0	1	0	0	49
561				1	0	0	0	0	1	1	1	1	0	1	1	57
562				0	1	1	0	0	1	0	0	0	0	0	0	22
563				1	1	0	1	1	1	1	0	0	0	0	1	50
564				0	0	0	1	1	1	0	1	1	0	1	1	59
565				1	1	0	0	0	0	1	1	1	0	0	0	39
566				0	0	1	1	1	0	0	0	1	0	1	0	40
567				1	0	0	1	1	1	0	1	1	1	0	0	58
568				1	0	1	0	1	0	1	1	1	0	0	1	52
569				0	1	1	1	1	0	0	1	0	0	1	1	51
570				0	1	1	1	1	0	1	1	1	1	1	1	80
571				0	0	0	1	1	0	0	1	0	1	0	1	39
572				1	1	0	0	0	0	0	1	1	1	0	1	45
573				1	1	1	1	0	0	0	1	1	1	0	1	56
574				1	1	1	1	1	1	0	1	0	0	0	0	50
575				0	1	0	1	0	0	0	1	1	1	1	0	51
576				1	1	0	0	0	1	0	0	1	1	0	0	41
577				1	0	0	1	1	0	0	1	0	1	0	0	38
578				0	0	0	0	0	0	1	1	0	1	0	0	28
579				0	1	1	1	0	1	1	1	1	1	0	1	70

580				1	0	1	1	1	0	0	0	0	0	1	1	39
581				1	1	1	0	0	1	0	0	1	0	0	0	36
582				0	0	1	0	1	0	0	0	0	0	1	0	24
583				1	0	0	1	0	1	0	1	1	1	1	0	59
584				0	1	0	0	1	0	1	1	0	1	1	1	59
585				1	0	0	1	0	1	0	1	1	1	0	0	49
586				1	0	1	0	1	1	1	1	1	0	0	0	56
587				0	0	1	0	1	1	1	1	0	0	0	1	47
588				0	1	0	1	1	0	0	0	1	0	0	1	37
589				0	0	0	1	1	1	0	1	1	1	1	1	69
590				0	1	1	0	1	0	1	0	1	1	0	0	49
591				1	0	1	0	1	1	0	0	0	0	0	1	33
592				0	0	1	0	0	1	1	0	0	1	1	1	48
593				1	1	1	1	1	1	0	0	0	0	1	1	56
594				0	1	1	1	0	0	0	1	0	1	0	0	36
595				1	1	1	1	0	1	0	0	1	1	1	0	61
596				1	0	1	0	1	0	0	1	0	1	1	0	47
597				1	1	0	0	1	1	1	0	1	1	0	0	59
598				0	0	1	0	1	0	1	1	1	0	0	0	42
599				1	0	1	1	0	1	1	1	1	0	1	1	68
600				1	0	0	0	1	1	1	1	0	0	1	1	57
601				0	1	1	0	1	0	0	1	1	1	1	1	65
602				1	1	0	1	1	0	1	1	1	0	1	1	69
603				0	1	1	1	0	0	1	1	1	0	1	0	56
604				1	0	1	0	1	1	1	1	1	1	1	1	81
605				0	0	1	0	0	0	1	1	0	0	0	0	23
606				0	1	0	0	1	1	1	1	0	0	1	1	59
607				0	0	0	0	0	1	1	1	1	0	0	0	38
608				1	1	1	0	0	1	1	1	0	0	1	1	59
609				1	0	0	0	1	1	0	0	0	1	1	1	48
610				0	0	0	0	1	1	1	1	0	1	0	0	47
611				1	1	1	0	0	0	0	0	0	1	0	0	26
612				1	1	1	1	0	1	0	1	0	0	1	1	56
613				0	1	1	0	1	0	0	1	0	1	0	0	40
614				0	0	1	0	1	1	0	1	0	1	0	1	48
615				1	0	0	0	1	0	0	0	0	1	0	1	28
616				0	0	1	1	0	0	0	1	1	0	1	1	45
617				1	1	0	0	1	0	1	0	0	1	0	1	44
618				0	1	1	0	0	1	1	0	0	1	1	1	55
619				1	0	1	0	0	1	0	1	0	1	0	0	38
620				0	0	1	1	0	0	0	1	1	0	0	0	30
621				1	1	1	1	1	1	1	0	0	0	0	0	50
622				0	1	1	1	0	1	0	0	1	1	0	0	47
623				0	1	0	1	0	0	0	1	0	0	1	1	37
624				1	1	0	0	0	1	1	0	0	0	0	1	35
625				0	1	0	0	1	1	1	0	0	1	1	0	55
626				1	0	1	1	1	1	1	0	1	1	0	0	62
627				1	1	0	0	1	1	1	0	0	0	1	1	54
628				1	0	1	1	0	1	1	0	1	0	0	0	44
629				0	0	0	0	1	1	1	0	1	1	0	0	48
630				0	0	1	0	0	0	1	1	1	0	0	0	33
631				0	0	1	0	1	1	0	1	1	0	0	0	43
632				0	1	0	0	1	0	0	0	0	1	0	0	26
633				0	0	0	0	1	0	0	1	1	0	0	0	28
634				1	1	1	1	1	0	0	1	0	1	0	1	55
635				0	1	0	0	0	0	0	1	0	1	0	1	31
636				1	0	0	1	1	0	1	1	0	0	0	1	43
637				0	0	1	1	1	0	1	0	0	0	1	1	44
638				1	0	0	1	0	1	1	1	1	0	0	0	48
639				1	1	0	0	0	1	1	0	1	0	0	1	45
640				0	0	1	0	1	0	0	1	0	1	1	0	43
641				1	0	1	1	1	1	0	1	1	0	1	1	68
642				0	1	0	0	0	1	1	1	0	0	0	1	40
643				1	0	1	1	1	0	1	1	0	1	0	1	57
644				0	1	1	1	1	0	0	1	1	1	1	1	71
645				1	1	1	1	1	0	0	1	1	0	0	1	55
646				0	1	0	0	1	0	0	1	1	0	0	0	35
647				0	0	1	0	0	0	0	0	0	1	1	1	30
648				1	1	0	0	1	0	1	0	0	1	0	0	39
649				1	1	1	1	1	0	1	1	1	1	1	0	79
650				0	0	1	1	1	1	0	1	0	0	0	1	44
651				1	1	1	0	0	0	0	0	1	1	1	1	51
652				0	1	0	0	1	0	0	1	0	0	0	1	31
653				1	1	1	0	0	0	0	1	1	0	1	1	50
654				1	1	0	0	0	1	0	1	0	0	1	0	40
655				1	0	0	0	1	0	1	0	1	0	0	1	38
656				1	1	0	1	1	1	0	1	0	1	0	0	55
657				0	1	0	0	0	1	0	0	0	0	1	0	27
658				1	0	1	1	1	1	1	1	1	1	1	1	87
659				1	0	0	0	1	1	0	1	1	0	1	0	52
660				1	1	0	1	0	0	0	1	0	1	0	0	36
661				1	1	1	0	1	0	1	0	1	0	1	1	59
662				0	0	0	1	0	1	0	1	0	0	1	1	40
663				0	0	0	1	1	1	0	0	0	0	1	1	40
664				1	0	0	1	0	1	1	0	0	1	0	0	38
665				0	0	0	1	0	1	0	0	1	1	1	1	50
666				1	0	1	1	1	0	0	0	1	0	1	1	49
667				0	1	0	0	1	0	1	0	1	1	0	1	50
668				0	0	1	0	0	0	1	0	1	1	0	0	33
669				1	1	1	0	0	0	1	1	0	0	0	0	34
670				1	1	1	1	0	0	1	1	0	0	0	1	45
671				0	0	1	0	0	0	1	0	0	0	0	0	14
672				0	0	0	0	0	1	0	0	1	1	1	0	39
673				0	0	1	0	0	1	0	0	0	1	1	1	40
674				0	1	1	0	1	1	0	1	1	1	1	1	75
675				0	1	0	1	1	1	0	1	1	1	0	1	66
676				0	0	1	0	1	0	0	0	0	0	0	1	19
677				1	1	1	1	1	1	0	0	1	0	1	1	66
678				1	0	1	0	1	1	0	0	0	1	0	1	43
679				0	1	0	1	1	1	1	0	0	1	0	1	56
680				0	1	0	1	1	1	0	0	1	0	1	0	52
681				1	1	0	1	0	1	0	0	1	0	0	1	42
682				1	0	1	0	1	0	1	1	0	1	1	1	61
683				1	0	1	0	0	1	1	1	0	0	1	1	52
684				1	0	0	1	1	0	1	0	0	0	0	0	28
685				1	0	0	1	0	0	1	0	0	1	1	0	38
686				1	0	1	1	1	0	1	0	0	0	0	1	38
687				0	0	1	1	1	0	1	0	1	0	0	0	39
688				0	0	1	1	0	1	0	0	0	0	1	0	30
689				1	1	1	0	1	1	1	1	0	1	1	1	78
690				1	1	1	0	1	0	0	0	1	1	0	0	45
691				1	0	0	1	1	1	1	0	0	1	1	1	63
692				0	0	0	1	0	0	1	0	1	1	1	1	49
693				0	1	0	0	0	0	1	1	0	0	1	1	40
694				1	1	0	1	0	0	0	0	0	1	1	1	42
695				1	1	0	0	0	0	1	0	1	1	0	0	40
696				1	1	0	1	1	0	0	0	0	0	1	1	41
697				1	1	1	1	0	1	0	0	1	0	0	1	47

698				0	1	0	1	0	0	1	1	1	1	0	0	50
699				1	0	0	0	0	1	1	0	1	1	1	1	58
700				1	0	1	1	1	0	1	0	0	0	1	1	48
701				0	1	1	0	0	0	1	1	0	1	0	1	45
702				0	0	1	1	0	0	0	1	0	1	0	1	35
703				1	0	1	1	1	0	0	0	0	1	0	0	34
704				1	0	0	0	1	0	0	0	0	0	1	1	29
705				1	1	1	1	1	0	0	1	1	0	1	0	60
706				0	0	0	1	1	1	0	1	0	0	0	0	34
707				0	0	0	1	0	1	0	0	1	0	1	0	36
708				0	0	0	1	0	0	1	0	1	0	1	1	40
709				1	0	0	0	0	1	0	0	1	1	0	0	34
710				1	1	0	0	0	1	1	1	0	1	1	1	64
711				1	0	0	1	0	1	0	1	0	0	0	0	29
712				0	1	1	0	1	1	1	0	1	0	0	0	50
713				1	1	0	1	1	1	1	0	1	0	1	1	70
714				1	0	0	1	1	1	0	0	0	1	1	1	54
715				0	0	0	0	0	1	1	1	0	0	1	1	43
716				1	0	0	1	0	1	1	0	0	0	0	1	34
717				1	1	0	0	0	0	0	1	0	0	0	1	26
718				0	1	1	1	1	0	0	0	0	0	1	0	37
719				0	0	1	1	0	0	1	1	1	0	0	0	39
720				0	1	1	0	1	1	1	1	1	1	0	1	74
721				0	0	0	1	0	1	1	0	1	1	0	0	44
722				1	0	0	1	1	1	1	0	0	1	0	1	53
723				0	0	1	1	1	0	0	1	0	1	0	0	39
724				0	0	0	0	0	0	1	0	1	0	0	1	24
725				1	1	0	0	0	0	0	0	0	0	1	0	21
726				1	1	0	1	1	0	1	1	0	0	1	0	54
727				0	0	1	0	1	0	0	0	0	1	1	0	34
728				1	0	1	0	0	1	0	0	1	0	0	0	29
729				0	1	0	0	1	0	0	0	0	0	0	1	22
730				0	1	1	1	0	1	0	0	1	1	0	0	47
731				1	1	1	0	0	1	0	0	1	0	0	1	41
732				1	0	0	1	0	0	0	0	1	1	0	1	35
733				0	1	1	1	0	0	0	0	1	0	0	0	28
734				0	0	1	0	1	0	0	1	1	0	1	0	43
735				1	0	0	1	1	0	1	1	0	1	0	0	47
736				0	0	1	1	1	0	0	1	1	1	1	1	64
737				1	0	0	0	1	1	1	1	1	1	1	0	71
738				1	1	1	0	0	1	0	1	1	1	1	0	65
739				0	1	1	1	0	1	1	0	0	0	0	1	42
740				1	0	0	0	0	0	1	1	0	0	1	0	32
741				1	1	1	0	1	1	0	1	1	0	0	0	54
742				1	0	0	1	0	1	0	0	1	1	0	1	45
743				1	0	1	1	1	1	0	0	0	0	0	0	34
744				1	1	1	1	0	1	1	1	1	0	1	0	70
745				1	1	1	1	1	1	1	1	1	1	1	1	94
746				1	0	0	1	1	0	1	0	0	1	0	1	43
747				1	1	1	0	0	1	1	1	0	1	1	1	69
748				0	0	1	0	0	1	1	1	1	1	0	0	52
749				0	1	1	1	1	0	0	1	1	0	1	1	61
750				0	0	1	1	0	0	1	1	0	1	0	1	44
751				1	0	0	1	1	1	0	1	1	1	1	1	73
752				0	0	1	0	1	0	0	0	1	0	0	1	29
753				0	1	1	0	0	0	0	0	0	1	1	0	31
754				0	0	0	1	0	0	0	1	0	1	0	1	30
755				1	0	0	0	1	1	1	0	1	1	1	1	67
756				0	0	1	0	1	0	1	1	1	0	0	1	47
757				0	0	1	0	0	1	1	1	1	1	1	1	68
758				1	1	0	0	1	1	0	1	1	1	1	1	74
759				1	0	0	1	1	1	0	0	1	0	1	0	49
760				1	0	0	1	1	1	1	1	0	1	0	1	62
761				1	0	1	1	0	0	0	0	0	1	1	0	34
762				1	1	1	1	0	0	0	0	1	0	0	0	32
763				0	0	1	0	1	1	0	0	1	1	0	1	49
764				1	0	1	0	1	1	1	0	0	1	1	1	62
765				1	1	0	1	1	0	1	1	0	1	1	1	69
766				0	1	0	0	1	0	1	1	1	0	1	0	54
767				0	0	1	0	0	1	1	0	1	0	1	0	43
768				0	0	0	1	0	1	1	0	0	0	1	1	40
769				0	1	1	0	1	0	0	0	0	1	0	1	36
770				1	1	0	1	1	0	0	0	0	1	0	0	36
771				1	1	0	1	1	0	0	1	0	1	1	1	60
772				0	0	0	1	0	1	0	1	0	0	0	0	25
773				1	0	0	1	1	0	1	0	0	0	1	1	43
774				1	1	0	0	1	1	1	0	1	1	1	1	74
775				1	0	1	1	1	1	1	0	0	1	0	0	52
776				1	1	1	1	0	1	1	0	1	0	1	1	66
777				0	1	0	0	1	0	0	1	1	1	1	0	55
778				0	0	1	1	1	0	0	1	1	1	1	0	58
779				1	0	1	0	0	0	0	0	1	1	1	0	38
780				1	1	1	0	0	1	0	0	0	1	0	1	41
781				1	1	0	1	1	0	1	0	1	0	0	1	50
782				0	0	1	1	1	1	0	1	1	0	1	0	59
783				1	0	0	0	1	0	1	0	0	1	0	1	37
784				0	0	1	0	0	0	0	0	1	1	0	0	24
785				0	1	1	1	1	0	1	0	0	1	1	1	61
786				1	0	1	1	0	0	0	1	1	0	0	0	34
787				0	0	0	1	1	1	0	0	0	0	0	1	30
788				1	1	0	0	0	0	0	1	0	0	1	1	36
789				0	1	0	0	1	1	1	1	0	1	0	1	59
790				1	1	1	0	0	0	1	1	0	0	0	0	34
791				1	0	0	1	1	1	1	0	1	0	0	0	48
792				0	1	1	0	1	1	0	0	0	1	1	0	50
793				0	1	1	0	0	0	0	1	1	0	1	1	46
794				1	1	0	1	0	0	0	1	0	0	0	1	32
795				0	1	0	0	0	1	1	0	1	0	1	0	46
796				0	0	0	0	1	0	0	0	1	1	1	1	44
797				0	1	0	1	1	1	0	0	0	1	1	0	51
798				0	1	0	0	1	0	1	1	0	0	1	1	50
799				0	1	1	0	1	0	0	0	1	0	0	0	31
800				0	1	0	1	0	0	1	1	0	1	0	0	41
801				0	1	0	0	0	0	1	0	0	0	0	1	21
802				0	1	1	0	0	0	0	1	0	1	1	0	41
803				0	0	0	1	1	1	1	0	1	0	1	1	59
804				0	1	0	1	1	1	1	0	0	1	0	1	56
805				1	0	1	0	1	1	0	0	0	0	0	0	28
806				1	1	0	0	0	0	0	0	0	1	1	1	36
807				1	1	1	1	1	1	0	1	1	1	0	0	70
808				1	0	1	0	1	1	0	1	0	0	0	1	43
809				1	1	1	1	1	1	1	1	1	0	0	1	74
810				1	0	0	0	0	1	0	1	0	1	0	1	38
811				1	0	1	0	1	0	1	1	0	0	0	0	36
812				0	0	1	0	1	0	0	1	0	1	1	0	43
813				0	1	1	0	0	1	1	1	1	1	1	1	75
814				1	0	1	1	0	0	0	1	0	0	1	1	39
815				1	1	0	0	1	0	0	1	1	0	0	1	45

816				1	1	0	0	1	1	1	0	1	0	1	0	59
817				1	1	0	1	0	0	0	0	0	1	1	1	42
818				1	1	0	0	1	0	0	0	1	1	1	1	55
819				1	0	0	0	1	0	1	1	0	1	0	1	46
820				0	1	1	1	0	1	1	1	0	0	1	1	61
821				0	1	0	0	0	0	1	1	0	1	0	0	35
822				1	0	1	1	0	1	1	1	0	0	0	0	43
823				1	0	1	1	0	0	0	1	1	0	0	1	39
824				1	0	0	1	1	0	1	1	0	1	0	0	47
825				0	0	0	0	0	0	0	1	1	0	1	0	29
826				1	0	0	1	1	0	1	0	0	0	0	1	33
827				1	0	0	0	1	0	0	0	0	1	0	1	28
828				1	1	1	1	1	0	1	1	0	1	1	1	74
829				0	0	1	1	1	1	0	1	0	0	1	1	54
830				0	1	1	1	1	0	1	0	1	0	1	0	56
831				0	0	0	1	1	1	0	1	1	0	0	1	49
832				0	1	0	0	1	1	0	0	0	1	0	1	41
833				1	0	1	1	1	1	1	0	0	0	0	0	43
834				1	0	1	1	0	0	0	1	1	0	1	0	44
835				0	1	1	1	0	0	0	1	0	1	1	0	46
836				0	0	0	1	0	1	0	1	0	1	0	1	40
837				0	0	0	1	0	1	1	0	1	0	0	1	40
838				1	0	1	1	1	0	1	1	1	1	1	1	77
839				1	0	1	1	1	1	1	0	1	0	0	0	53
840				1	0	1	0	1	1	0	0	1	1	0	0	48
841				1	1	1	1	1	1	1	1	0	0	0	1	64
842				0	0	1	1	0	0	0	1	1	1	1	1	55
843				0	0	0	1	0	1	1	0	0	1	0	0	34
844				0	1	0	1	0	1	1	0	0	0	0	1	37
845				1	0	0	1	1	1	0	1	1	0	1	1	63
846				1	0	1	1	0	0	0	1	1	0	0	0	34
847				0	0	1	1	1	0	0	0	0	1	1	0	39
848				1	0	1	1	1	0	0	1	0	0	0	0	33
849				1	0	0	0	0	0	0	0	1	0	1	0	24
850				0	1	1	0	0	0	0	0	0	1	0	1	27
851				1	0	0	0	0	1	0	0	0	0	1	1	29
852				1	1	0	1	1	1	0	1	0	0	1	0	55
853				1	0	1	1	1	0	0	1	1	1	0	0	53
854				1	0	0	0	1	1	1	0	1	1	1	1	67
855				0	0	0	1	1	0	1	0	1	0	1	1	49
856				0	0	1	0	0	1	1	1	0	0	1	1	48
857				1	1	1	1	0	1	0	1	0	1	0	1	56
858				0	0	1	0	0	1	1	0	1	1	1	1	58
859				1	1	0	1	1	1	1	1	1	0	1	1	79
860				1	1	1	0	1	0	0	1	1	0	0	0	44
861				0	0	1	0	0	1	0	0	1	1	1	0	44
862				0	1	1	1	1	1	0	0	0	1	1	1	62
863				1	1	1	0	1	1	0	1	0	1	0	0	54
864				0	1	0	0	1	1	1	1	0	0	0	0	44
865				0	0	1	1	1	0	0	0	0	1	0	1	35
866				0	1	1	1	0	0	0	1	0	0	0	1	32
867				1	0	0	1	0	1	1	0	0	0	0	1	34
868				0	0	0	0	0	1	1	1	1	0	0	0	38
869				1	0	1	1	0	0	0	1	0	1	1	0	44
870				0	0	1	0	1	0	0	0	1	1	1	1	49
871				1	0	0	1	1	1	0	1	0	1	0	0	48
872				0	1	1	1	1	0	0	1	1	0	1	0	56
873				0	0	1	0	1	1	1	1	1	0	0	0	52
874				1	1	1	0	1	1	0	0	0	0	1	1	50
875				1	0	0	0	1	1	0	1	1	0	1	1	58
876				1	1	0	1	1	1	0	1	0	1	0	0	55
877				0	0	0	0	1	1	1	1	0	1	1	1	62
878				0	0	0	1	1	0	0	0	0	1	0	1	30
879				0	1	1	0	1	1	1	0	0	1	1	1	65
880				0	0	1	0	1	0	0	0	1	0	1	0	34
881				0	1	0	1	0	1	1	0	1	0	1	0	51
882				1	1	0	1	0	0	0	1	0	0	1	0	36
883				0	0	1	1	0	1	1	1	1	0	0	1	54
884				0	0	1	0	0	0	1	0	0	0	0	0	14
885				1	1	1	0	1	1	0	0	0	1	1	0	55
886				1	1	1	1	0	1	0	0	1	0	1	1	57
887				0	1	1	1	0	0	1	0	0	0	0	0	27
888				1	0	0	0	1	0	0	0	0	0	0	1	19
889				1	1	0	1	1	1	0	1	0	1	0	1	60
890				1	0	0	1	0	1	0	0	0	0	1	1	35
891				1	0	1	0	1	1	1	0	0	1	0	0	47
892				0	1	0	1	0	1	1	0	0	0	1	1	47
893				1	1	0	0	1	1	0	0	0	1	1	0	50
894				0	1	1	0	1	0	0	0	1	0	1	0	41
895				1	1	1	1	0	1	0	0	1	0	0	1	47
896				1	1	0	1	0	1	1	1	0	0	1	0	55
897				1	0	0	1	0	0	0	0	0	1	0	1	25
898				1	1	1	1	0	1	0	0	1	1	0	1	57
899				1	1	1	0	1	0	0	0	1	0	0	0	35
900				0	0	1	0	0	1	0	1	1	0	1	1	49
901				1	0	1	1	1	1	1	0	1	0	1	1	68
902				0	1	1	1	1	1	0	0	0	1	0	1	52
903				1	1	1	0	0	1	0	1	1	0	0	1	50
904				0	1	1	1	0	1	0	1	1	0	0	0	47
905				0	1	1	1	1	0	1	1	0	0	0	0	45
906				0	1	1	0	1	1	1	0	0	1	0	0	49
907				0	1	1	0	1	0	1	1	1	1	0	1	64
908				0	1	0	0	0	0	0	1	1	0	1	0	36
909				1	0	0	1	1	1	1	0	1	0	0	1	53
910				0	0	0	1	1	0	0	0	1	1	1	1	50
911				0	1	1	0	1	0	0	0	1	1	0	1	46
912				0	1	1	1	1	0	0	0	1	0	0	1	42
913				0	0	1	0	1	0	1	0	0	1	0	1	38
914				0	1	0	1	0	0	0	0	0	0	1	0	23
915				1	0	1	1	1	0	1	0	1	0	1	0	53
916				1	0	1	0	0	0	0	1	0	0	1	1	33
917				0	1	0	0	0	0	0	1	0	1	0	0	26
918				1	1	0	0	0	1	0	1	0	0	0	1	36
919				1	1	0	1	1	1	0	1	1	0	0	1	61
920				1	1	0	0	0	0	0	1	1	0	0	1	36
921				0	0	0	0	0	1	1	1	1	1	0	1	53
922				0	0	0	0	0	1	1	0	0	1	1	0	38
923				1	0	0	1	1	1	0	1	0	1	1	1	63
924				0	0	1	0	0	1	0	1	0	0	1	1	39
925				0	1	0	0	0	1	1	0	1	0	1	0	46
926				1	0	0	0	1	0	0	0	1	1	0	0	33
927				0	1	0	0	0	1	1	1	0	0	1	1	50
928				1	0	1	1	1	1	1	0	1	0	0	1	58
929				0	0	0	1	0	0	0	0	0	0	0	1	11
930				0	0	1	1	1	1	1	0	0	0	1	0	49
931				1	0	0	1	1	0	0	0	1	1	0	1	44
932				0	1	0	0	0	1	1	0	1	1	1	0	55
933				0	0	1	1	0	1	1	1	0	0	0	0	39

934				0	1	1	0	0	1	1	1	0	0	1	0	50
935				0	0	0	1	0	1	0	1	0	1	0	1	40
936				0	0	0	1	1	0	1	0	1	0	1	0	44
937				1	0	0	1	1	0	1	1	0	0	1	0	47
938				1	1	0	1	1	1	1	1	1	0	1	0	74
939				0	1	0	0	1	1	1	0	0	1	1	0	55
940				1	0	0	1	1	0	0	0	1	0	0	0	29
941				0	1	1	0	1	0	0	1	1	1	1	1	65
942				0	1	0	0	1	1	0	1	0	0	0	1	41
943				0	0	0	1	1	1	1	0	0	0	1	0	44
944				0	0	1	0	1	1	0	1	0	1	0	1	48
945				1	1	1	1	1	0	1	0	0	1	0	1	55
946				1	0	1	0	0	0	0	0	1	1	1	0	38
947				1	1	0	1	1	1	1	0	0	0	1	1	60
948				0	1	0	1	1	1	1	1	0	0	1	0	60
949				1	1	1	1	0	1	0	1	0	0	1	0	51
950				1	0	1	1	1	0	1	1	1	1	1	1	77
951				1	0	1	1	1	0	1	1	0	0	1	1	57
952				1	0	1	0	1	1	1	1	0	0	1	0	56
953				0	0	1	0	1	0	1	0	0	1	1	1	48
954				0	0	1	0	1	1	1	0	0	1	0	1	48
955				0	0	0	1	0	0	1	0	0	0	1	1	30
956				1	0	1	1	0	0	0	1	1	0	1	0	44
957				0	0	0	1	0	0	1	0	1	1	0	0	34
958				1	0	1	1	0	0	0	1	1	0	0	0	34
959				1	0	1	1	1	0	0	1	0	0	0	0	33
960				0	1	1	1	0	0	0	1	1	0	0	0	37
961				1	1	1	0	0	1	0	1	0	1	0	0	45
962				1	0	1	1	0	0	1	1	0	1	0	1	48
963				0	1	0	0	1	1	0	1	0	1	1	1	60
964				0	0	0	0	1	1	0	0	0	1	1	0	39
965				1	0	1	1	0	0	1	1	1	1	1	1	68
966				1	1	0	0	1	1	1	0	0	1	0	0	49
967				0	1	0	0	0	1	0	1	0	0	1	0	36
968				0	0	0	0	1	0	1	0	1	0	0	1	33
969				1	1	1	0	1	0	0	1	0	0	0	0	34
970				1	1	1	1	0	1	0	0	1	0	1	1	57
971				1	1	1	1	1	0	1	0	0	0	1	0	50
972				0	0	0	0	1	1	0	1	1	1	1	1	63
973				0	0	0	1	1	1	1	1	1	0	0	1	58
974				0	1	1	0	1	1	1	1	1	0	0	1	64
975				1	1	1	1	0	1	1	1	1	0	1	0	70
976				1	1	0	0	1	1	0	0	1	0	0	0	40
977				1	0	0	0	1	0	0	0	1	0	0	0	23
978				1	1	1	1	0	1	0	0	0	1	0	1	47
979				1	1	1	0	0	1	1	0	1	0	0	0	45
980				0	1	1	0	0	0	1	1	1	1	0	0	49
981				0	1	0	1	1	1	0	1	0	1	0	0	51
982				1	1	1	1	0	0	1	1	1	1	0	0	59
983				1	0	1	0	1	1	0	1	0	1	0	1	52
984				1	1	1	1	0	1	0	1	0	1	1	0	60
985				1	0	1	1	1	0	1	0	1	1	1	0	62
986				0	0	1	1	0	0	1	0	1	1	1	0	49
987				1	0	1	0	1	0	0	1	1	1	1	0	57
988				0	0	0	1	1	1	0	1	0	0	0	0	34
989				1	0	0	0	0	1	1	0	1	0	1	1	48
990				0	1	0	0	1	0	0	0	1	1	1	1	51
991				1	0	1	1	0	0	1	0	0	0	0	1	29
992				0	0	0	1	1	1	0	1	0	1	0	0	44
993				0	1	1	0	1	0	1	1	1	1	1	1	74
994				1	1	1	0	1	1	0	1	1	1	1	1	79
995				1	0	0	1	1	1	1	0	1	0	1	0	58
996				1	1	1	1	0	0	0	0	0	1	0	1	37
997				0	0	1	0	1	0	1	0	1	0	0	0	33
998				1	1	1	1	1	0	1	0	0	0	0	1	45
999				0	0	0	0	1	0	1	1	1	1	0	1	52
1000				0	1	0	0	1	1	1	0	1	1	1	0	64
1001				0	1	0	1	1	0	1	1	1	1	0	1	65
1002				0	1	1	1	0	1	0	0	0	0	1	0	37
1003				0	0	1	1	1	1	1	0	1	0	1	1	64
1004				1	0	0	1	0	1	0	0	0	1	0	0	30
1005				1	1	0	1	0	0	1	1	0	1	1	1	60
1006				1	0	0	1	0	1	1	0	1	0	1	1	54
1007				1	1	0	1	1	1	1	0	1	1	0	0	65
1008				0	1	1	0	0	1	1	0	1	0	0	0	41
1009				1	0	0	0	0	0	1	0	0	1	1	0	33
1010				1	0	0	1	1	0	1	1	1	1	0	1	62
1011				1	1	1	1	0	0	1	1	1	0	1	0	60
1012				0	1	1	1	1	0	0	0	1	1	1	1	62
1013				0	0	0	0	1	1	1	0	1	0	1	0	48
1014				0	1	1	0	0	1	0	1	1	1	1	1	66
1015				1	1	1	1	0	1	1	1	0	1	0	1	65
1016				1	1	0	0	0	1	1	0	0	0	1	0	40
1017				1	1	0	0	1	0	0	1	1	1	0	0	49
1018				1	1	1	1	0	0	0	1	1	1	1	1	66
1019				1	1	0	1	1	1	0	0	1	1	1	0	66
1020				0	1	1	1	0	1	0	1	1	1	1	1	71
1021				0	1	1	1	0	0	0	0	0	1	1	1	42
1022				1	0	1	0	1	0	1	1	0	1	1	0	56
1023				1	0	1	0	0	1	1	1	0	0	1	0	47
1024				1	0	0	1	1	1	0	1	0	0	0	1	44
1025				1	0	1	0	0	0	1	0	0	1	1	0	37
1026				0	0	1	1	0	1	1	0	1	1	1	0	59
1027				0	0	0	0	1	0	1	0	1	1	1	1	53
1028				1	0	0	1	1	0	0	1	1	1	1	1	63
1029				0	0	0	0	1	1	0	1	0	0	0	1	34
1030				0	1	1	0	0	1	1	0	0	1	0	0	40
1031				1	1	1	0	1	0	0	0	0	0	0	0	25
1032				1	1	1	1	0	0	0	0	1	1	1	1	57
1033				0	1	1	1	1	1	0	0	0	1	1	1	62
1034				1	0	0	1	0	1	1	0	0	0	1	1	44
1035				1	0	1	1	1	0	1	1	1	1	0	0	62
1036				1	1	1	1	0	1	0	0	1	1	1	0	61
1037				1	0	0	1	0	0	0	1	0	1	1	0	39
1038				1	0	1	0	1	0	0	0	0	1	0	1	33
1039				0	0	0	1	1	1	1	1	0	1	1	1	68
1040				1	0	1	0	1	1	0	0	1	1	1	1	63
1041				1	0	0	1	1	0	0	0	0	0	1	0	29
1042				0	0	1	1	1	1	1	1	0	0	0	1	53
1043				1	0	1	0	0	1	0	1	1	0	1	1	53
1044				1	0	0	1	1	1	1	0	1	1	0	0	58
1045				0	1	0	0	0	0	1	0	0	1	0	1	31
1046				1	1	0	0	0	0	0	1	0	1	1	0	40
1047				0	0	0	0	0	1	1	1	1	0	0	0	38
1048				0	1	0	1	0	1	1	0	1	1	1	1	66
1049				1	0	0	0	1	0	0	0	1	0	0	0	23
1050				1	0	1	0	1	0	0	1	0	0	1	0	37
1051				0	1	1	0	1	1	0	1	0	0	0	1	45
1052				1	0	0	1	0	0	1	1	1	1	0	1	53
1053				1	1	0	0	1	1	0	1	0	1	0	0	49
1054				1	1	0	1	0	0	1	1	1	0	1	1	60

1055			1	0	0	0	0	0	0	1	0	0	1	1	29
1056			0	1	0	0	0	0	0	1	1	1	0	0	36
1057			0	1	0	1	0	1	0	0	0	1	1	0	42
1058			1	0	0	0	0	0	0	1	1	1	0	0	33
1059			1	0	1	0	0	0	1	0	1	1	0	1	43
1060			1	0	0	1	0	0	0	0	1	0	0	1	25
1061			1	0	1	1	1	1	0	0	0	0	0	0	34
1062			1	0	1	1	0	1	0	1	0	1	0	1	49
1063			1	1	0	0	0	0	0	1	1	0	1	0	40
1064			1	1	0	0	1	0	0	0	1	1	0	0	40
1065			1	1	0	0	0	0	0	0	0	1	1	1	36
1066			1	0	0	1	1	1	0	1	0	0	0	0	38
1067			0	1	0	1	0	0	1	0	1	1	0	0	41
1068			0	1	1	0	0	0	1	1	1	1	1	0	59
1069			0	1	0	0	0	1	0	1	0	1	0	1	41
1070			1	1	1	0	1	0	0	0	0	0	1	0	35
1071			1	0	0	0	1	1	0	1	1	1	0	0	52
1072			0	0	1	1	1	0	0	0	0	0	0	0	20
1073			0	1	1	0	0	1	0	0	1	0	1	0	42
1074			0	0	0	1	1	0	1	1	1	0	1	1	58
1075			1	1	1	0	0	0	0	1	1	1	1	1	60
1076			1	0	0	0	0	0	1	0	0	0	0	1	18
1077			1	1	1	0	0	0	0	0	1	0	1	0	36
1078			1	0	1	1	1	0	0	1	1	0	1	1	58
1079			0	1	0	1	0	1	1	1	1	0	0	0	51
1080			0	0	0	1	0	0	0	0	1	0	1	1	31

QualiPresc - Quality of Prescription in Primary Health Care

Instructions
1. Select the indicator in the Table below; and 2. Indicate with an X, in the Table on the right, the month in which a new median begins.

Indicators
1. Patient's date of birth
2. Prescriber's identification
3. Record of allergy report
4. Medicine included in the institutional list officially approved
5.Active ingredient
6. Concentration
7. Dosage
8. Pharmaceutical form
9. Route of administration
10. Frequency of administration
11. Duration of treatment
12. Directions on the use of the drugs
13. Non-pharmacological recommendations
14. QUALITY

Month	Value	Median	New Median
jan/21			
fev/21			
mar/21			
abr/21			
mai/21			x
jun/21			
jul/21			
ago/21			
set/21			
out/21			x
nov/21			
dez/21			x
jan/22			
fev/22			
mar/22			
abr/22			
mai/22			
jun/22			
jul/22			
ago/22			
set/22			
out/22			
nov/22			
dez/22			
jan/23			
fev/23			
mar/23			
abr/23			
mai/23			
jun/23			
jul/23			
ago/23			
set/23			
out/23			
nov/23			
dez/23			

1. Patient's date of birth

Run Chart: 1. Patient's date of birth

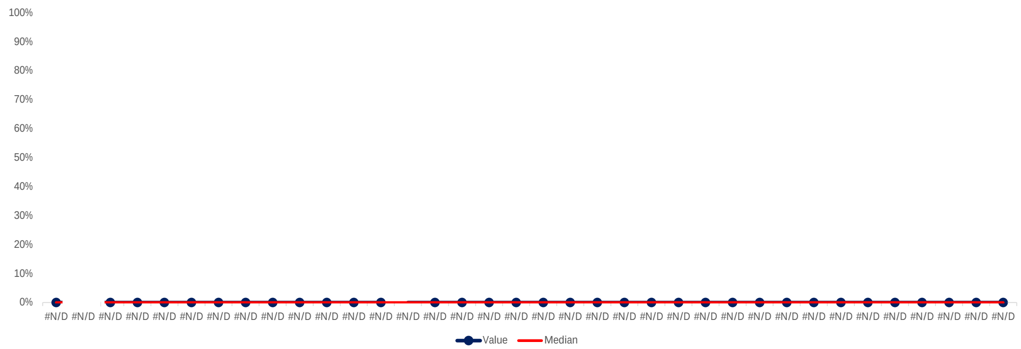

Supplement: S2 File — (PDF) [file pone.0267707.s005.pdf]
