# Supplementary material for: Validation of the QualiPresc instrument for assessing the quality of drug prescription writing in primary health care
Source: PLoS One. 2022 May 11;17(5):e0267707. doi: 10.1371/journal.pone.0267707 (PMC9094502; doi:10.1371/journal.pone.0267707)

QualiPresc - Calidad de la Prescripción en Atención Primaria de Salud

Instrucciones:

El instrumento QualiPresc tiene como objetivo facilitar el seguimiento de la calidad de las prescripciones de medicamentos en base a criterios de redacción. Se entiende por criterios de redacción los relacionados con la estructura del documento de prescripción en función de la información del usuario, prescriptor y medicamento prescrito.

Cómo utilizar:

1) El instrumento incluye 13 indicadores de calidad de prescripción de medicamentos que tienen diferentes pesos para la efectividad y seguridad de la prescripción.
2) QualiPresc debe ser aplicado preferentemente por farmacéuticos y/o previa formación impartida por este profesional.
3) Antes de recolectar datos, los evaluadores deben leer cuidadosamente las definiciones y aclaraciones para la medición de indicadores en la tabla a continuación.
4) Después de leer, haga clic en la siguiente hoja de trabajo llamada "recolección" o en la flecha al lado para registrar los datos de las recetas evaluadas.

INDICADOR	DEFINICIONES/ACLARACIONES PARA LA MEDICIÓN	SCORE[3]		
		S	E	Q
**1. Fecha de nacimiento del paciente**	Día / mes / año.	7,1	6,9	6,4
**2. Identificación del prescriptor**	Ampara número de registro profesional ante el Consejo Regional, profesión, nombre y apellido del prescriptor. Las palabras intermedias al nombre y apellido pueden estar constituidas por las letras iniciales.	6,9	4,8	4,2
**3. Registro de informe de alergia**	Registro de información sobre el hecho de que el usuario es alérgico a determinado(s) medicamento(s). Debe incluirse en la prescripción independientemente de que el fármaco de que se trate esté presente en la prescripción.	8,3	6,6	7
**4. Medicamento incluido en la lista institucional oficialmente aprobada[1]**	Componente de la lista estándar de medicamentos de una institución, estandarizados, preferentemente, de acuerdo con el perfil epidemiológico y la mejor evidencia científica de eficacia, seguridad y costo-efectividad. Como alternativa a la lista estándar de una institución específica, se puede utilizar como base la lista estándar de medicamentos esenciales municipales, estatales o nacionales, considerando la más adecuada al contexto.	6	6,3	4,8
**5. Principio activo**	Esta es la Denominación Común Brasileña (DCB) o, en su defecto, la Denominación Común Internacional (DCI). No utilice nombres de medicamentos	6,9	6,6	5,8
**6. Concentración[2]**	Corresponde a la cantidad de principio activo contenido en cada unidad de dosificación. Para formas de dosificación sólidas, 1 unidad de dosificación corresponde a 1 unidad de dosificación (por ejemplo, 1 tableta). Para formas de dosificación semisólidas y líquidas, 1 unidad de dosificación corresponde a 1 unidad de medida (por ejemplo, 1 ml, 1 g) (4,5).	8,3	8,7	9,2
**7. Dosis[2]**	Cantidad de medicamento a administrar en cada momento de uso. Consta de valor numérico y unidad de medida. Como unidad de medida, considerar el nombre de la forma farmacéutica sólida o gotas o abreviaturas/siglas/símbolos recomendados nacional y/o internacionalmente (4,5).	8,6	9	9,9
**8. Forma farmaceutica**	Forma física final del droga tras la mezcla de principios activos y excipientes durante el proceso de producción.	8,3	8,4	8,9
**9. Ruta de administración**	Puerta de entrada a través de la cual se administra el fármaco para llegar a su sitio de acción (por ejemplo, oral, intramuscular, intravenosa, etc.).	8,3	8,7	9,2
**10. Frecuencia de administración**	Número de veces, considerando un período de 24 horas, en que se debe administrar cada dosis de medicamento (p. ej., cada 12 horas, etc.).	8,6	9	9,9
**11. Duración del tratamiento**	Duración del tiempo en que se debe usar el medicamento (por ejemplo, durante 10 días, uso continuo, etc.).	8,3	9	9,6
**12. Directrices sobre el consumo de drogas[1]**	Mejor período para la administración da droga (p. ej., mañana/noche, antes/después de las comidas, en ayunas, después del baño), duración de la administración da droga (p. ej., n minutos, hasta que se termine el contenido del envase), aplicación en capa fina (p. ej., uso tópico) , dilución de drogas, instrucciones de uso de dispositivos de inhalación, entre otros necesarios para que el usuario comprenda cómo usar la droga.	8,6	9	9,9
**13. Recomendaciones no farmacológicas**	Información relativa a tratamientos no farmacológicos (por ejemplo, dieta, actividad física, etc.).	6	6,9	5,3

[1] En los indicadores 4 a 12, si la prescripción tiene más de 1 medicamento, este ítem se considera cumplido solo si todos los medicamentos de prescripción cumplen con el indicador.

[2] No use las abreviaturas U, u y UI, ya que pueden confundirse con 0 o 4 o cc o IV o 10, escriba unidad internacional. No use las abreviaturas mcg o µg, escriba microgramo. No use ceros al final ni omita los ceros al principio, ya que el lugar decimal puede pasar desapercibido, escriba X mg y 0.X mg, respectivamente. Para dosis o volúmenes con números fraccionarios (ej. 2.5mL), observar en ambos ejemplares de prescripción que la coma esté bien posicionada y clara. No utilice un punto en lugar de una coma [4,5].

[3] Puntuaciones de ponderación con respecto a Seguridad (S) y Efectividad (E), para ponderar el indicador compuesto Calidad (Q).

[4] The Joint Commission. Facts about Official Do-Not-Use List. Disponible: http://www.jointcommission.org/assets/1/18/Do_Not_Use_List.pdf. Consultado el: 18 de abril de 2021.

[5] Ministério da Saúde. Protocolo de segurança na prescrição, uso e administração de medicamentos. 2013. Disponible: https://proqualis.net/sites/proqualis.net/files/000002490IQmwD8.pdf.

QualiPresc - Calidad de la Prescripción en Atención Primaria de Salud

Recogida de datos:

1. Para garantizar un seguimiento continuo, seleccione un mínimo de 20 a 30 prescripciones. Hacer una selección aleatoria (sortear entre todas las recetas del período evaluado), para asegurar la representatividad.
2. Incluir solo recetas legibles y colocar el número de identificación de las incluidas en la primera columna posterior a la fecha de recolección. Si alguna es ilegible, extraiga otra receta del mismo período para completar la muestra.
3. Para las prescripciones incluidas, responda con 0 o 1 si los indicadores son cumplidos o no cumplidos, respectivamente.
4. Después de ingresar las respuestas, verifique los resultados consolidados de los 13 indicadores simples y los 3 indicadores compuestos de QualiPresc en la siguiente hoja de cálculo.

IDENTIFICACIÓN			INDICADORES SIMPLES													
ID	Fecha	Nº receta	1. Fecha de nacimiento del paciente	2. Identificación del prescriptor	3. Registro de informe de alergia	4. Medicamento incluido en la lista institucional oficialmente aprobada	5. Principio activo	6. Concentración	7. Dosis	8. Forma farmaceutica	9. Vía de administración	10. Frecuencia de administración	11. Duración del tratamiento	12. Informaciones sobre el uso del medicamento	13. Recomendaciones no farmacológicas	14. CALIDAD
1			1	0	1	0	1	0	1	0	1	1	0	1	1	63
2			0	0	1	0	0	1	0	1	1	0	0	0	1	40
3			0	0	1	1	1	0	0	1	1	1	0	0	0	46
4			0	0	1	1	1	1	1	0	1	1	1	1	0	75
5			1	0	1	0	0	1	0	0	0	0	1	1	0	42
6			0	0	1	0	0	1	1	1	0	0	1	0	0	45
7			1	0	0	0	1	1	1	0	0	1	1	0	1	56
8			1	1	0	0	0	0	0	1	1	0	0	1	0	39
9			1	1	0	1	1	1	0	1	1	0	0	1	1	64
10			0	0	1	0	1	1	0	0	0	1	1	1	1	57
11			1	0	1	1	1	0	1	0	0	0	1	1	1	59
12			0	1	1	1	0	1	0	0	1	0	0	1	1	50
13			0	1	0	1	1	0	1	1	1	1	0	0	1	58
14			1	1	1	0	0	0	1	1	0	1	1	0	0	56
15			1	1	0	0	1	1	0	0	0	1	0	0	1	41
16			1	1	1	0	0	0	0	1	0	1	1	0	1	51
17			1	1	0	0	1	1	0	1	0	1	0	1	0	54
18			1	0	0	0	0	0	1	1	1	0	0	1	0	44
19			1	1	0	1	1	0	1	0	0	1	1	1	0	61
20			0	1	0	1	0	0	0	1	1	1	0	0	1	42
21			0	1	0	1	0	1	1	1	1	0	0	0	0	46
22			1	0	0	0	0	1	1	0	1	1	1	0	0	54
23			1	1	0	0	1	0	0	0	0	1	1	0	1	41
24			1	0	0	0	1	1	0	0	1	0	0	0	0	31
25			0	1	1	0	0	1	0	0	0	0	1	1	0	40
26			0	0	0	0	1	1	0	0	1	0	1	1	1	49
27			1	1	0	0	1	0	0	1	1	0	0	1	0	44
28			1	1	1	1	0	1	0	0	1	0	0	0	0	41
29			0	1	1	0	1	0	0	1	1	1	1	0	1	60
30			0	1	1	0	0	1	1	1	1	1	1	1	1	83
31			0	1	1	1	1	0	1	0	1	0	0	0	0	41
32			0	0	1	1	1	0	0	1	1	0	0	1	0	46
33			0	1	0	0	1	1	1	0	0	1	0	1	1	54
34			0	1	1	1	1	0	1	1	0	0	1	0	1	56
35			1	0	0	1	1	1	1	0	1	1	0	1	1	70
36			1	0	0	0	1	1	0	1	0	1	0	0	1	46
37			0	1	0	0	1	1	0	1	0	1	1	1	0	58
38			1	1	1	1	0	0	0	0	0	0	0	1	1	38
39			1	0	1	1	0	1	0	1	0	0	1	0	1	51
40			1	1	0	0	1	1	1	1	0	0	0	1	0	54
41			0	0	0	0	0	1	1	1	0	1	1	1	0	57
42			1	1	0	0	0	1	0	0	1	0	0	0	1	34
43			1	0	0	1	0	1	1	0	1	0	1	0	0	49
44			1	1	1	0	1	0	1	1	1	1	0	0	1	67
45			1	0	0	1	1	1	1	1	0	0	1	1	1	70
46			1	1	0	0	0	1	0	1	1	0	0	1	0	48
47			0	1	1	0	1	0	0	1	0	0	1	0	1	41
48			0	1	1	1	1	0	0	0	1	0	0	0	0	31
49			1	0	1	0	1	0	1	1	1	1	1	1	1	82
50			0	1	1	1	1	1	0	0	0	1	1	0	0	51
51			1	1	0	1	1	0	1	0	1	0	1	0	0	50
52			1	1	0	0	1	1	1	1	1	0	1	0	1	69
53			0	1	0	1	1	0	1	1	1	0	0	0	0	43
54			1	1	0	1	1	0	1	0	0	0	0	0	0	31
55			1	1	1	1	0	1	1	0	1	1	1	0	0	70
56			1	1	1	0	1	0	1	1	0	1	1	0	1	67
57			1	0	0	1	1	1	0	0	1	0	0	0	1	41
58			0	1	0	0	1	0	0	1	1	0	1	0	0	38
59			1	0	1	1	0	1	0	1	1	1	1	0	1	70
60			1	1	1	1	1	0	0	1	0	0	0	0	0	37
61			1	0	1	1	1	0	1	1	1	1	0	0	1	67
62			1	0	1	0	1	0	0	0	1	0	1	0	0	38
63			0	0	0	1	1	1	0	0	1	0	0	0	1	34
64			1	1	1	1	1	1	1	0	0	0	1	1	0	67
65			0	0	1	0	1	0	0	1	1	1	0	1	1	56
66			1	1	1	1	0	0	0	1	0	0	1	1	0	51
67			0	0	0	0	0	0	0	1	0	0	1	0	0	19
68			1	0	1	1	0	0	0	1	1	0	1	1	0	56
69			1	0	1	0	1	1	0	1	0	0	0	1	0	47
70			0	0	1	0	0	0	0	0	0	1	1	0	1	32
71			0	1	0	1	1	1	0	1	0	1	1	1	1	68
72			1	1	1	1	0	0	0	0	0	1	0	1	1	48
73			0	1	0	0	1	1	0	1	1	0	1	0	1	52
74			1	1	1	1	0	1	0	1	1	0	1	0	1	65
75			1	1	1	0	0	0	1	0	1	0	1	0	0	46
76			1	0	1	0	0	1	1	0	0	1	1	0	0	52
77			0	1	0	0	1	1	1	0	1	1	0	0	0	48
78			0	1	1	0	1	0	1	0	1	0	1	1	0	56
79			1	0	1	0	1	1	1	1	1	0	0	1	1	72
80			1	1	1	1	1	1	1	0	1	0	0	1	0	66
81			1	0	1	1	0	0	1	0	0	0	1	0	1	43
82			1	0	1	0	1	1	0	1	1	0	0	1	0	56
83			1	0	1	1	1	1	0	1	0	1	0	0	0	52
84			1	1	0	0	0	0	0	1	1	1	1	1	0	58
85			1	1	0	1	0	1	0	0	1	1	0	1	0	54
86			1	0	0	1	1	0	1	0	1	0	0	1	0	46
87			1	1	0	0	0	1	1	1	0	0	1	0	1	54
88			1	0	1	0	1	0	1	1	0	0	1	1	0	58
89			0	1	1	0	1	0	1	1	1	0	1	0	0	55
90			0	0	0	1	0	0	1	0	1	1	0	1	1	49
91			1	1	1	0	0	1	0	0	0	0	1	0	1	42
92			1	1	0	1	1	0	0	0	1	1	1	0	0	50
93			1	1	1	1	0	1	0	0	0	0	0	1	0	42
94			1	1	1	0	0	1	1	1	0	0	1	1	0	65
95			1	1	1	0	1	1	0	1	1	0	1	1	0	70
96			0	0	1	1	1	1	1	1	1	1	1	0	1	80
97			0	1	1	0	1	1	0	0	0	1	0	1	1	51
98			1	1	0	1	1	1	1	0	1	1	0	1	0	69
99			0	0	0	1	0	0	1	0	0	0	1	1	0	34
100			1	1	1	0	0	1	0	0	1	0	0	0	1	41
101			0	1	1	1	0	0	0	0	1	0	0	1	0	35
102			1	1	0	0	1	0	1	1	0	1	1	1	0	65
103			1	0	1	0	1	0	0	1	1	0	0	0	0	37
104			1	0	1	1	1	1	0	0	0	1	1	1	1	68
105			0	0	0	0	0	0	1	0	1	1	0	0	1	34
106			0	0	0	1	1	1	1	0	1	1	1	0	0	58
107			1	1	0	1	1	1	1	1	0	0	0	0	0	49

Opções de resposta
0
1

108			0	0	1	1	1	1	1	0	1	1	1	0	1	71
109			1	0	0	1	0	0	0	0	1	1	1	1	0	50
110			1	1	1	1	1	0	0	0	0	1	1	0	0	48
111			1	0	0	1	1	1	0	0	0	1	1	1	0	56
112			0	1	0	1	1	0	1	1	1	1	1	0	1	68
113			1	1	0	0	1	0	0	0	1	1	0	0	1	41
114			1	0	1	1	1	1	1	1	1	0	0	0	1	67
115			1	1	1	1	0	1	0	1	1	1	0	0	1	65
116			1	1	0	0	1	0	0	1	1	0	0	1	1	50
117			1	1	1	1	1	0	1	0	1	1	1	1	0	77
118			1	1	1	1	0	1	0	1	0	1	1	0	0	60
119			0	1	0	0	1	0	1	0	1	0	0	0	0	29
120			1	0	1	0	0	1	0	0	1	0	1	1	0	51
121			0	0	1	1	1	0	1	1	1	0	1	1	0	65
122			0	1	1	1	1	0	0	0	0	0	1	1	0	41
123			1	0	0	1	1	0	0	0	1	1	1	0	0	46
124			0	1	1	0	1	1	1	1	0	1	0	1	0	65
125			1	1	0	1	1	1	0	1	1	1	0	1	1	74
126			0	1	1	1	0	0	0	0	1	1	0	1	0	45
127			0	0	1	1	0	0	0	0	1	1	0	1	1	46
128			1	0	1	0	0	1	1	1	0	1	1	0	1	66
129			0	1	1	0	1	1	1	0	0	0	1	0	0	46
130			1	1	0	0	0	0	1	0	0	1	1	0	0	40
131			1	1	1	1	1	1	0	0	0	1	0	1	0	57
132			1	0	1	0	0	1	0	1	1	1	0	0	1	56
133			1	0	0	0	0	1	1	1	1	1	0	0	0	54
134			0	1	0	1	0	0	0	1	0	0	0	1	1	33
135			0	0	1	1	1	1	0	0	1	1	0	1	0	56
136			0	1	0	0	1	0	1	1	1	0	1	1	1	63
137			1	1	1	0	0	1	0	0	0	1	1	1	1	62
138			0	1	1	0	0	1	0	0	0	1	0	0	0	30
139			0	1	0	1	0	0	0	0	0	1	0	1	0	29
140			0	0	1	0	1	1	0	1	1	0	0	1	0	50
141			1	1	1	0	0	0	0	1	0	0	1	0	0	36
142			0	0	0	0	1	1	0	1	0	1	0	1	1	49
143			0	0	1	0	0	0	0	1	1	1	1	0	1	50
144			0	0	0	1	0	1	0	1	1	0	0	1	1	47
145			1	1	0	1	1	1	0	1	1	1	0	1	1	74
146			0	1	0	0	1	1	0	0	1	1	0	1	1	54
147			1	0	0	1	0	0	1	1	0	1	0	1	0	50
148			0	0	0	0	0	0	1	1	0	0	1	0	0	28
149			1	0	0	1	1	1	0	0	1	1	1	1	0	65
150			0	0	0	1	0	0	1	1	1	1	1	1	0	62
151			1	0	1	0	0	0	0	0	1	1	1	0	1	47
152			1	1	0	1	0	1	0	1	0	1	0	1	1	59
153			1	0	1	1	1	0	1	1	1	0	0	1	0	62
154			0	1	1	0	1	0	1	1	1	0	1	1	0	65
155			0	1	0	0	0	0	1	1	0	0	0	0	0	23
156			0	0	1	0	1	1	1	0	1	1	0	1	1	66
157			0	0	0	1	0	0	0	0	1	0	1	0	1	29
158			0	1	0	1	0	1	0	1	0	1	0	1	0	47
159			1	0	1	1	0	1	0	1	1	1	0	0	0	55
160			0	1	0	1	0	1	0	0	0	1	1	1	0	48
161			1	0	0	0	0	0	1	1	0	1	1	1	1	60
162			0	1	0	1	1	0	1	1	0	0	0	0	1	39
163			1	1	0	1	0	0	1	1	1	0	1	0	1	58
164			1	1	1	1	1	1	0	1	0	0	1	1	1	71
165			0	0	0	0	0	0	1	0	1	1	1	1	0	49
166			0	1	0	0	0	0	0	0	0	0	0	0	1	10
167			0	1	1	0	0	0	1	1	0	1	0	0	0	40
168			0	0	1	0	1	1	1	1	1	0	0	0	1	55
169			0	1	0	0	1	0	1	1	0	0	0	0	0	29
170			1	0	0	0	1	1	0	0	1	1	0	1	0	50
171			1	0	1	0	0	0	0	0	0	1	1	1	1	48
172			1	1	1	0	0	1	0	1	0	1	0	0	0	46
173			1	1	0	0	1	0	1	0	1	1	0	0	1	51
174			1	1	0	0	0	1	0	1	0	1	1	1	0	58
175			1	0	1	1	0	1	0	0	0	0	1	1	0	47
176			0	0	1	1	1	0	0	0	0	0	1	0	1	33
177			1	0	0	0	0	1	0	1	1	1	1	1	0	63
178			1	1	0	1	0	1	1	1	1	1	0	1	0	72
179			1	1	0	0	0	1	0	0	0	0	1	1	0	39
180			0	1	1	1	1	1	1	1	1	1	1	1	1	94
181			1	0	0	1	1	0	1	0	0	1	0	0	0	37
182			1	1	0	0	0	0	1	1	1	0	0	0	0	39
183			0	1	0	1	1	1	1	1	0	0	1	0	1	58
184			0	0	1	0	0	0	0	0	0	1	0	0	0	17
185			1	0	0	1	0	1	1	0	1	1	1	1	0	69
186			0	0	0	0	0	0	1	1	0	1	1	1	1	54
187			1	1	0	1	0	1	0	0	1	1	0	0	0	44
188			1	0	1	1	1	0	0	1	0	1	1	0	0	52
189			1	1	0	1	1	1	1	1	0	1	1	1	1	84
190			1	0	0	1	1	0	0	0	1	1	0	0	1	41
191			0	1	0	1	0	1	0	1	1	0	0	0	0	36
192			0	0	1	0	1	0	1	0	1	1	0	1	0	52
193			1	0	0	0	0	0	1	1	0	1	1	0	0	45
194			0	1	0	0	0	0	1	1	0	1	0	0	0	33
195			1	1	0	0	1	1	0	1	1	1	0	1	0	64
196			0	0	0	0	0	0	1	1	0	0	0	0	0	19
197			0	1	1	0	0	0	0	0	1	1	1	1	0	50
198			0	1	1	0	0	1	0	0	0	0	0	0	0	20
199			1	1	1	1	1	1	1	1	1	1	0	1	0	85
200			0	0	1	1	1	1	0	1	0	0	1	1	0	55
201			1	0	0	0	1	1	1	0	1	0	0	0	1	46
202			1	1	1	1	0	0	1	0	1	0	0	1	0	51
203			0	0	1	1	1	1	1	1	0	1	1	1	0	75
204			1	0	1	0	0	1	0	0	1	0	1	0	1	47
205			0	0	0	0	0	1	1	0	1	0	1	1	0	48
206			1	0	0	0	0	1	1	1	1	1	0	0	1	59
207			1	0	0	1	1	1	1	1	1	1	0	1	0	74
208			1	1	0	0	0	1	1	1	1	1	0	1	0	68
209			0	1	1	1	1	1	1	1	0	0	1	0	0	59
210			0	1	1	1	0	0	1	1	0	1	1	1	0	64
211			1	1	1	1	1	0	0	0	0	1	0	1	1	53
212			0	1	1	1	1	0	0	0	0	0	1	1	1	47
213			1	1	0	0	1	1	1	0	1	0	0	0	1	50
214			1	0	0	0	1	1	0	1	1	1	0	0	1	55
215			0	0	0	0	1	1	0	0	0	0	0	0	0	15
216			0	1	1	1	1	1	0	1	0	1	1	1	0	69
217			0	0	0	1	0	1	0	0	1	1	1	1	1	58
218			1	0	1	0	1	1	1	0	0	1	0	0	1	54
219			1	0	0	0	1	1	0	1	0	0	1	0	0	40
220			1	1	0	0	0	0	0	0	1	1	0	0	0	30
221			1	0	0	1	1	1	0	1	0	1	0	0	1	50
222			0	0	0	0	1	1	1	1	0	1	1	0	1	59
223			1	1	0	0	0	1	0	0	0	1	1	0	0	39
224			1	1	0	1	0	1	1	1	1	1	0	1	1	78
225			0	1	0	0	1	1	1	1	1	1	1	1	0	77

226			1	0	1	1	0	1	1	0	1	1	1	0	0	66
227			1	1	0	0	0	1	1	1	0	0	1	1	0	58
228			1	1	0	1	0	1	0	0	1	0	0	1	1	49
229			0	0	0	0	1	0	0	0	1	0	1	1	1	40
230			1	1	0	1	1	1	1	0	0	1	1	0	0	60
231			0	0	1	1	0	0	0	1	1	1	1	0	1	55
232			1	1	1	1	1	0	1	1	0	0	0	1	1	62
233			0	0	0	1	0	0	1	0	1	0	0	0	0	24
234			1	1	0	1	0	1	0	1	1	0	0	1	1	58
235			0	0	1	0	1	1	1	1	1	1	1	1	0	79
236			1	1	0	1	1	1	0	0	0	1	0	0	0	40
237			1	0	1	1	1	0	0	1	0	0	0	0	0	33
238			0	0	1	0	1	1	0	1	0	0	1	1	0	50
239			1	1	0	1	1	0	0	1	1	1	0	1	0	59
240			1	1	0	0	0	0	1	1	1	0	0	0	1	44
241			1	1	1	0	1	1	0	0	0	1	0	0	0	43
242			0	0	0	0	0	1	0	1	1	1	1	0	0	47
243			0	0	0	1	1	1	1	0	1	1	0	1	1	64
244			0	0	0	0	0	1	0	1	1	0	0	1	1	43
245			0	1	0	1	0	1	1	1	1	1	1	1	1	81
246			1	0	0	1	0	1	1	1	0	1	0	1	1	64
247			1	1	0	1	0	1	1	1	0	1	0	1	1	69
248			0	0	0	1	0	1	1	0	0	0	0	0	0	24
249			0	0	0	0	0	0	0	1	1	0	0	1	1	33
250			0	0	1	1	0	0	0	0	0	1	0	1	0	32
251			1	1	0	1	0	1	1	0	0	0	1	1	0	54
252			1	1	1	0	0	0	0	0	0	1	1	1	0	47
253			1	0	1	0	1	0	1	0	1	0	0	0	1	44
254			0	1	1	0	0	1	1	1	0	1	1	0	1	64
255			1	1	0	0	1	1	1	0	1	0	0	1	0	55
256			0	1	1	1	1	0	0	1	0	1	0	0	1	46
257			0	0	0	0	0	1	1	1	0	1	0	0	0	38
258			0	0	1	1	1	0	1	0	1	0	0	1	1	52
259			1	1	0	0	0	1	0	0	0	0	1	0	0	29
260			1	1	0	1	1	0	0	0	0	0	1	0	1	36
261			1	1	0	1	0	1	1	0	0	0	0	1	0	44
262			0	0	1	0	1	0	1	1	0	1	0	1	1	57
263			0	0	0	0	0	0	0	1	1	0	0	0	0	18
264			1	1	1	1	0	1	1	0	1	1	0	0	0	61
265			0	1	0	1	0	1	0	0	0	1	1	1	1	53
266			1	1	0	0	1	1	0	0	1	1	0	1	0	55
267			0	0	1	1	1	1	1	0	0	1	1	1	1	71
268			1	1	0	1	1	0	1	0	1	1	1	0	1	65
269			1	1	1	1	0	1	0	0	1	0	1	1	1	66
270			1	1	1	1	0	1	0	1	1	0	1	0	1	65
271			1	1	0	0	0	1	0	0	0	0	0	0	1	25
272			0	0	0	1	0	1	0	1	1	1	0	0	0	42
273			0	0	0	1	1	1	1	0	0	0	1	1	0	49
274			0	0	1	0	1	1	0	0	0	1	0	1	0	33
275			1	1	0	1	1	0	1	0	1	1	1	0	0	60
276			1	1	0	0	0	1	0	1	0	1	0	1	1	54
277			1	1	1	0	1	0	1	0	1	1	0	0	1	58
278			0	1	1	0	0	1	0	1	0	0	0	0	1	35
279			0	0	0	0	1	1	1	0	0	0	0	1	0	35
280			0	1	1	0	0	0	0	0	0	0	0	0	0	11
281			0	0	0	0	1	1	1	1	0	1	1	0	1	59
282			1	0	0	0	0	1	1	1	0	1	0	0	0	44
283			1	0	0	0	0	1	1	0	0	0	0	1	0	35
284			1	0	0	1	0	1	1	1	0	1	0	1	1	64
285			1	0	1	1	1	0	1	0	0	0	1	1	1	59
286			0	0	1	1	0	1	1	0	1	0	0	1	1	55
287			1	0	0	1	1	1	1	0	1	1	0	0	0	55
288			0	0	0	0	0	0	0	1	1	0	1	0	1	33
289			0	1	0	1	0	1	1	1	0	1	0	1	0	57
290			0	0	0	1	1	0	1	0	1	0	0	0	1	35
291			0	1	1	1	0	0	0	1	0	0	0	0	0	25
292			1	0	0	0	0	1	0	1	1	1	0	1	0	54
293			0	0	1	1	1	0	1	1	0	0	1	1	0	56
294			1	0	1	0	0	0	0	0	1	0	1	0	0	32
295			0	1	1	1	0	1	1	1	1	1	0	0	1	68
296			1	1	1	0	0	0	0	0	0	0	0	1	1	33
297			1	1	1	0	0	1	1	0	1	0	0	0	0	46
298			1	0	0	0	1	0	0	1	1	1	1	1	1	65
299			1	0	0	1	1	1	0	0	0	1	1	0	1	51
300			1	0	1	0	1	0	1	0	0	0	1	1	1	54
301			0	0	1	0	1	1	0	1	0	0	1	0	0	41
302			0	0	0	1	1	1	1	1	0	0	1	0	0	48
303			1	0	0	0	1	1	0	1	1	0	0	0	1	45
304			1	0	1	1	1	0	1	1	0	0	1	0	1	58
305			0	0	0	0	1	1	1	0	1	1	0	0	0	44
306			0	0	0	1	0	0	1	0	0	1	1	0	0	34
307			0	1	0	1	0	1	1	1	0	0	0	0	0	37
308			1	0	1	1	0	0	0	0	0	1	0	0	0	28
309			0	0	0	0	0	0	0	0	0	0	0	0	0	0
310			0	0	1	1	1	0	0	0	1	0	0	1	0	37
311			0	0	0	1	1	1	1	0	1	1	1	0	0	58
312			1	1	1	0	0	1	1	1	1	1	1	1	1	90
313			0	0	0	0	1	0	0	1	0	1	1	1	0	44
314			0	1	1	0	1	0	1	1	0	1	1	1	1	71
315			0	0	1	0	1	1	0	1	1	1	0	0	0	50
316			1	1	1	1	0	1	1	0	1	1	0	1	0	71
317			0	1	0	1	0	1	0	0	0	1	0	0	1	33
318			1	1	0	0	1	1	1	1	0	0	1	1	1	69
319			0	0	0	1	0	0	0	1	0	1	1	0	1	39
320			0	1	0	1	1	0	1	1	1	1	0	1	1	68
321			1	1	0	0	0	0	1	1	0	1	0	0	1	45
322			1	1	1	1	0	0	0	1	1	1	1	1	0	70
323			1	1	1	0	1	1	0	0	0	0	0	0	0	33
324			0	1	0	1	0	1	0	1	1	1	0	0	0	46
325			1	0	1	0	1	0	0	1	1	1	0	1	0	57
326			1	0	0	1	1	1	0	1	0	1	1	0	1	60
327			1	1	1	0	0	0	1	0	1	0	0	1	1	52
328			1	0	1	0	0	1	0	0	0	0	1	1	1	47
329			1	0	0	1	1	0	1	0	1	0	1	1	0	56
330			1	1	0	1	0	1	0	1	1	0	0	0	0	43
331			0	1	0	1	1	0	0	0	1	0	1	1	0	44
332			0	1	1	0	1	1	0	0	0	1	0	0	0	36
333			0	0	0	1	1	0	1	0	1	0	1	0	1	45
334			0	0	0	1	0	1	1	1	1	0	1	1	0	62
335			0	0	0	0	1	1	0	0	1	0	0	1	1	39
336			0	1	1	0	1	0	1	1	1	0	1	1	0	65
337			0	1	0	0	0	0	1	0	1	0	1	1	0	43
338			1	0	0	0	1	0	0	1	0	0	0	1	0	31
339			1	1	1	0	0	0	0	0	1	0	1	1	1	52
340			1	0	1	0	1	1	1	0	1	1	0	1	1	73
341			0	1	1	1	0	1	1	1	0	0	1	0	0	54
342			0	1	1	0	0	1	0	0	0	0	0	1	1	36
343			0	0	1	1	1	0	0	0	0	1	1	0	1	42

344			0	1	0	0	0	0	1	1	1	0	0	1	1	47
345			0	1	0	0	1	0	0	0	1	0	0	1	0	29
346			0	0	0	0	0	1	1	1	0	0	1	1	1	53
347			0	0	0	1	1	0	0	0	1	1	1	0	1	45
348			0	0	0	0	1	1	0	0	1	1	1	0	0	44
349			0	1	1	0	0	1	0	1	1	1	1	0	1	63
350			1	0	0	1	0	0	1	1	1	0	1	0	0	49
351			1	0	1	0	1	0	1	0	0	1	0	0	0	39
352			1	0	0	0	0	0	1	1	0	0	0	1	0	35
353			0	0	0	0	0	1	1	0	0	0	1	0	1	34
354			1	0	1	1	0	1	1	1	0	0	0	0	0	46
355			1	0	0	1	0	0	1	0	1	1	1	1	0	60
356			1	1	1	1	1	0	0	0	0	0	1	0	1	43
357			0	1	1	1	1	1	0	1	0	1	0	0	1	55
358			0	1	0	1	0	1	0	1	0	1	0	1	0	47
359			0	1	0	1	1	0	1	1	1	0	1	0	1	58
360			0	0	0	1	0	0	0	1	0	1	1	0	1	39
361			1	1	0	1	1	0	0	0	0	1	0	1	0	41
362			1	1	1	1	1	0	0	1	0	0	1	1	0	57
363			1	1	1	0	1	0	1	0	0	0	0	1	0	43
364			1	0	0	0	0	1	1	0	0	1	0	0	0	35
365			1	1	0	1	1	1	0	1	1	0	1	0	1	63
366			1	0	0	1	1	1	1	1	1	1	1	1	0	84
367			1	0	1	0	1	1	1	1	0	0	0	0	0	47
368			1	1	0	0	1	0	0	0	1	1	1	1	0	55
369			1	1	0	0	0	1	0	0	0	0	0	0	1	25
370			1	0	1	0	0	1	1	0	1	0	0	0	1	47
371				1	1	1	1	0	1	0	1	1	1	1	0	70
372				1	0	1	1	1	0	0	1	0	1	1	0	53
373				0	0	1	1	0	1	0	0	1	1	1	0	50
374				1	0	1	1	1	0	0	0	0	1	1	1	49
375				1	0	0	0	0	0	1	0	1	0	1	1	38
376				0	1	0	1	0	0	1	0	0	1	0	1	37
377				0	1	0	0	0	1	1	1	0	1	0	0	45
378				0	0	1	0	0	0	1	1	0	0	1	0	33
379				1	1	1	1	1	1	0	0	1	0	1	1	66
380				1	0	0	1	0	1	1	1	1	1	0	0	58
381				0	1	1	1	1	1	1	0	1	0	0	1	61
382				0	0	0	1	0	1	0	0	1	1	1	1	50
383				1	1	0	1	0	1	1	0	1	1	1	0	65
384				0	1	1	1	0	1	0	1	1	1	1	0	66
385				0	0	0	1	0	0	0	1	0	0	0	0	15
386				0	0	1	1	0	0	1	1	1	0	1	0	49
387				0	1	1	1	0	1	1	0	0	1	1	0	56
388				0	0	0	0	0	0	1	0	1	1	1	1	44
389				1	0	0	1	1	0	1	0	0	1	1	0	48
390				0	1	1	0	1	0	0	1	1	0	1	1	55
391				1	1	0	0	1	1	0	0	1	0	0	1	46
392				1	1	0	1	1	0	0	1	1	0	1	1	61
393				1	1	0	1	0	1	0	0	0	0	0	1	32
394				0	1	1	1	1	0	0	1	1	0	1	0	56
395				0	1	0	1	1	1	1	0	0	1	1	1	66
396				0	0	0	1	1	0	0	0	0	1	1	0	35
397				1	0	0	0	0	0	1	1	0	1	1	0	42
398				0	0	0	1	0	0	1	0	0	0	1	1	30
399				0	0	0	0	1	0	0	1	0	0	1	0	28
400				1	1	1	1	0	1	1	0	0	0	1	0	51
401				0	1	1	1	1	1	0	0	0	1	0	0	46
402				0	1	1	1	1	1	0	0	1	0	0	1	52
403				0	0	1	0	0	0	0	1	1	1	1	1	49
404				0	1	1	1	1	1	0	1	1	0	0	0	56
405				0	0	1	1	1	0	0	0	0	0	1	1	35
406				1	1	0	1	0	0	1	0	1	0	0	0	36
407				1	1	1	0	1	1	1	1	0	0	1	1	68
408				1	1	0	1	0	0	1	1	1	1	1	1	70
409				1	1	0	1	0	0	0	1	0	1	1	1	51
410				1	0	1	0	0	0	0	1	0	0	1	0	28
411				0	0	1	0	1	0	0	1	0	1	0	0	33
412				0	0	1	1	0	1	1	1	1	0	1	1	64
413				0	0	0	0	1	1	0	0	0	0	0	0	19
414				0	1	1	0	0	1	1	1	1	1	0	0	59
415				0	1	1	1	1	0	1	0	0	1	1	1	61
416				0	1	1	0	1	0	1	0	1	0	1	0	50
417				1	0	1	1	1	1	0	1	1	1	1	1	78
418				1	1	0	0	1	1	0	1	0	0	0	1	45
419				1	0	1	0	0	0	1	1	1	1	0	0	47
420				0	1	1	0	0	1	0	1	1	1	0	1	56
421				0	1	0	1	1	0	1	1	0	1	0	0	50
422				1	0	0	1	1	1	0	1	1	1	1	1	73
423				1	0	1	0	1	1	1	1	1	1	0	1	71
424				1	0	1	1	0	1	1	1	1	0	1	0	63
425				0	0	1	1	0	0	1	1	0	1	1	1	54
426				0	0	0	1	0	0	1	0	0	1	1	1	40
427				0	1	0	0	0	0	0	1	1	1	0	0	36
428				0	1	1	0	0	0	0	0	0	1	1	1	37
429				1	1	0	0	0	1	0	0	0	0	0	1	26
430				0	0	0	0	0	0	0	0	0	0	0	1	5
431				1	0	0	1	0	0	1	1	0	1	1	0	48
432				1	0	0	0	1	1	1	1	0	1	0	1	56
433				1	1	1	1	1	1	1	1	1	1	1	1	94
434				1	1	0	0	0	1	0	0	0	0	1	1	36
435				1	1	1	0	0	1	1	0	1	1	0	1	60
436				1	0	0	0	1	1	0	0	0	1	0	1	38
437				1	1	1	0	1	0	0	0	1	0	0	1	40
438				1	1	0	0	0	0	1	1	1	0	0	1	45
439				1	1	0	1	1	1	0	0	1	0	0	0	46
440				1	1	1	0	0	1	0	0	1	1	1	0	55
441				0	1	0	1	1	1	1	1	0	0	1	0	60
442				1	1	0	1	1	0	1	0	1	0	0	1	50
443				1	0	1	0	1	0	0	0	1	1	0	0	38
444				0	0	1	1	1	0	0	1	0	0	0	0	29
445				1	0	1	0	0	0	1	1	1	0	0	1	42
446				0	1	0	0	0	1	1	0	1	0	1	0	46
447				1	0	1	1	1	0	0	1	1	0	1	1	58
448				0	1	1	0	1	1	1	1	1	1	0	0	69
449				1	0	0	0	0	1	0	0	0	0	1	0	24
450				0	0	1	0	1	0	1	0	1	0	0	0	33
451				1	1	1	1	1	0	0	0	0	0	0	1	36
452				1	0	0	1	0	0	1	0	1	1	0	1	44
453				0	0	1	0	1	0	1	0	0	1	1	1	48
454				0	0	0	1	1	0	0	0	0	0	1	0	25
455				0	1	0	0	0	0	0	1	0	1	1	0	36
456				0	0	0	1	0	1	1	1	0	0	1	1	49
457				1	0	1	0	1	1	0	1	0	0	1	1	53
458				1	0	0	0	0	0	1	0	0	1	1	1	38
459				0	1	1	1	0	0	0	1	1	0	1	0	47
460				1	1	1	1	1	1	0	0	1	0	0	0	51
461				0	0	1	0	0	1	1	1	0	1	1	1	58

462				0	1	1	1	0	1	0	1	0	1	0	0	46
463				0	1	0	1	1	1	0	0	0	0	0	1	37
464				1	0	0	1	1	1	0	0	1	0	0	0	39
465				0	0	1	0	0	1	1	1	1	1	0	1	58
466				0	0	1	0	0	1	1	0	1	1	1	1	58
467				0	0	1	1	1	1	1	0	0	1	1	0	58
468				1	1	1	1	1	1	1	1	1	1	1	0	88
469				1	1	0	0	0	1	1	0	1	0	1	0	50
470				0	1	1	0	0	0	0	1	1	1	0	0	41
471				0	1	1	0	0	1	0	0	1	0	0	0	32
472				0	1	0	0	1	0	0	0	1	1	0	1	41
473				0	1	0	1	1	1	1	1	1	1	1	1	85
474				1	1	1	1	1	0	0	1	0	1	0	1	55
475				0	1	1	1	0	1	0	1	1	0	0	1	52
476				1	1	0	0	1	0	0	1	0	1	1	1	54
477				0	0	0	1	1	0	0	1	0	1	0	1	39
478				1	1	0	1	0	1	0	1	1	1	1	0	66
479				1	0	0	0	0	0	0	1	0	0	1	0	23
480				1	0	0	0	1	0	1	1	1	1	0	0	51
481				0	1	0	0	0	1	1	1	1	0	1	1	60
482				0	0	0	0	1	1	1	1	1	1	0	1	62
483				1	1	1	0	0	0	1	0	0	1	1	0	44
484				1	0	0	0	0	0	1	0	1	1	1	1	48
485				0	1	1	1	1	1	0	0	0	0	0	0	37
486				1	0	1	1	0	0	0	0	1	0	0	1	30
487				0	0	1	1	1	0	0	0	1	0	1	1	45
488				1	1	1	1	0	1	0	0	0	1	0	0	41
489				1	1	1	0	1	1	1	0	1	0	1	1	69
490				0	1	0	1	1	0	0	0	1	1	0	0	42
491				1	0	0	0	1	0	0	1	0	0	0	0	23
492				1	1	0	0	0	0	1	0	0	0	0	1	25
493				1	0	0	1	0	1	0	0	0	0	1	0	30
494				1	0	0	0	1	1	0	1	1	0	0	1	48
495				1	1	1	0	0	0	0	0	0	1	0	0	26
496				0	0	1	0	1	0	1	1	0	1	0	1	47
497				1	0	1	0	0	1	0	0	0	1	1	1	44
498				0	1	1	1	1	0	1	1	1	1	1	0	74
499				0	0	0	1	0	1	0	0	1	1	1	1	50
500				0	0	1	1	0	1	0	1	0	0	0	1	35
501				0	1	0	1	0	0	1	0	0	0	1	1	37
502				0	1	0	1	0	1	0	0	1	0	0	0	33
503				0	1	1	0	0	0	0	0	0	1	1	0	31
504				0	1	0	1	1	1	0	0	1	0	1	1	57
505				1	0	0	0	1	1	0	0	0	1	0	0	33
506				1	0	1	0	1	1	0	1	0	1	1	1	62
507				0	1	1	0	0	1	0	1	0	0	1	0	41
508				0	1	1	1	0	1	1	0	1	1	0	1	61
509				1	1	1	0	1	0	1	1	1	1	0	0	63
510				0	1	0	0	1	0	0	1	0	1	0	1	40
511				0	0	0	1	1	0	0	0	1	1	1	1	50
512				0	1	1	0	1	0	0	0	1	0	0	1	36
513				0	0	0	1	1	1	1	1	0	1	1	1	68
514				0	1	1	1	1	0	1	0	0	1	1	1	61
515				0	1	1	0	1	0	1	1	0	1	0	0	49
516				1	0	1	1	0	0	0	1	1	1	1	0	53
517				0	0	0	1	0	1	0	0	1	0	1	1	41
518				1	0	1	0	1	0	0	0	0	0	1	1	33
519				1	1	0	1	1	1	1	1	1	0	0	0	64
520				0	1	0	1	0	0	1	1	0	1	0	0	41
521				0	1	1	0	0	0	0	0	0	1	1	1	37
522				1	0	1	1	0	1	1	0	0	0	0	1	39
523				1	1	1	1	0	0	1	0	0	0	0	0	31
524				1	1	1	1	1	1	1	0	0	1	1	0	69
525				1	1	0	0	0	0	1	0	0	1	0	0	30
526				0	0	1	1	1	0	1	0	0	1	1	1	54
527				1	0	1	0	0	1	0	0	1	0	1	0	39
528				0	0	1	1	0	0	0	0	1	1	1	0	40
529				0	1	1	0	0	0	0	0	1	0	1	0	32
530				1	0	1	0	0	1	1	0	1	0	1	1	53
531				1	1	0	0	1	0	1	1	0	0	1	1	54
532				0	0	0	1	0	1	1	1	0	1	0	0	43
533				0	1	0	0	0	0	0	0	0	1	1	0	27
534				1	1	1	0	0	1	1	1	1	0	0	0	54
535				0	1	0	0	1	1	0	1	0	1	1	1	60
536				0	1	0	1	1	1	0	1	0	1	1	1	66
537				1	0	0	1	0	1	1	0	1	0	0	0	39
538				0	0	1	1	0	1	1	1	0	1	1	1	63
539				1	1	0	1	1	1	0	0	0	0	0	1	41
540				1	1	1	0	0	1	0	1	1	1	0	1	60
541				0	1	1	1	1	0	1	0	0	1	1	0	55
542				0	0	1	1	1	0	1	1	1	1	0	0	57
543				0	1	1	1	0	0	0	0	0	0	0	0	18
544				1	0	0	1	0	1	0	1	0	1	1	0	49
545				1	0	1	0	0	1	0	0	1	1	1	1	54
546				1	0	1	1	0	0	0	1	0	1	0	0	34
547				0	1	1	1	1	1	0	1	0	0	0	1	51
548				1	0	1	1	0	0	0	1	0	0	1	1	39
549				1	0	0	1	1	0	1	0	1	1	1	0	58
550				1	0	1	1	1	0	0	1	0	0	0	0	33
551				1	1	1	0	1	0	0	1	1	1	0	1	59
552				0	0	1	0	0	0	0	0	0	1	1	0	24
553				0	0	0	0	0	0	0	1	0	1	0	0	19
554				0	1	1	0	0	0	1	1	1	1	0	0	49
555				1	1	1	0	1	1	0	1	0	0	0	0	44
556				0	0	0	1	0	0	1	1	0	0	0	1	29
557				1	0	0	1	1	1	1	0	1	1	1	1	73
558				0	1	1	0	0	1	1	1	0	1	0	1	55
559				1	0	1	0	1	0	0	1	0	0	0	1	33
560				1	1	0	0	0	1	1	1	0	1	0	0	49
561				1	0	0	0	0	1	1	1	1	0	1	1	57
562				0	1	1	0	0	1	0	0	0	0	0	0	22
563				1	1	0	1	1	1	1	0	0	0	0	1	50
564				0	0	0	1	1	1	0	1	1	0	1	1	59
565				1	1	0	0	0	0	1	1	1	0	0	0	39
566				0	0	1	1	1	0	0	0	1	0	1	0	40
567				1	0	0	1	1	1	0	1	1	1	0	0	58
568				1	0	1	0	1	0	1	1	1	0	0	1	52
569				0	1	1	1	1	0	0	1	0	0	1	1	51
570				0	1	1	1	1	0	1	1	1	1	1	1	80
571				0	0	0	1	1	0	0	1	0	1	0	1	39
572				1	1	0	0	0	0	0	1	1	1	0	1	45
573				1	1	1	1	0	0	0	1	1	1	0	1	56
574				1	1	1	1	1	1	0	1	0	0	0	0	50
575				0	1	0	1	0	0	0	1	1	1	1	0	51
576				1	1	0	0	0	1	0	0	1	1	0	0	41
577				1	0	0	1	1	0	0	1	0	1	0	0	38
578				0	0	0	0	0	0	1	1	0	1	0	0	28
579				0	1	1	1	0	1	1	1	1	1	0	1	70

580				1	0	1	1	1	0	0	0	0	0	1	1	39
581				1	1	1	0	0	1	0	0	1	0	0	0	36
582				0	0	1	0	1	0	0	0	0	0	1	0	24
583				1	0	0	1	0	1	0	1	1	1	1	0	59
584				0	1	0	0	1	0	1	1	0	1	1	1	59
585				1	0	0	1	0	1	0	1	1	1	0	0	49
586				1	0	1	0	1	1	1	1	1	0	0	0	56
587				0	0	1	0	1	1	1	1	0	0	0	1	47
588				0	1	0	1	1	0	0	0	1	0	0	1	37
589				0	0	0	1	1	1	0	1	1	1	1	1	69
590				0	1	1	0	1	0	1	0	1	1	0	0	49
591				1	0	1	0	1	1	0	0	0	0	0	1	33
592				0	0	1	0	0	1	1	0	0	1	1	1	48
593				1	1	1	1	1	1	0	0	0	0	1	1	56
594				0	1	1	1	0	0	0	1	0	1	0	0	36
595				1	1	1	1	0	1	0	0	1	1	1	0	61
596				1	0	1	0	1	0	0	1	0	1	1	0	47
597				1	1	0	0	1	1	1	0	1	1	0	0	59
598				0	0	1	0	1	0	1	1	1	0	0	0	42
599				1	0	1	1	0	1	1	1	1	0	1	1	68
600				1	0	0	0	1	1	1	1	0	0	1	1	57
601				0	1	1	0	1	0	0	1	1	1	1	1	65
602				1	1	0	1	1	0	1	1	1	0	1	1	69
603				0	1	1	1	0	0	1	1	1	0	1	0	56
604				1	0	1	0	1	1	1	1	1	1	1	1	81
605				0	0	1	0	0	0	1	1	0	0	0	0	23
606				0	1	0	0	1	1	1	1	0	0	1	1	59
607				0	0	0	0	0	1	1	1	1	0	0	0	38
608				1	1	1	0	0	1	1	1	0	0	1	1	59
609				1	0	0	0	1	1	0	0	0	1	1	1	48
610				0	0	0	0	1	1	1	1	0	1	0	0	47
611				1	1	1	0	0	0	0	0	0	1	0	0	26
612				1	1	1	1	0	1	0	1	0	0	1	1	56
613				0	1	1	0	1	0	0	1	0	1	0	0	40
614				0	0	1	0	1	1	0	1	0	1	0	1	48
615				1	0	0	0	1	0	0	0	0	1	0	1	28
616				0	0	1	1	0	0	0	1	1	0	1	1	45
617				1	1	0	0	1	0	1	0	0	1	0	1	44
618				0	1	1	0	0	1	1	0	0	1	1	1	55
619				1	0	1	0	0	1	0	1	0	1	0	0	38
620				0	0	1	1	0	0	0	1	1	0	0	0	30
621				1	1	1	1	1	1	1	0	0	0	0	0	50
622				0	1	1	1	0	1	0	0	1	1	0	0	47
623				0	1	0	1	0	0	0	1	0	0	1	1	37
624				1	1	0	0	0	1	1	0	0	0	0	1	35
625				0	1	0	0	1	1	1	0	0	1	1	0	55
626				1	0	1	1	1	1	1	0	1	1	0	0	62
627				1	1	0	0	1	1	1	0	0	0	1	1	54
628				1	0	1	1	0	1	1	0	1	0	0	0	44
629				0	0	0	0	1	1	1	0	1	1	0	0	48
630				0	0	1	0	0	0	1	1	1	0	0	0	33
631				0	0	1	0	1	1	0	1	1	0	0	0	43
632				0	1	0	0	1	0	0	0	0	1	0	0	26
633				0	0	0	0	1	0	0	1	1	0	0	0	28
634				1	1	1	1	1	0	0	1	0	1	0	1	55
635				0	1	0	0	0	0	0	1	0	1	0	1	31
636				1	0	0	1	1	0	1	1	0	0	0	1	43
637				0	0	1	1	1	0	1	0	0	0	1	1	44
638				1	0	0	1	0	1	1	1	1	0	0	0	48
639				1	1	0	0	0	1	1	0	1	0	0	1	45
640				0	0	1	0	1	0	0	1	0	1	1	0	43
641				1	0	1	1	1	1	0	1	1	0	1	1	68
642				0	1	0	0	0	1	1	1	0	0	0	1	40
643				1	0	1	1	1	0	1	1	0	1	0	1	57
644				0	1	1	1	1	0	0	1	1	1	1	1	71
645				1	1	1	1	1	0	0	1	1	0	0	1	55
646				0	1	0	0	1	0	0	1	1	0	0	0	35
647				0	0	1	0	0	0	0	0	0	1	1	1	30
648				1	1	0	0	1	0	1	0	0	1	0	0	39
649				1	1	1	1	1	0	1	1	1	1	1	0	79
650				0	0	1	1	1	1	0	1	0	0	0	1	44
651				1	1	1	0	0	0	0	0	1	1	1	1	51
652				0	1	0	0	1	0	0	1	0	0	0	1	31
653				1	1	1	0	0	0	0	1	1	0	1	1	50
654				1	1	0	0	0	1	0	1	0	0	1	0	40
655				1	0	0	0	1	0	1	0	1	0	0	1	38
656				1	1	0	1	1	1	0	1	0	1	0	0	55
657				0	1	0	0	0	1	0	0	0	0	1	0	27
658				1	0	1	1	1	1	1	1	1	1	1	1	87
659				1	0	0	0	1	1	0	1	1	0	1	0	52
660				1	1	0	1	0	0	0	1	0	1	0	0	36
661				1	1	1	0	1	0	1	0	1	0	1	1	59
662				0	0	0	1	0	1	0	1	0	0	1	1	40
663				0	0	0	1	1	1	0	0	0	0	1	1	40
664				1	0	0	1	0	1	1	0	0	1	0	0	38
665				0	0	0	1	0	1	0	0	1	1	1	1	50
666				1	0	1	1	1	0	0	0	1	0	1	1	49
667				0	1	0	0	1	0	1	0	1	1	0	1	50
668				0	0	1	0	0	0	1	0	1	1	0	0	33
669				1	1	1	0	0	0	1	1	0	0	0	0	34
670				1	1	1	1	0	0	1	1	0	0	0	1	45
671				0	0	1	0	0	0	1	0	0	0	0	0	14
672				0	0	0	0	0	1	0	0	1	1	1	0	39
673				0	0	1	0	0	1	0	0	0	1	1	1	40
674				0	1	1	0	1	1	0	1	1	1	1	1	75
675				0	1	0	1	1	1	0	1	1	1	0	1	66
676				0	0	1	0	1	0	0	0	0	0	0	1	19
677				1	1	1	1	1	1	0	0	1	0	1	1	66
678				1	0	1	0	1	1	0	0	0	1	0	1	43
679				0	1	0	1	1	1	1	0	0	1	0	1	56
680				0	1	0	1	1	1	0	0	1	0	1	0	52
681				1	1	0	1	0	1	0	0	1	0	0	1	42
682				1	0	1	0	1	0	1	1	0	1	1	1	61
683				1	0	1	0	0	1	1	1	0	0	1	1	52
684				1	0	0	1	1	0	1	0	0	0	0	0	28
685				1	0	0	1	0	0	1	0	0	1	1	0	38
686				1	0	1	1	1	0	1	0	0	0	0	1	38
687				0	0	1	1	1	0	1	0	1	0	0	0	39
688				0	0	1	1	0	1	0	0	0	0	1	0	30
689				1	1	1	0	1	1	1	1	0	1	1	1	78
690				1	1	1	0	1	0	0	0	1	1	0	0	45
691				1	0	0	1	1	1	1	0	0	1	1	1	63
692				0	0	0	1	0	0	1	0	1	1	1	1	49
693				0	1	0	0	0	0	1	1	0	0	1	1	40
694				1	1	0	1	0	0	0	0	0	1	1	1	42
695				1	1	0	0	0	0	1	0	1	1	0	0	40
696				1	1	0	1	1	0	0	0	0	0	1	1	41
697				1	1	1	1	0	1	0	0	1	0	0	1	47

698				0	1	0	1	0	0	1	1	1	1	0	0	50
699				1	0	0	0	0	1	1	0	1	1	1	1	58
700				1	0	1	1	1	0	1	0	0	0	1	1	48
701				0	1	1	0	0	0	1	1	0	1	0	1	45
702				0	0	1	1	0	0	0	1	0	1	0	1	35
703				1	0	1	1	1	0	0	0	0	1	0	0	34
704				1	0	0	0	1	0	0	0	0	0	1	1	29
705				1	1	1	1	1	0	0	1	1	0	1	0	60
706				0	0	0	1	1	1	0	1	0	0	0	0	34
707				0	0	0	1	0	1	0	0	1	0	1	0	36
708				0	0	0	1	0	0	1	0	1	0	1	1	40
709				1	0	0	0	0	1	0	0	1	1	0	0	34
710				1	1	0	0	0	1	1	1	0	1	1	1	64
711				1	0	0	1	0	1	0	1	0	0	0	0	29
712				0	1	1	0	1	1	1	0	1	0	0	0	50
713				1	1	0	1	1	1	1	0	1	0	1	1	70
714				1	0	0	1	1	1	0	0	0	1	1	1	54
715				0	0	0	0	0	1	1	1	0	0	1	1	43
716				1	0	0	1	0	1	1	0	0	0	0	1	34
717				1	1	0	0	0	0	0	1	0	0	0	1	26
718				0	1	1	1	1	0	0	0	0	0	1	0	37
719				0	0	1	1	0	0	1	1	1	0	0	0	39
720				0	1	1	0	1	1	1	1	1	1	0	1	74
721				0	0	0	1	0	1	1	0	1	1	0	0	44
722				1	0	0	1	1	1	1	0	0	1	0	1	53
723				0	0	1	1	1	0	0	1	0	1	0	0	39
724				0	0	0	0	0	0	1	0	1	0	0	1	24
725				1	1	0	0	0	0	0	0	0	0	1	0	21
726				1	1	0	1	1	0	1	1	0	0	1	0	54
727				0	0	1	0	1	0	0	0	0	1	1	0	34
728				1	0	1	0	0	1	0	0	1	0	0	0	29
729				0	1	0	0	1	0	0	0	0	0	0	1	22
730				0	1	1	1	0	1	0	0	1	1	0	0	47
731				1	1	1	0	0	1	0	0	1	0	0	1	41
732				1	0	0	1	0	0	0	0	1	1	0	1	35
733				0	1	1	1	0	0	0	0	1	0	0	0	28
734				0	0	1	0	1	0	0	1	1	0	1	0	43
735				1	0	0	1	1	0	1	1	0	1	0	0	47
736				0	0	1	1	1	0	0	1	1	1	1	1	64
737				1	0	0	0	1	1	1	1	1	1	1	0	71
738				1	1	1	0	0	1	0	1	1	1	1	0	65
739				0	1	1	1	0	1	1	0	0	0	0	1	42
740				1	0	0	0	0	0	1	1	0	0	1	0	32
741				1	1	1	0	1	1	0	1	1	0	0	0	54
742				1	0	0	1	0	1	0	0	1	1	0	1	45
743				1	0	1	1	1	1	0	0	0	0	0	0	34
744				1	1	1	1	0	1	1	1	1	0	1	0	70
745				1	1	1	1	1	1	1	1	1	1	1	1	94
746				1	0	0	1	1	0	1	0	0	1	0	1	43
747				1	1	1	0	0	1	1	1	0	1	1	1	69
748				0	0	1	0	0	1	1	1	1	1	0	0	52
749				0	1	1	1	1	0	0	1	1	0	1	1	61
750				0	0	1	1	0	0	1	1	0	1	0	1	44
751				1	0	0	1	1	1	0	1	1	1	1	1	73
752				0	0	1	0	1	0	0	0	1	0	0	1	29
753				0	1	1	0	0	0	0	0	0	1	1	0	31
754				0	0	0	1	0	0	0	1	0	1	0	1	30
755				1	0	0	0	1	1	1	0	1	1	1	1	67
756				0	0	1	0	1	0	1	1	1	0	0	1	47
757				0	0	1	0	0	1	1	1	1	1	1	1	68
758				1	1	0	0	1	1	0	1	1	1	1	1	74
759				1	0	0	1	1	1	0	0	1	0	1	0	49
760				1	0	0	1	1	1	1	1	0	1	0	1	62
761				1	0	1	1	0	0	0	0	0	1	1	0	34
762				1	1	1	1	0	0	0	0	1	0	0	0	32
763				0	0	1	0	1	1	0	0	1	1	0	1	49
764				1	0	1	0	1	1	1	0	0	1	1	1	62
765				1	1	0	1	1	0	1	1	0	1	1	1	69
766				0	1	0	0	1	0	1	1	1	0	1	0	54
767				0	0	1	0	0	1	1	0	1	0	1	0	43
768				0	0	0	1	0	1	1	0	0	0	1	1	40
769				0	1	1	0	1	0	0	0	0	1	0	1	36
770				1	1	0	1	1	0	0	0	0	1	0	0	36
771				1	1	0	1	1	0	0	1	0	1	1	1	60
772				0	0	0	1	0	1	0	1	0	0	0	0	25
773				1	0	0	1	1	0	1	0	0	0	1	1	43
774				1	1	0	0	1	1	1	0	1	1	1	1	74
775				1	0	1	1	1	1	1	0	0	1	0	0	52
776				1	1	1	1	0	1	1	0	1	0	1	1	66
777				0	1	0	0	1	0	0	1	1	1	1	0	55
778				0	0	1	1	1	0	0	1	1	1	1	0	58
779				1	0	1	0	0	0	0	0	1	1	1	0	38
780				1	1	1	0	0	1	0	0	0	1	0	1	41
781				1	1	0	1	1	0	1	0	1	0	0	1	50
782				0	0	1	1	1	1	0	1	1	0	1	0	59
783				1	0	0	0	1	0	1	0	0	1	0	1	37
784				0	0	1	0	0	0	0	0	1	1	0	0	24
785				0	1	1	1	1	0	1	0	0	1	1	1	61
786				1	0	1	1	0	0	0	1	1	0	0	0	34
787				0	0	0	1	1	1	0	0	0	0	0	1	30
788				1	1	0	0	0	0	0	1	0	0	1	1	36
789				0	1	0	0	1	1	1	1	0	1	0	1	59
790				1	1	1	0	0	0	1	1	0	0	0	0	34
791				1	0	0	1	1	1	1	0	1	0	0	0	48
792				0	1	1	0	1	1	0	0	0	1	1	0	50
793				0	1	1	0	0	0	0	1	1	0	1	1	46
794				1	1	0	1	0	0	0	1	0	0	0	1	32
795				0	1	0	0	0	1	1	0	1	0	1	0	46
796				0	0	0	0	1	0	0	0	1	1	1	1	44
797				0	1	0	1	1	1	0	0	0	1	1	0	51
798				0	1	0	0	1	0	1	1	0	0	1	1	50
799				0	1	1	0	1	0	0	0	1	0	0	0	31
800				0	1	0	1	0	0	1	1	0	1	0	0	41
801				0	1	0	0	0	0	1	0	0	0	0	1	21
802				0	1	1	0	0	0	0	1	0	1	1	0	41
803				0	0	0	1	1	1	1	0	1	0	1	1	59
804				0	1	0	1	1	1	1	0	0	1	0	1	56
805				1	0	1	0	1	1	0	0	0	0	0	0	28
806				1	1	0	0	0	0	0	0	0	1	1	1	36
807				1	1	1	1	1	1	0	1	1	1	0	0	70
808				1	0	1	0	1	1	0	1	0	0	0	1	43
809				1	1	1	1	1	1	1	1	1	0	0	1	74
810				1	0	0	0	0	1	0	1	0	1	0	1	38
811				1	0	1	0	1	0	1	1	0	0	0	0	36
812				0	0	1	0	1	0	0	1	0	1	1	0	43
813				0	1	1	0	0	1	1	1	1	1	1	1	75
814				1	0	1	1	0	0	0	1	0	0	1	1	39
815				1	1	0	0	1	0	0	1	1	0	0	1	45

816				1	1	0	0	1	1	1	0	1	0	1	0	59
817				1	1	0	1	0	0	0	0	0	1	1	1	42
818				1	1	0	0	1	0	0	0	1	1	1	1	55
819				1	0	0	0	1	0	1	1	0	1	0	1	46
820				0	1	1	1	0	1	1	1	0	0	1	1	61
821				0	1	0	0	0	0	1	1	0	1	0	0	35
822				1	0	1	1	0	1	1	1	0	0	0	0	43
823				1	0	1	1	0	0	0	1	1	0	0	1	39
824				1	0	0	1	1	0	1	1	0	1	0	0	47
825				0	0	0	0	0	0	0	1	1	0	1	0	29
826				1	0	0	1	1	0	1	0	0	0	0	1	33
827				1	0	0	0	1	0	0	0	0	1	0	1	28
828				1	1	1	1	1	0	1	1	0	1	1	1	74
829				0	0	1	1	1	1	0	1	0	0	1	1	54
830				0	1	1	1	1	0	1	0	1	0	1	0	56
831				0	0	0	1	1	1	0	1	1	0	0	1	49
832				0	1	0	0	1	1	0	0	0	1	0	1	41
833				1	0	1	1	1	1	1	0	0	0	0	0	43
834				1	0	1	1	0	0	0	1	1	0	1	0	44
835				0	1	1	1	0	0	0	1	0	1	1	0	46
836				0	0	0	1	0	1	0	1	0	1	0	1	40
837				0	0	0	1	0	1	1	0	1	0	0	1	40
838				1	0	1	1	1	0	1	1	1	1	1	1	77
839				1	0	1	1	1	1	1	0	1	0	0	0	53
840				1	0	1	0	1	1	0	0	1	1	0	0	48
841				1	1	1	1	1	1	1	1	0	0	0	1	64
842				0	0	1	1	0	0	0	1	1	1	1	1	55
843				0	0	0	1	0	1	1	0	0	1	0	0	34
844				0	1	0	1	0	1	1	0	0	0	0	1	37
845				1	0	0	1	1	1	0	1	1	0	1	1	63
846				1	0	1	1	0	0	0	1	1	0	0	0	34
847				0	0	1	1	1	0	0	0	0	1	1	0	39
848				1	0	1	1	1	0	0	1	0	0	0	0	33
849				1	0	0	0	0	0	0	0	1	0	1	0	24
850				0	1	1	0	0	0	0	0	0	1	0	1	27
851				1	0	0	0	0	1	0	0	0	0	1	1	29
852				1	1	0	1	1	1	0	1	0	0	1	0	55
853				1	0	1	1	1	0	0	1	1	1	0	0	53
854				1	0	0	0	1	1	1	0	1	1	1	1	67
855				0	0	0	1	1	0	1	0	1	0	1	1	49
856				0	0	1	0	0	1	1	1	0	0	1	1	48
857				1	1	1	1	0	1	0	1	0	1	0	1	56
858				0	0	1	0	0	1	1	0	1	1	1	1	58
859				1	1	0	1	1	1	1	1	1	0	1	1	79
860				1	1	1	0	1	0	0	1	1	0	0	0	44
861				0	0	1	0	0	1	0	0	1	1	1	0	44
862				0	1	1	1	1	1	0	0	0	1	1	1	62
863				1	1	1	0	1	1	0	1	0	1	0	0	54
864				0	1	0	0	1	1	1	1	0	0	0	0	44
865				0	0	1	1	1	0	0	0	0	1	0	1	35
866				0	1	1	1	0	0	0	1	0	0	0	1	32
867				1	0	0	1	0	1	1	0	0	0	0	1	34
868				0	0	0	0	0	1	1	1	1	0	0	0	38
869				1	0	1	1	0	0	0	1	0	1	1	0	44
870				0	0	1	0	1	0	0	0	1	1	1	1	49
871				1	0	0	1	1	1	0	1	0	1	0	0	48
872				0	1	1	1	1	0	0	1	1	0	1	0	56
873				0	0	1	0	1	1	1	1	1	0	0	0	52
874				1	1	1	0	1	1	0	0	0	0	1	1	50
875				1	0	0	0	1	1	0	1	1	0	1	1	58
876				1	1	0	1	1	1	0	1	0	1	0	0	55
877				0	0	0	0	1	1	1	1	0	1	1	1	62
878				0	0	0	1	1	0	0	0	0	1	0	1	30
879				0	1	1	0	1	1	1	0	0	1	1	1	65
880				0	0	1	0	1	0	0	0	1	0	1	0	34
881				0	1	0	1	0	1	1	0	1	0	1	0	51
882				1	1	0	1	0	0	0	1	0	0	1	0	36
883				0	0	1	1	0	1	1	1	1	0	0	1	54
884				0	0	1	0	0	0	1	0	0	0	0	0	14
885				1	1	1	0	1	1	0	0	0	1	1	0	55
886				1	1	1	1	0	1	0	0	1	0	1	1	57
887				0	1	1	1	0	0	1	0	0	0	0	0	27
888				1	0	0	0	1	0	0	0	0	0	0	1	19
889				1	1	0	1	1	1	0	1	0	1	0	1	60
890				1	0	0	1	0	1	0	0	0	0	1	1	35
891				1	0	1	0	1	1	1	0	0	1	0	0	47
892				0	1	0	1	0	1	1	0	0	0	1	1	47
893				1	1	0	0	1	1	0	0	0	1	1	0	50
894				0	1	1	0	1	0	0	0	1	0	1	0	41
895				1	1	1	1	0	1	0	0	1	0	0	1	47
896				1	1	0	1	0	1	1	1	0	0	1	0	55
897				1	0	0	1	0	0	0	0	0	1	0	1	25
898				1	1	1	1	0	1	0	0	1	1	0	1	57
899				1	1	1	0	1	0	0	0	1	0	0	0	35
900				0	0	1	0	0	1	0	1	1	0	1	1	49
901				1	0	1	1	1	1	1	0	1	0	1	1	68
902				0	1	1	1	1	1	0	0	0	1	0	1	52
903				1	1	1	0	0	1	0	1	1	0	0	1	50
904				0	1	1	1	0	1	0	1	1	0	0	0	47
905				0	1	1	1	1	0	1	1	0	0	0	0	45
906				0	1	1	0	1	1	1	0	0	1	0	0	49
907				0	1	1	0	1	0	1	1	1	1	0	1	64
908				0	1	0	0	0	0	0	1	1	0	1	0	36
909				1	0	0	1	1	1	1	0	1	0	0	1	53
910				0	0	0	1	1	0	0	0	1	1	1	1	50
911				0	1	1	0	1	0	0	0	1	1	0	1	46
912				0	1	1	1	1	0	0	0	1	0	0	1	42
913				0	0	1	0	1	0	1	0	0	1	0	1	38
914				0	1	0	1	0	0	0	0	0	0	1	0	23
915				1	0	1	1	1	0	1	0	1	0	1	0	53
916				1	0	1	0	0	0	0	1	0	0	1	1	33
917				0	1	0	0	0	0	0	1	0	1	0	0	26
918				1	1	0	0	0	1	0	1	0	0	0	1	36
919				1	1	0	1	1	1	0	1	1	0	0	1	61
920				1	1	0	0	0	0	0	1	1	0	0	1	36
921				0	0	0	0	0	1	1	1	1	1	0	1	53
922				0	0	0	0	0	1	1	0	0	1	1	0	38
923				1	0	0	1	1	1	0	1	0	1	1	1	63
924				0	0	1	0	0	1	0	1	0	0	1	1	39
925				0	1	0	0	0	1	1	0	1	0	1	0	46
926				1	0	0	0	1	0	0	0	1	1	0	0	33
927				0	1	0	0	0	1	1	1	0	0	1	1	50
928				1	0	1	1	1	1	1	0	1	0	0	1	58
929				0	0	0	1	0	0	0	0	0	0	0	1	11
930				0	0	1	1	1	1	1	0	0	0	1	0	49
931				1	0	0	1	1	0	0	0	1	1	0	1	44
932				0	1	0	0	0	1	1	0	1	1	1	0	55
933				0	0	1	1	0	1	1	1	0	0	0	0	39

934				0	1	1	0	0	1	1	1	0	0	1	0	50
935				0	0	0	1	0	1	0	1	0	1	0	1	40
936				0	0	0	1	1	0	1	0	1	0	1	0	44
937				1	0	0	1	1	0	1	1	0	0	1	0	47
938				1	1	0	1	1	1	1	1	1	0	1	0	74
939				0	1	0	0	1	1	1	0	0	1	1	0	55
940				1	0	0	1	1	0	0	0	1	0	0	0	29
941				0	1	1	0	1	0	0	1	1	1	1	1	65
942				0	1	0	0	1	1	0	1	0	0	0	1	41
943				0	0	0	1	1	1	1	0	0	0	1	0	44
944				0	0	1	0	1	1	0	1	0	1	0	1	48
945				1	1	1	1	1	0	1	0	0	1	0	1	55
946				1	0	1	0	0	0	0	0	1	1	1	0	38
947				1	1	0	1	1	1	1	0	0	0	1	1	60
948				0	1	0	1	1	1	1	1	0	0	1	0	60
949				1	1	1	1	0	1	0	1	0	0	1	0	51
950				1	0	1	1	1	0	1	1	1	1	1	1	77
951				1	0	1	1	1	0	1	1	0	0	1	1	57
952				1	0	1	0	1	1	1	1	0	0	1	0	56
953				0	0	1	0	1	0	1	0	0	1	1	1	48
954				0	0	1	0	1	1	1	0	0	1	0	1	48
955				0	0	0	1	0	0	1	0	0	0	1	1	30
956				1	0	1	1	0	0	0	1	1	0	1	0	44
957				0	0	0	1	0	0	1	0	1	1	0	0	34
958				1	0	1	1	0	0	0	1	1	0	0	0	34
959				1	0	1	1	1	0	0	1	0	0	0	0	33
960				0	1	1	1	0	0	0	1	1	0	0	0	37
961				1	1	1	0	0	1	0	1	0	1	0	0	45
962				1	0	1	1	0	0	1	1	0	1	0	1	48
963				0	1	0	0	1	1	0	1	0	1	1	1	60
964				0	0	0	0	1	1	0	0	0	1	1	0	39
965				1	0	1	1	0	0	1	1	1	1	1	1	68
966				1	1	0	0	1	1	1	0	0	1	0	0	49
967				0	1	0	0	0	1	0	1	0	0	1	0	36
968				0	0	0	0	1	0	1	0	1	0	0	1	33
969				1	1	1	0	1	0	0	1	0	0	0	0	34
970				1	1	1	1	0	1	0	0	1	0	1	1	57
971				1	1	1	1	1	0	1	0	0	0	1	0	50
972				0	0	0	0	1	1	0	1	1	1	1	1	63
973				0	0	0	1	1	1	1	1	1	0	0	1	58
974				0	1	1	0	1	1	1	1	1	0	0	1	64
975				1	1	1	1	0	1	1	1	1	0	1	0	70
976				1	1	0	0	1	1	0	0	1	0	0	0	40
977				1	0	0	0	1	0	0	0	1	0	0	0	23
978				1	1	1	1	0	1	0	0	0	1	0	1	47
979				1	1	1	0	0	1	1	0	1	0	0	0	45
980				0	1	1	0	0	0	1	1	1	1	0	0	49
981				0	1	0	1	1	1	0	1	0	1	0	0	51
982				1	1	1	1	0	0	1	1	1	1	0	0	59
983				1	0	1	0	1	1	0	1	0	1	0	1	52
984				1	1	1	1	0	1	0	1	0	1	1	0	60
985				1	0	1	1	1	0	1	0	1	1	1	0	62
986				0	0	1	1	0	0	1	0	1	1	1	0	49
987				1	0	1	0	1	0	0	1	1	1	1	0	57
988				0	0	0	1	1	1	0	1	0	0	0	0	34
989				1	0	0	0	0	1	1	0	1	0	1	1	48
990				0	1	0	0	1	0	0	0	1	1	1	1	51
991				1	0	1	1	0	0	1	0	0	0	0	1	29
992				0	0	0	1	1	1	0	1	0	1	0	0	44
993				0	1	1	0	1	0	1	1	1	1	1	1	74
994				1	1	1	0	1	1	0	1	1	1	1	1	79
995				1	0	0	1	1	1	1	0	1	0	1	0	58
996				1	1	1	1	0	0	0	0	0	1	0	1	37
997				0	0	1	0	1	0	1	0	1	0	0	0	33
998				1	1	1	1	1	0	1	0	0	0	0	1	45
999				0	0	0	0	1	0	1	1	1	1	0	1	52
1000				0	1	0	0	1	1	1	0	1	1	1	0	64
1001				0	1	0	1	1	0	1	1	1	1	0	1	65
1002				0	1	1	1	0	1	0	0	0	0	1	0	37
1003				0	0	1	1	1	1	1	0	1	0	1	1	64
1004				1	0	0	1	0	1	0	0	0	1	0	0	30
1005				1	1	0	1	0	0	1	1	0	1	1	1	60
1006				1	0	0	1	0	1	1	0	1	0	1	1	54
1007				1	1	0	1	1	1	1	0	1	1	0	0	65
1008				0	1	1	0	0	1	1	0	1	0	0	0	41
1009				1	0	0	0	0	0	1	0	0	1	1	0	33
1010				1	0	0	1	1	0	1	1	1	1	0	1	62
1011				1	1	1	1	0	0	1	1	1	0	1	0	60
1012				0	1	1	1	1	0	0	0	1	1	1	1	62
1013				0	0	0	0	1	1	1	0	1	0	1	0	48
1014				0	1	1	0	0	1	0	1	1	1	1	1	66
1015				1	1	1	1	0	1	1	1	0	1	0	1	65
1016				1	1	0	0	0	1	1	0	0	0	1	0	40
1017				1	1	0	0	1	0	0	1	1	1	0	0	49
1018				1	1	1	1	0	0	0	1	1	1	1	1	66
1019				1	1	0	1	1	1	0	0	1	1	1	0	66
1020				0	1	1	1	0	1	0	1	1	1	1	1	71
1021				0	1	1	1	0	0	0	0	0	1	1	1	42
1022				1	0	1	0	1	0	1	1	0	1	1	0	56
1023				1	0	1	0	0	1	1	1	0	0	1	0	47
1024				1	0	0	1	1	1	0	1	0	0	0	1	44
1025				1	0	1	0	0	0	1	0	0	1	1	0	37
1026				0	0	1	1	0	1	1	0	1	1	1	0	59
1027				0	0	0	0	1	0	1	0	1	1	1	1	53
1028				1	0	0	1	1	0	0	1	1	1	1	1	63
1029				0	0	0	0	1	1	0	1	0	0	0	1	34
1030				0	1	1	0	0	1	1	0	0	1	0	0	40
1031				1	1	1	0	1	0	0	0	0	0	0	0	25
1032				1	1	1	1	0	0	0	0	1	1	1	1	57
1033				0	1	1	1	1	1	0	0	0	1	1	1	62
1034				1	0	0	1	0	1	1	0	0	0	1	1	44
1035				1	0	1	1	1	0	1	1	1	1	0	0	62
1036				1	1	1	1	0	1	0	0	1	1	1	0	61
1037				1	0	0	1	0	0	0	1	0	1	1	0	39
1038				1	0	1	0	1	0	0	0	0	1	0	1	33
1039				0	0	0	1	1	1	1	1	0	1	1	1	68
1040				1	0	1	0	1	1	0	0	1	1	1	1	63
1041				1	0	0	1	1	0	0	0	0	0	1	0	29
1042				0	0	1	1	1	1	1	1	0	0	0	1	53
1043				1	0	1	0	0	1	0	1	1	0	1	1	53
1044				1	0	0	1	1	1	1	0	1	1	0	0	58
1045				0	1	0	0	0	0	1	0	0	1	0	1	31
1046				1	1	0	0	0	0	0	1	0	1	1	0	40
1047				0	0	0	0	0	1	1	1	1	0	0	0	38
1048				0	1	0	1	0	1	1	0	1	1	1	1	66
1049				1	0	0	0	1	0	0	0	1	0	0	0	23
1050				1	0	1	0	1	0	0	1	0	0	1	0	37
1051				0	1	1	0	1	1	0	1	0	0	0	1	45
1052				1	0	0	1	0	0	1	1	1	1	0	1	53
1053				1	1	0	0	1	1	0	1	0	1	0	0	49
1054				1	1	0	1	0	0	1	1	1	0	1	1	60

1055				1	0	0	0	0	0	0	1	0	0	1	1	29
1056				0	1	0	0	0	0	0	1	1	1	0	0	36
1057				0	1	0	1	0	1	0	0	0	1	1	0	42
1058				1	0	0	0	0	0	0	1	1	1	0	0	33
1059				1	0	1	0	0	0	1	0	1	1	0	1	43
1060				1	0	0	1	0	0	0	0	1	0	0	1	25
1061				1	0	1	1	1	1	0	0	0	0	0	0	34
1062				1	0	1	1	0	1	0	1	0	1	0	1	49
1063				1	1	0	0	0	0	0	1	1	0	1	0	40
1064				1	1	0	0	1	0	0	0	1	1	0	0	40
1065				1	1	0	0	0	0	0	0	0	1	1	1	36
1066				1	0	0	1	1	1	0	1	0	0	0	0	38
1067				0	1	0	1	0	0	1	0	1	1	0	0	41
1068				0	1	1	0	0	0	1	1	1	1	1	0	59
1069				0	1	0	0	0	1	0	1	0	1	0	1	41
1070				1	1	1	0	1	0	0	0	0	0	1	0	35
1071				1	0	0	0	1	1	0	1	1	1	0	0	52
1072				0	0	1	1	1	0	0	0	0	0	0	0	20
1073				0	1	1	0	0	1	0	0	1	0	1	0	42
1074				0	0	0	1	1	0	1	1	1	0	1	1	58
1075				1	1	1	0	0	0	0	1	1	1	1	1	60
1076				1	0	0	0	0	0	1	0	0	0	0	1	18
1077				1	1	1	0	0	0	0	0	1	0	1	0	36
1078				1	0	1	1	1	0	0	1	1	0	1	1	58
1079				0	1	0	1	0	1	1	1	1	0	0	0	51
1080				0	0	0	1	0	0	0	0	1	0	1	1	31

QualiPresc - Calidad de la Prescripción en Atención Primaria de Salud

Instrucciones
1. Seleccione el indicador en la Tabla a continuación; y 2. Señalar con una X, en la Tabla de la derecha, el mes en que comienza una nueva mediana.

Indicadores
1. Fecha de nacimiento del paciente
2. Identificación del prescriptor
3. Registro de informe de alergia
4. Medicamento incluido en la lista institucional oficialmente aprobada
5. Principio activo
6. Concentración
7. Dosis
8. Forma farmaceutica
9. Vía de administración
10. Frecuencia de administración
11. Duración del tratamiento
12. Informaciones sobre el uso del medicamento
13. Recomendaciones no farmacológicas
14. CALIDAD

Mes	Valor	Mediana	Nueva Mediana
jan/21			
fev/21			
mar/21			
abr/21			
mai/21			x
jun/21			
jul/21			
ago/21			
set/21			
out/21			x
nov/21			
dez/21			x
jan/22			
fev/22			
mar/22			
abr/22			
mai/22			
jun/22			
jul/22			
ago/22			
set/22			
out/22			
nov/22			
dez/22			
jan/23			
fev/23			
mar/23			
abr/23			
mai/23			
jun/23			
jul/23			
ago/23			
set/23			
out/23			
nov/23			
dez/23			

14. CALIDAD

Run Chart: 14. CALIDAD

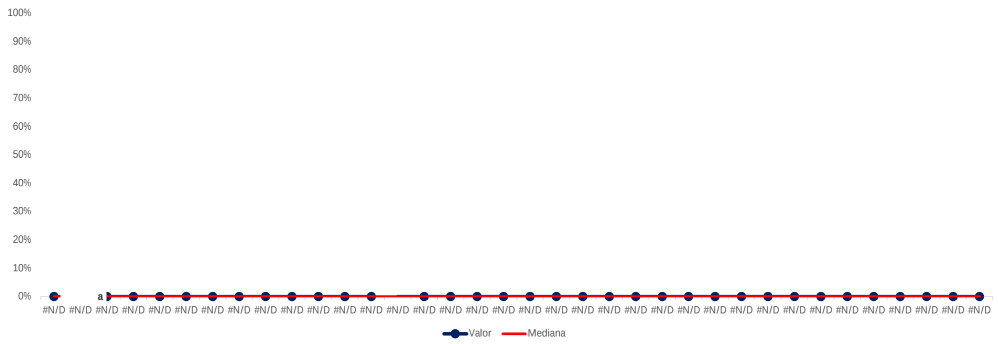

Supplement: S3 File — (PDF) [file pone.0267707.s006.pdf]
