# Supplementary material for: Validation of the QualiPresc instrument for assessing the quality of drug prescription writing in primary health care
Source: PLoS One. 2022 May 11;17(5):e0267707. doi: 10.1371/journal.pone.0267707 (PMC9094502; doi:10.1371/journal.pone.0267707)
Supplement: Attachment — Submitted filename: Plos one 202201.docx [file pone.0267707.s007.docx]

This is a study whose novelty is relatively clear, but there were some points which makes this manuscript difficult to understand.

1. (Abstract, line 28) What “writing criteria” mean? Is this a commonly used phrase?
2. (Abstract, Methods) Please write about where this study was taken place, who are the target patients or medical institutes of the study, and their numbers.
3. (Methods, line 105) How did you create QualiPresc ? Does “Step1. Validity analysis” correspond to the method of creating QualiPresc ? In that case, it is bizarre that how the QualiPresc was created is written in the section of validation analysis. In other words, you should write separately about development and validation of QualiPresc.
4. (Results) All the Figures are blurred, and difficult to see. Please increase the quality of the Figures. You should check it before submission. In addition, Figures 2-4 can be presented by Tables. Why did you present them in Figures ?
5. (Results, Figure 4) Does Figure 4 corresponds to the finalized QualiPresc ? Please write more about how to use it in practice.
6. (Discussion) As you write in the limitations, the generalizability of QualiPresc may be limited. For example, it might be good to use it in other countries. Therefore, you should write about where this method can be applicable in the Conclusions and Conclusions section of Abstract.
