# Supplementary material for: Validation of the QualiPresc instrument for assessing the quality of drug prescription writing in primary health care
Source: PLoS One. 2022 May 11;17(5):e0267707. doi: 10.1371/journal.pone.0267707 (PMC9094502; doi:10.1371/journal.pone.0267707)
Supplement: Attachment — Submitted filename: Response to Reviewers.doc [file pone.0267707.s009.doc]

**COVER LETTER WITH A POINT-BY-POINT RESPONSE TO THE COMMENTS**

Manuscript ID: PONE-D-21-37829R1

Manuscript Title: VALIDATION OF THE QUALIPRESC INSTRUMENT FOR ASSESSING THE QUALITY OF DRUG PRESCRIPTION WRITING IN PRIMARY HEALTH CARE

Dear Editor and Reviewers,

We appreciate your thoughtful review of our manuscript.

We have reviewed and addressed each of the reviewers’ comments, as outlined below. Changes to the manuscript have been marked.

| **Journal Requirements** | **Authors' Response** |
| --- | --- |
| Please review your reference list to ensure that it is complete and correct. If you have cited papers that have been retracted, please include the rationale for doing so in the manuscript text, or remove these references and replace them with relevant current references. Any changes to the reference list should be mentioned in the rebuttal letter that accompanies your revised manuscript. If you need to cite a retracted article, indicate the article’s retracted status in the References list and also include a citation and full reference for the retraction notice. | None of the papers cited in this manuscript were retracted. |
| **Reviewer #1** |  |
| 6. Review Comments to the Author  1. (Tables) I think Tables 1-3 need to be shown in the main manuscript, not in supplementary information. | 1. We agree. Tables 1-3 will be in the manuscript. They are not Supplementary Information. The current view at the end of the manuscript is only during the review process, as this is Plos One's organization during this process (tables and figures). We have included the titles of all tables and figures in the text to indicate the approximate location where they will be inserted. See manuscript template ([Download sample manuscript body (PDF)](https://journals.plos.org/plosone/s/file?id=9cba/PLOS Manuscript Body Formatting Guidelines.pdf)). |
| 2. (Discussion, lines 363) It is bizarre that the contents of QualiPresc are shown for the first time in Discussion. You should indicate it in the Methods or Results. | 2. Changes made. This information is presented in the Results topic (lines 277-282). |
| 3. In addition, QualiPresc in English version needs to be written in English, but not in another language. | 3. Changes made. View Supplementary Files 1-3. |
